# Ancestral regulatory mechanisms specify conserved midbrain circuitry in arthropods and vertebrates

**DOI:** 10.1073/pnas.1918797117

**Published:** 2020-08-03

**Authors:** Jessika C. Bridi, Zoe N. Ludlow, Benjamin Kottler, Beate Hartmann, Lies Vanden Broeck, Jonah Dearlove, Markus Göker, Nicholas J. Strausfeld, Patrick Callaerts, Frank Hirth

**Affiliations:** ^a^Department of Basic and Clinical Neuroscience, Institute of Psychiatry, Psychology and Neuroscience, King’s College London, London SE5 9RT, United Kingdom;; ^b^Department of Basic and Clinical Neuroscience, Maurice Wohl Clinical Neuroscience Institute, Institute of Psychiatry, Psychology and Neuroscience, King’s College London, London SE5 9RT, United Kingdom;; ^c^University of Basel, 4031 Basel, Switzerland;; ^d^Laboratory of Behavioural & Developmental Genetics, Department of Human Genetics, Katholieke Universiteit Leuven, 6023000 Leuven, Belgium;; ^e^Leibniz Institute German Collection of Microorganisms and Cell Cultures, 38124 Braunschweig, Germany;; ^f^Department of Neuroscience, University of Arizona, Tucson, AZ 85721;; ^g^Center for Insect Sciences, University of Arizona, Tucson, AZ 85721

**Keywords:** brain, evolution, neural circuit, gene regulatory network, homology

## Abstract

Corresponding attributes of neural development and function suggest arthropod and vertebrate brains may have an evolutionarily conserved organization. However, the underlying mechanisms have remained elusive. Here, we identify a gene regulatory and character identity network defining the deutocerebral–tritocerebral boundary (DTB) in *Drosophila*. This network comprises genes homologous to those directing midbrain-hindbrain boundary (MHB) formation in vertebrates and their closest chordate relatives. Genetic tracing reveals that the embryonic DTB gives rise to adult midbrain circuits that in flies control auditory and vestibular information processing and motor coordination, as do MHB-derived circuits in vertebrates. DTB-specific gene expression and function are directed by *cis*-regulatory elements of developmental control genes that include homologs of mammalian *Zinc finger of the cerebellum* and *Purkinje cell protein 4*. *Drosophila* DTB-specific *cis*-regulatory elements correspond to regulatory sequences of human *ENGRAILED-2, PAX-2*, and *DACHSHUND-1* that direct MHB-specific expression in the embryonic mouse brain. We show that *cis*-regulatory elements and the gene networks they regulate direct the formation and function of midbrain circuits for balance and motor coordination in insects and mammals. Regulatory mechanisms mediating the genetic specification of cephalic neural circuits in arthropods correspond to those in chordates, thereby implying their origin before the divergence of deuterostomes and ecdysozoans.

## Significance

Comparative developmental genetics indicate insect and mammalian forebrains form and function in comparable ways. However, these data are open to opposing interpretations that advocate either a single origin of the brain and its adaptive modification during animal evolution; or multiple, independent origins of the many different brains present in extant Bilateria. Here, we describe conserved regulatory elements that mediate the spatiotemporal expression of developmental control genes directing the formation and function of midbrain circuits in flies, mice, and humans. These circuits develop from corresponding midbrain-hindbrain boundary regions and regulate comparable behaviors for balance and motor control. Our findings suggest that conserved regulatory mechanisms specify cephalic circuits for sensory integration and coordinated behavior common to all animals that possess a brain.

Many components of the vertebrate and arthropod forebrain share features regarding neural arrangements along the rostrocaudal axis and their connections of sensory and motor pathways with higher integrative centers. This is exemplified in ancestral vertebrate lineages from which lampreys and hagfish derived. Rostral neuropils of the forebrain encode visual and olfactory information that is relayed to further forebrain centers integrating these modalities (1, 2). Circuits involved in vestibular reception and integration, and by extension acoustic perception, develop in more caudal territories of the anterior brain that arise in the telencephalon of vertebrates (3). Corresponding arrangements are found in the deutocerebrum of arthropods (4), among which Onychophora offer comparable proxies of ancestral neural arrangements (5).

The formation of these neural arrangements is mediated by conserved developmental control genes acting along anterior-posterior (AP) and dorso-ventral (DV) axes of the embryonic nervous system (6–13). For example, the *Drosophila* gene *orthodenticle* (*otd*) and its mammalian *Otx* homologs are required in both for rostral brain development (14, 15). In cross-phylum rescue experiments, human *OTX2* restores fly brain formation in *otd* mutant embryos (16, 17) while fly *otd* can replace *Otx1/2* in mouse head and forebrain formation (18, 19). Fly *engrailed* can replace *Engrailed-1* in mouse midbrain-hindbrain boundary (MHB) development (20). Further cross-phyletic studies revealed correspondences in developmental genetic mechanisms underlying circuit formation and information processing of the vertebrate basal ganglia and the arthropod central complex, including pathologies (9, 21–23). These similarities also extend to comparisons of the vertebrate hippocampus and the arthropod mushroom bodies, forebrain centers that support spatial navigation, allocentric memory, and associative learning (10, 24).

Based on these findings, it has been postulated that the corresponding brain organization in arthropods and vertebrates is an example of a genealogical relationship that can be traced to a distant pre-Cambrian ancestor (8, 24, 25). Indeed, evidence from soft tissue preservation in fossils of stem arthropods suggests that the gross cerebral arrangements present in the four extant panarthropod lineages originated prior to the early Cambrian. This further implies that neural ground patterns attributed to the Panarthropoda may be both ancient and extremely stable over geological time (25). Here, ground patterns are defined as ancestral arrangements that are inherited with modification. However, due to the extreme rarity of such detailed fossil material, resolving correspondences across phyla has to rely instead on the identification of shared developmental rules and their outcomes (21, 24). These correspondences are expected to be defined by common gene regulatory (26) and character identity networks (27) that convey positional information and identity to a species-specific morphology, albeit often highly derived (28). Accordingly, it is the comparative analysis of ground patterns across phylogenetic lineages that allows the identification of correspondences among cell types, tissue, and organs and that informs about their origins and genealogical relationships (29).

We applied this approach to compare the formation and function of the *Drosophila* and vertebrate MHB region. The vertebrate MHB is positioned by adjacent *Otx* and *Gbx* activity along the AP axis and elaborated by region-specific expression of *Engrailed*, *Wnt*, *Pax2/5/8*, and FGF8-mediated organizer activity (30–33). In *Drosophila*, the corresponding boundary (henceforth referred to as the deutocerebral–tritocerebral boundary [DTB]) is defined by adjoining expression of *otd* and *unplugged* (*unpg*)*,* homologs of *Otx* and *Gbx*, respectively (34). The observation of these similar expression patterns raises a number of questions: whether they reflect a shared developmental program for the MHB and DTB; what adult brain structures derive from them; and what their function might be. We hypothesized that, if the DTB evolutionarily corresponds to the vertebrate MHB, its formation would be mediated by gene regulatory and character identity networks homologous to those driving MHB formation. Furthermore, if true, we expected the DTB to give rise to circuits mediating similar behaviors controlled by MHB-derived circuitry. Here, we describe experimental evidence verifying that the arthropod DTB indeed shares a ground pattern organization with the vertebrate MHB, including correspondence of neural circuits and their behavioral functions.

## Results

**Fig. 1.**
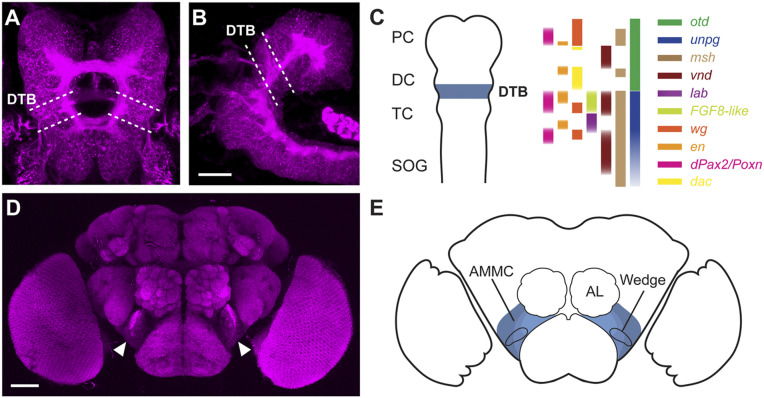
The embryonic deutocerebral–tritocerebral boundary gives rise to the antennal mechanosensory motor center of the adult brain in *Drosophila*. (*A*, *B*, and *D*) Confocal images of stage 14 embryonic (*A*, dorsal; *B*, lateral) and adult brain (D, frontal) immunolabeled with anti-Brp/nc82; dashed lines demarcate the deutocerebral–tritocerebral boundary (DTB) region; arrowheads indicate antennal mechanosensory motor center (AMMC). (*C*) Schematic summarizing gene expression patterns delineating the DTB in the embryonic brain, including the *dachshund* (*dac*) regulatory element R65A11. DC, deutocerebrum; PC, protocerebrum; SOG, subesophageal ganglion; TC, tritocerebrum. (*E*) Schematic of adult brain showing AMMC, Wedge, and antennal lobes (AL). (Scale bars: *B*, 20 μm; *D*, 50 μm.)

We focused on phylotypic (35) stage 11 to 14 embryos (Fig. 1 *A* and *B*) to characterize morphological and molecular signatures of the developing *Drosophila* DTB. In addition to the adjoining *otd* and *unpg* expression and function reported earlier (34), we found specific domains of expression of the Pax2/5/8 homologs *shaven(sv)/dPax2* and *Pox neuro* (*Poxn*), as well as *engrailed* (*en*), *wingless* (*wg/dWnt*), *muscle specific homeobox* (*msh/dMsx*), *ventral nervous system defective* (*vnd/dNkx2*), and *empty spiracles* (*ems/dEmx*) (Fig. 1*C* and *SI Appendix*, Fig. S1*A*). For axial patterning, we examined the expression and function of the key genes *otd* + *wg* (antero-posterior) and *msh* + *vnd* (dorsal-ventral), which revealed essential roles in DTB formation (*SI Appendix*, Fig. S1*B*).

**Fig. 2.**
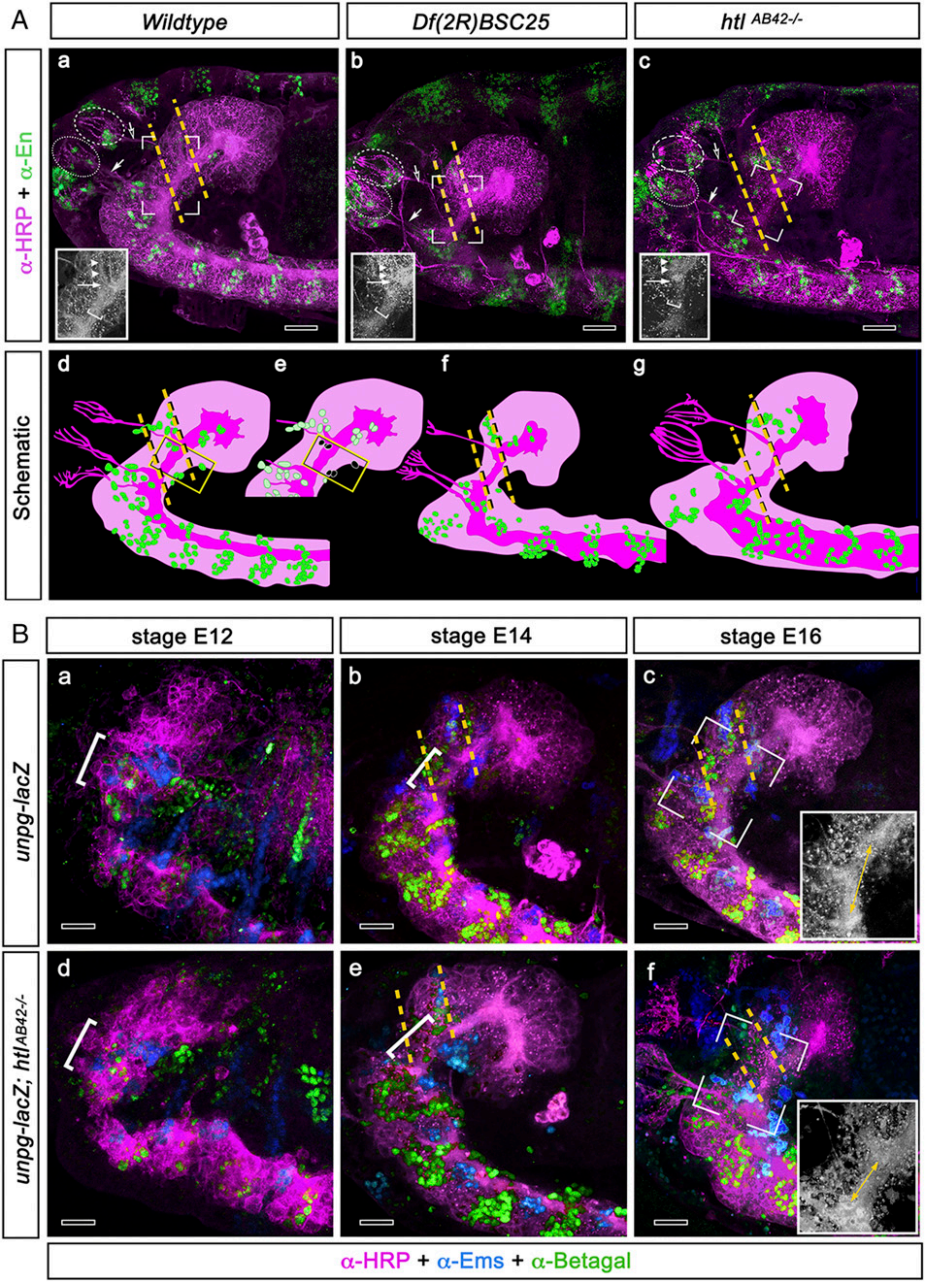
FGF8 signaling is required for the formation and maintenance of the embryonic DTB. (*A*, *a*–*c*) Confocal images of stage 13/14 embryonic brains immunolabeled for HRP (magenta) and anti-Engrailed (green/yellow). To assist orientation across examples, corresponding axon bundles of developing deutocerebral and tritocerebral sensory nerves (open and closed arrows) and their developing sensory neurons (circled) are shown for the wild type (*a*), *Df(2R)BSC25* (*b*), and *htl*^*AB42−/−*^ (*c*). These nerves eventually reside in the antenna, which in the imago is equipped with both deutocerebral olfactory neurons and mechanosensory neurons of both deuto- and tritocerebral origin. Dashed yellow lines in *a*–*c* indicate the deutocerebral–tritocerebral boundary (DTB). *Insets* in *a*–*c* refer to open rectangles in each panel indicating corresponding normal (*a*) and affected morphologies of the DTB region (*b* and *c*). These include the prefrontal commissure (arrowheads), the width and integrity of longitudinal connectives (white brackets), and axonal projections at the bifurcation between prefrontal commissure and longitudinal connective (white arrow). All elements are altered in *Df(2R)BSC25* and *htl*^*AB42−/−*^. Arrowheads and arrow mark the positions where these elements should normally be positioned in the wild-type condition. (*d*–*g*) Corresponding color-coded schematics highlight the distribution of engrailed-expressing neurons (green); those indicated within a yellow rectangle in wild type (*d* and removed in the schematic *e*) are absent in homozygous *Df(2R)BSC25* (*b* and *f*) and *htl*^*AB42−/−*^ (*c* and *g*). The morphology of the DTB region in the *Df(2R)BSC25* mutant, a deficiency deleting both *FGF8-like1* and *FGF8-like2* genomic loci, reveals morphological alterations at the DTB (yellow dashed lines) when compared to the wild type (*a*, *d*, and *e*). Likewise, homozygous *htl*-null mutant embryos where DTB-specific engrailed expression is lost (*c* and *g*) reveal morphological alterations at the DTB (yellow dashed lines) when compared to wild type (*a*, *d*, and *e*). (*B*) Lateral views of developing embryos at stages E12, E14, and E16. Progressive unpg-lacZ and ems expression patterns reveal successive formation of the DTB. Panels *a*–*c* illustrate transgenic unpg-lacZ in control brains at embryonic stages E12, E14, and E16 showing progressive maturation of the DTB domain (bracketed), enlarged in the *Lower Right Inset* of *c*. (*d*–*f*) Maintenance of DTB depends on FGF8 signaling: as illustrated by the successive developmental stages of *htl*-null mutant embryos (*unpg-lacZ; htl*^*AB42−/−*^), in which unpg and ems expression patterns are initially visible but those relating to the DTB are subsequently altered by embryonic stage 16. (*Insets* in *c* and *f*) Monochrome enlargements of the open boxed areas in *a* and *f* resolve rostrocaudal shortening in the mutant (double arrows insect *f*) compared with the DTB of the wild type (*Inset*, *c*). HRP immunolabeling is shown as magenta; anti-Ems is shown blue; and anti–β-galactosidase is shown green. For *Insets* in *B, c* and *f*, the magnification is 40×. (Scale bars: 10 μm.)

A cardinal feature of the vertebrate MHB is its organizer activity, mainly mediated by the FGF8 effector molecule (30–33, 36). Previous studies failed to identify a DTB-related function of the FGF8 homolog *branchless* and its receptor *breathless* in embryonic brain development of *Drosophila* (34). We now show that a second set of FGF8-like orthologs, *thisbe/FGF8-like1* and *pyramus/FGF8-like2* (37, 38), and the FGF8 receptor *heartless* (*htl*) are expressed at the DTB (*SI Appendix*, Fig. S2). Consistent with earlier reports (37, 38), phylogenetic comparison of annotated protein sequences reveals that Pyramus and Thisbe, like Branchless, are homologs of human and mouse FGF8, FGF17, and FGF18, as well as of the ascidian *Ciona intestinalis* FGF8/17/18, the Annelid *Capitella teleta* FGF, and the Cnidarian *Nematostella vectensis* FGF8a (*SI Appendix*, Fig. S3). Similarly, phylogenetic comparison of annotated protein sequences reveals that the *Drosophila* FGF receptors Breathless and Heartless are homologs of human and mouse FGF receptors FGFR1-FGFR4, as well as of the ascidian *C. intestinalis* FGFR, the annelid *C. teleta* FGFR, and the cnidarian *N. vectensis* FGFR (*SI Appendix*, Fig. S4). A functional role for FGF8 signaling at the *Drosophila* DTB was revealed by altered *engrailed* expression patterns and morphological defects in embryos homozygous for a deficiency, *Df(2R)BSC25*, uncovering both *FGF8-like1* and *FGF8-like2* genomic loci, and of an *htl*-null allele, *htl*^*AB42−/−*^ (Fig. 2*A*). As indicated in the *Insets* of Fig. 2 *A*, *a*–*c*, these defects impact the prefrontal commissure (indicated by arrowheads) and the size and integrity of longitudinal connectives (indicated by the white brackets), as well as axonal projections, also indicated by the white arrow in each panel at the bifurcation between prefrontal commissure and longitudinal connective. These observations were further substantiated by progressive changes and loss of the DTB expression patterns of *unpg* and *ems* in *htl*-null mutant embryos, between embryonic stages 12 to 16 (Fig. 2 *B*, *d*–*f*; compare to panels *a*–*c*). These data identify a role of FGF8-like signaling in the maintenance of the embryonic DTB region in *Drosophila*. Given the expression pattern of FGF8-like homologs in the brain and procephalic endoderm (*SI Appendix*, Fig. S2), it remains to be shown whether cell autonomous or cell nonautonomous FGF8 signaling is required for DTB formation and maintenance. Ectopic expression of the FGF8-like homolog *thisbe* in *ems*-specific brain regions did not cause any detectable changes in morphology or molecular signatures of the DTB region (*SI Appendix*, Fig. S5). Despite conserved regulatory interactions between *otd/Otx* and *unpg/Gbx* (34), these data indicate the absence of FGF8-mediated organizer activity in the embryonic DTB. We conclude that direct or indirect FGF8-like signaling is required for the maintenance of the embryonic DTB but, contrary to what is seen in vertebrate MHB development, appears not to organize the DTB region in *Drosophila*.

To identify the adult brain structures and associated functional modalities that derive from the embryonic DTB, we utilized genetic tracing of neural lineages employing the tracer line *UAS-mCD8::GFP, tub-FRT-CD2-FRT-Gal4, UAS-FLP/CyO GMR Dfd YFP* (39, 40). We first deployed *en-Gal4* as driver, which, combined with the tracer line, causes permanent GFP labeling of progeny of *engrailed*-expressing cells, even when those progeny themselves no longer express *engrailed*. This is due to the fact that the genetic tracer line carries a flippase (FLP) recombinase-related flip-out cassette, in which a strong and ubiquitous *tubulin* enhancer is separated from a Gal4 transcriptional activator by CD2, flanked by a pair of FRT sites, thereby preventing Gal4 expression. As soon as the genetic tracer line is crossed with an independent Gal4 driver, in this case *en-Gal4*, it initiates UAS-FLP expression as part of the genetic tracer, which in turn induces recombination at the CD2-flanking FRT sites. As a result, the CD2 element is excised, resulting in a fusion of the *tubulin* enhancer to drive Gal4, which in turn leads to expression of *UAS-mCD8::GFP* in that cell and all of its progeny. Since *tubulin* is constitutively active, the genetic tracing thus permanently marks targeted cells and their progeny, thereby revealing lineage-relationship even if some of the traced cells no longer express *engrailed*.

**Fig. 3.**
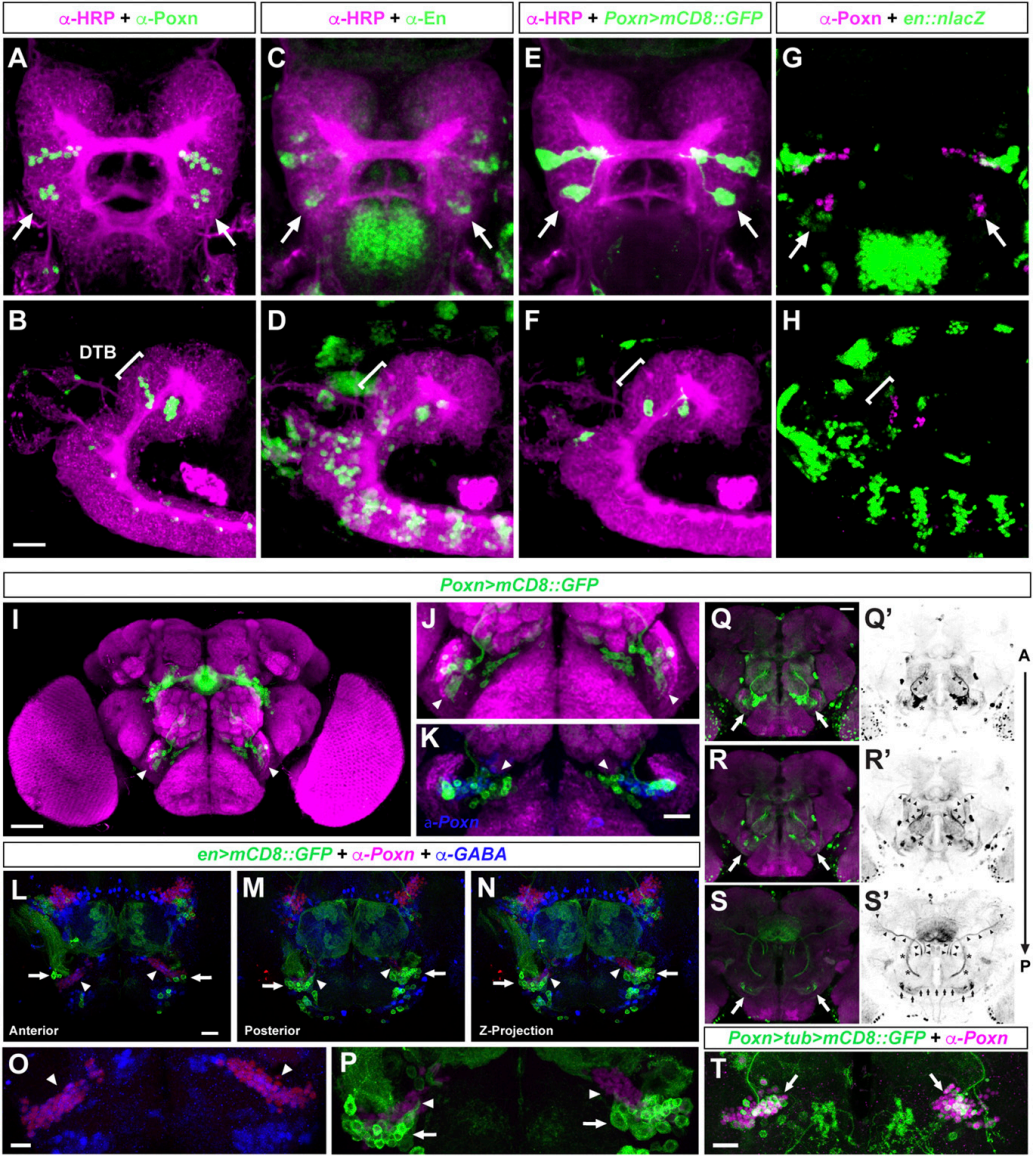
DTB and AMMC/Wedge-specific expression of the Pax2/5/8 homolog *Pox neuro.* Confocal images of embryonic stage 14 (*A*–*H*); anterior is up in *A*, *C*, *E*, and *G*, dorsal views; anterior is to the *Left* in (*B*, *D*, *F*, and *H*, lateral views. (*A* and *B*) in the anterior embryonic brain, anti-Pox neuro (Poxn) immunolabeling reveals two Poxn expression domains, an anterior at the protocerebral–deutocerebral neuromere boundary and a posterior demarcating the deutocerebral–tritocerebral boundary (DTB) region (arrows, bracket). (*C* and *D*) engrailed expression demarcates neuromere boundaries, including the DTB (arrows, bracket). (*E* and *F*) *Poxn > mCD8::GFP* expression reveals GFP expression pattern comparable to endogenous Poxn expression (compare with *A* and *B*), including the DTB (arrows; bracket). (*G* and *H*) *en > nLacZ* expression reveals Engrailed expression pattern comparable to endogenous En expression (compare with *C* and *D*), including the DTB that encompasses the Poxn expression domain (arrows; bracket). (*I*–*T*) Confocal images of adult brain; dorsal is up. (*I*–*K*) *Poxn*^*brain*^ *> mCD8::GFP*-mediated cell labeling identifies Poxn+ cell clusters (green, arrowheads) in close vicinity to the antennal mechanosensory motor center (AMMC), the majority of which are anti-Poxn immunopositive (*K*, in blue). (*L*–*P*) *en > mCD8::GFP* visualizes AMMC neurons (arrows) that are located in close vicinity/adjacent to Poxn-positive cells (magenta, arrowheads; enlarged views in *O* and *P*) that are immunoreactive for anti-GABA (*O*, blue, arrowheads) like En-expressing cells (*P*, arrows). (*Q*–*S*’), *Poxn*^*brain*^ *> mCD8::GFP* visualizes AMMC/Wedge neurons (arrows) and their projections to antennal glomeruli (*Q* and *Q’*) to ventrolateral protocerebrum (*R*–*S’*, middle section of brain), as well as commissural axons of AMMC/Wedge neurons (*S’*, small arrows). (*T*) *Poxn > tub > mCD8::GFP*-mediated genetic tracing of DTB Poxn lineages identifies AMMC/Wedge neurons (arrows). (Scale bars: *B*, *K*, *L*, and *O*, 20 μm; *Q*, *T*, 25 μm; *I*, 50 μm.)

Genetic tracing of *engrailed*-expressing lineages of the embryonic neuroectodermal DTB (*SI Appendix*, Fig. S6 *A*–*C*) identified neurons and projections of the antennal mechanosensory and motor center (AMMC) and select antennal glomeruli in the adult brain (Fig. 1 *D* and *E* and *SI Appendix*, Fig. S6 *D*–*F*). We also determined the fate of Poxn-expressing cells, which in the embryonic brain are located next to DTB-specific engrailed lineages (Fig. 3 *A*–*H*). In the adult brain, Poxn-expressing cells are present in a caudal volume of the deutocerebrum called the Wedge receiving auditory and gravity sensing afferents (41), where its interneurons, as do En-positive cells, express the neurotransmitter GABA (Fig. 3 *I*–*P*). Genetic tracing revealed that the Poxn-expressing AMMC/Wedge neurons derive from DTB lineages. Furthermore, their region-specific projection patterns resemble the mechanosensory pathway architecture of local interneurons and projection neurons (Fig. 3 *Q*–*T*) previously identified for the AMMC and the Wedge (41–44).

The *Drosophila* AMMC and Wedge neuropils comprise neurons that mediate auditory, vestibular, mechanosensory, and somatosensory information processing in pathways with similarities to the mammalian auditory and vestibular pathways (41–44). In vertebrates, auditory, vestibular, somatosensory, and motor information are processed by neural populations of the tectum and cerebellum, adult brain structures derived from the MHB region (36, 45). These fate-mapping studies in mice did not identify specific neural circuits within the tectum and cerebellum, which limits a structural comparison until more refined studies at cell and circuit resolution in vertebrates become available.

**Fig. 4.**
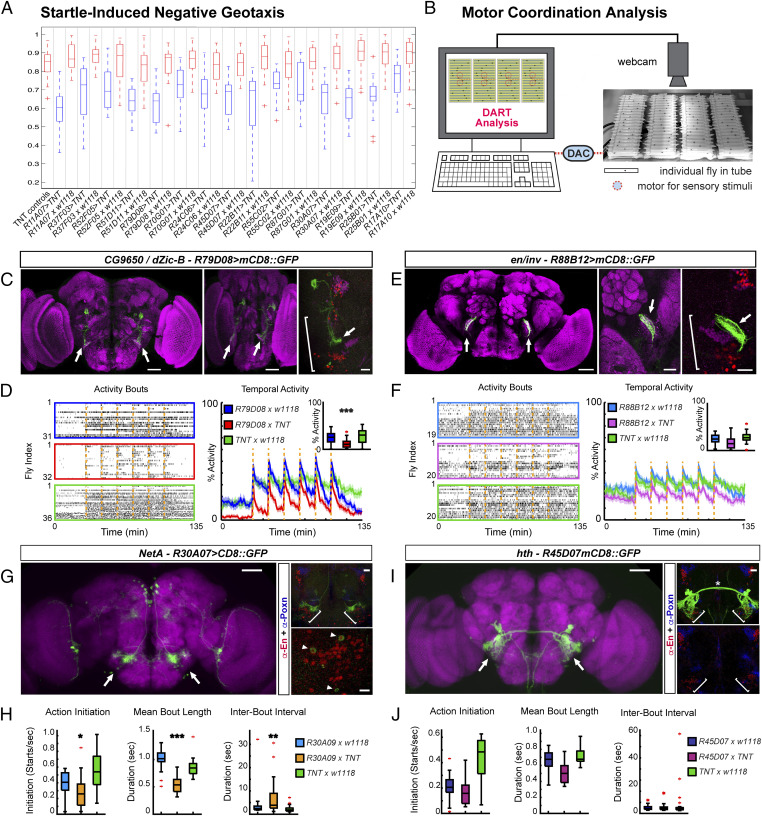
The AMMC mediates motor coordination in *Drosophila*. (*A*) Startle-induced negative geotaxis of AMMC-specific GAL4 lines misexpressing *UAS*-*TNT* and controls (*n* = 150 each). (*B*) *Drosophila* Arousal Tracking (DART) recording each fly in tube walking back and forth, motors underneath elicit vibration stimuli via digital-to-analog converter (DAC). (*C*) *CG9560/dZic-B, R79D08 > mCD8::GFP* immunolabeled with anti-Brp/nc82 and anti-GFP visualizes AMMC-specific (bracket) arborizations (arrows). (*Middle*) Rotated brain to depict AMMC-specific projections. (*D*) Motor behavior of *R79D08 > TNT,* UAS/+ and Gal4/+ control flies. (*Left*) Raster plots of activity bouts; each lane one individual fly; colored boxes indicate genotypes. (*Right*) Stimulus response (main plot) and median response (*Inset*) to repeated mechanical stimulation (dashed orange lines). (*E*) *inv R88B12 > mCD8::GFP* visualizes neuronal projections to AMMC (arrows, enlarged for hemisphere in *Middle* and *Right*) encompassed by anti-En (green) and anti-Poxn (magenta) expression domains (bracket). (*F*) Motor behavior of *R88B12 > TNT,* UAS/+ and Gal4/+ control flies, parameters as in *D*. (*G*) *NetA*, *R30A07 > mCD8::GFP* expression in AMMC (*Left*, arrows). (*Right*) Anti-En (red) and anti-Poxn (blue) immunolabeling encompasses *R30A07 > mCD8::GFP* domain (brackets) and cells, some coexpressing Engrailed (arrowheads). (*H*) Motor kinematics of *R30A07 > TNT,* UAS/+ and Gal4/+ control flies. (*I*) *hth R45D07* > m*CD8::GFP* in AMMC (arrows). (*Right*) *R54D07 > mCD8::GFP* visualizes AMMC interneurons and dendritic arborizations close to anti-Engrailed (red) and anti-Poxn (blue) immunolabeled neurons that encompass AMMC/Wedge (brackets). (*J*) Motor kinematics of *R45D07 > TNT,* UAS/+ and Gal4/+ control flies. Mean ± SEM. *P < 0.05, **P < 0.01, ***P < 0.001. Data of *G*, *Left* and *I*, *Left *are from the Janelia FlyLight database (49), with permission. (Scale bars: *C*, *E*, *G*, *I*, *Left*, 50 μm; *C*, *Middle*, 50 μm; *C*, *Right*, 40 μm; *E*, *Middle Right*, 10 μm; *G*, *Top Right*, 25 μm; *G*, *Bottom Right*, 10 μm; *I*, *Right*, 25 μm.)

Despite these limitations, we reflected on whether functional comparisons might be possible. The tectum and cerebellum receive auditory and vestibular, as well as motor, information and, among other functions, are important for balance, body posture, sensorimotor integration, and motor coordination (1, 4, 46). To test whether DTB-derived circuits in *Drosophila* might exert similar functions, the GAL4-UAS system was used to express *Tetanus-Toxin-Light-Chain* (*TNT*) and inhibit synaptic transmission (47) in subsets of AMMC neurons (42). Flies were tested for their startle-induced negative geotaxis (SING) response, which, after being shaken to the bottom in a test tube, quantifies their ability to right themselves and climb upwards (48). Except *R19E09 > TNT*, all of the tested genotypes showed significantly impaired SING behavior (Fig. 4*A* and *SI Appendix*, Table S1). This includes R79D08, R45D07, and R30A07 that target AMMC neurons and coexpress *engrailed*, or encompass *Poxn* and *engrailed* expression domains (Fig. 4 *C*–*I*). Of note, several of the tested genotypes showed difficulties with balance and to right themselves, as exemplified for *R52F05 > TNT* compared to control (Movies S1 and S2).

To further analyze AMMC-mediated motor coordination, we employed video-assisted motion tracking and recorded freely moving flies (Fig. 4*B*). During 135-min recordings, activity bouts and movement trajectories were analyzed to quantify locomotion parameters: frequency of episodic movements, how often they were initiated, and their length and average velocity, as well as the duration and frequency of pauses. Response to sensory stimulation triggered by repeated mechanical shock was also recorded. *UAS-TNT* expression by *R79D08* (*CG9650/dZic-B*)-Gal4, which targets zone B (41–43) of the AMMC (Fig. 4*C*, arrows), significantly impaired overall activity and duration, with fewer actions initiated, shorter episodes of activity and their intervals, and reduced velocity and distances traveled (Fig. 4*D* and *SI Appendix*, Fig. S7*A*). *UAS-TNT* expression by *R88B12* (*en/inv*)-Gal4 targeting zone A (41–43) of the AMMC (Fig. 4*E*, arrows) significantly impaired average and prestimulus speed, with shorter bouts of activity, together resulting in less distance traveled (Fig. 4*F* and *SI Appendix*, Fig. S7*B*). Comparable motor phenotypes were seen with *R30A07* (*NetA*)-Gal4, which targets AMMC neurons that coexpress *engrailed* (Fig. 4*G*, arrowheads, Fig. 4*H*, and *SI Appendix*, Fig. S7*C*), but not with *R45D07* (*hth*)-Gal4 targeting parts of the AMMC-specific giant fiber system (Fig. 4 *I* and *J* and *SI Appendix*, Fig. S7*D*). Together, with SING data, our behavioral observations establish essential functions of the AMMC for sensorimotor integration (41, 42, 44), balance, righting reflex, and motor coordination in *Drosophila,* behavioral manifestations similar to MHB region-derived circuits in vertebrates.

Our findings thus far establish correspondences between *Drosophila* DTB and vertebrate MHB at multiple levels, including adult brain circuits and the behaviors they regulate. We hypothesized that this will be reflected in commonalities among character identity networks of DTB and MHB that are mediated by homologous gene regulatory networks (27, 28). To test this hypothesis, we screened the Janelia Gal4 collection (49) for *cis*-regulatory elements (CREs) mediating the spatiotemporal expression of developmental genes controlling DTB formation in *Drosophila.* We identified CREs for *msh*, *vnd*, *ems*, and for *thisbe/FGF8-like1* (*SI Appendix*, Fig. S8 *A*–*E*), genes that are essential for the formation and/or maintenance of the embryonic DTB (Fig. 2 and *SI Appendix*, Fig. S1) (14). In addition, we identified CREs for *Wnt10*, *Sex combs reduced/Hox5;* the *Drosophila* homologs of *zinc finger of the cerebellum* (*ZIC*)*, odd-paired* (*opa/dZic-A*) and *CG9650/dZic-B;* of *Purkinje cell protein 4* (*PCP4*), *igloo* (*igl/dPCP4*); of *Ptf1a, Fer1/dPtf1a*, and of *Lim1* (*SI Appendix*, Fig. S8 *F*–*J*). All mammalian homologs of these genes have been implicated in vertebrate MHB formation and the specification of midbrain-cerebellar circuitry (30–34, 50) (*SI Appendix*, Table S2).

**Fig. 5.**
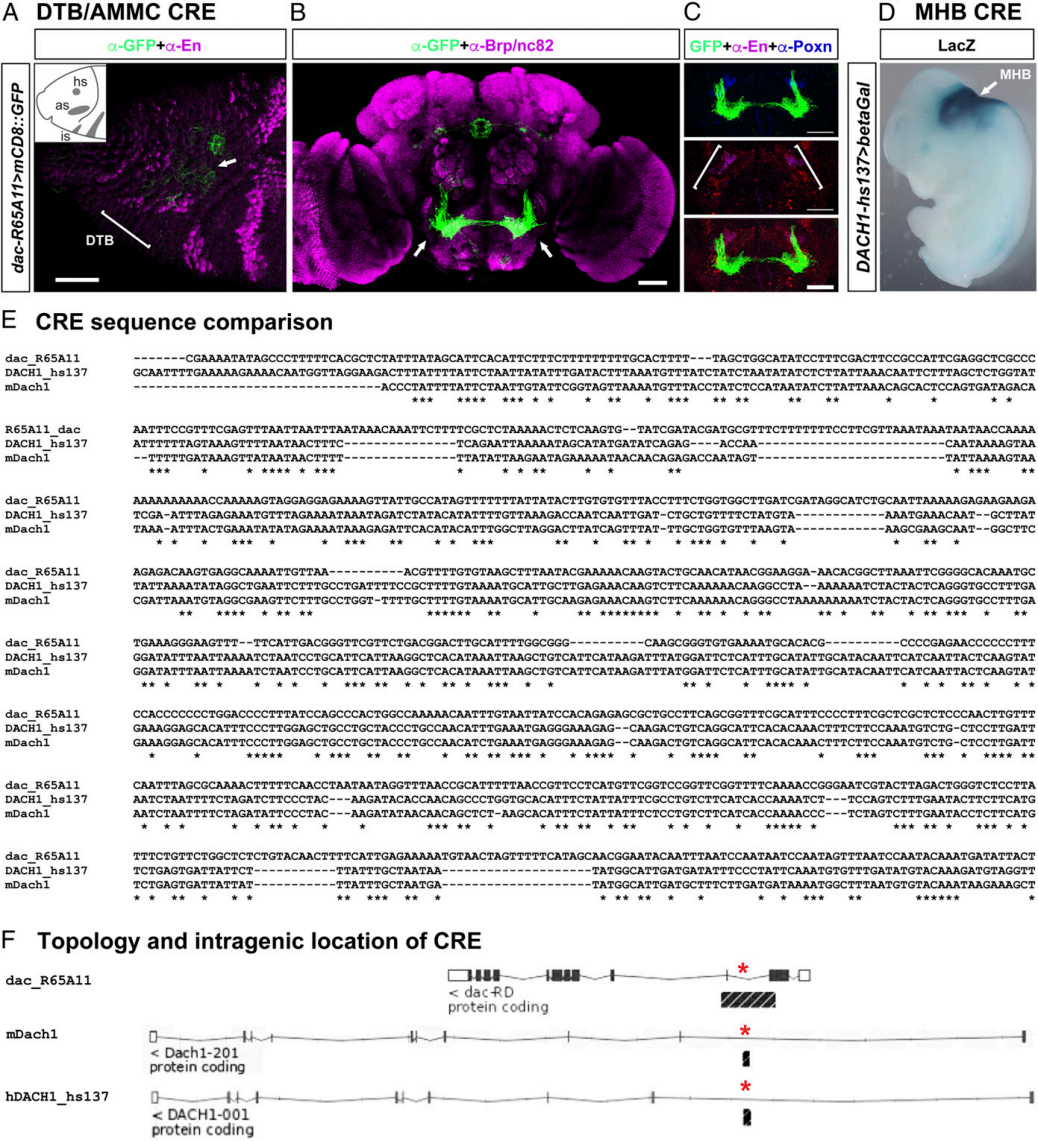
Conserved *cis*-regulatory sequences of *dac/DACH1* direct DTB-AMMC–specific expression in *Drosophila* and MHB-specific expression in mouse. (*A*) Confocal image of stage 10/11 *Drosophila* neuroectoderm of *R65A11-Gal4 > mCD8::GFP* embryo (lateral view, anterior left, dorsal up), immunolabeled with anti-GFP (green) and anti-En (magenta). (*Inset*) Illustrates Engrailed expression domains in procephalic neuroectoderm, including head spot (hs), antennal spot (as), and intercalary spot (is). *R65A11 > GFP* expression (arrow) is seen in DTB primordium (bracket). (*B*) Confocal image of *R65A11-LexA > mCD8::GFP* expression in AMMC (arrows) of adult *Drosophila* brain, immunolabeled with anti-Brp/NC82 (magenta) and anti-GFP (green). (*C*) Anti-En and anti-Poxn immunolabeling encompass *R65A11-LexA > mCD8::GFP* in AMMC (bracket). (*D*) Human *DACH1*-specific *cis*-regulatory sequence (CRE), *hs137,* targets LacZ expression to midbrain hindbrain boundary (MHB, arrow) in E11.5 mouse embryo (VISTA database). Data from ref. 53. (*E*) Sequence comparison of parts of *D. melanogaster dac R65A11*, mouse *mDach1*, and human *hDACH1 hs137* CREs. (*F*) Intragenic locations (black bar, asterisks) of *dac R65A11*, *hDACH1_hs137*, and corresponding mouse *mDach1* CRE sequences. (Scale bars: *A*, 40 μm; *B* and *C*, 50 μm.)

We also identified CREs for *dachshund* (*dac*) and the *Pax2* homolog *shaven* (*sv/dPax2*). *dac*-specific CRE *R65A11-Gal4* targeted *UAS-mCD8::GFP* expression to the DTB region, in derived lineages of the embryonic brain and to the AMMC in a pattern encompassed by DTB-specific *engrailed* and *Poxn* expression domains (Fig. 5 *A*–*C*). The regulatory element VT51937 (51) located in an intron of *sv/dPax2* targets Gal4 expression in a segment-specific pattern similar to endogenous *sv/dPax2*, including DTB expression domains (*SI Appendix*, Fig. S9 *A*–*F*). *VT51937-Gal4*–mediated genetic tracing also identified cells and projections in the AMMC (*SI Appendix*, Fig. S9*G*). Together, these data identify CREs of the DTB-AMMC character identity network in *Drosophila* that mediate the spatiotemporal expression patterns of genes that are homologous to genes involved in the formation and specification of the vertebrate MHB and derived midbrain-cerebellar circuitry (30–34, 50).

Given the correspondences between the *Drosophila* DTB and vertebrate MHB gene regulatory and character identity networks, we asked whether the CREs that control the expression of these genes might be conserved. To identify conserved cross-phylum CREs, we utilized DTB-AMMC–specific regulatory sequences and applied bioinformatics tools (52) including VISTA (53), MLAGAN (54), and EMBOSS MATCHER (55) to screen for corresponding CREs in mouse and human genomes (56). Stringent selection criteria (57) were applied to identify CRE sequences 1) that are linked to homologous genes in the different species; 2) with a minimum of 62% sequence identity over at least 55 base pairs (bp) and at least 1e^−1^ confidence level as the BLAST e-value; 3) that are not unannotated protein sequences; and 4) that are not repetitive elements. Additionally, BLAT (58) was applied for searching vertebrate genomes using the same selection criteria.

We first analyzed the DTB-AMMC–specific CRE of *sv/dPax2* (= VT51937 sequence) and identified noncoding CREs for mouse *Pax2* and human *PAX2* with extensive sequence similarities (*SI Appendix*, Fig. S9H and Supplementary Dataset S1), and comparable intragenic location (*SI Appendix*, Fig. S9*I*). To validate the significance of these sequences, we carried out BLAST/BLAT searches of the *sv/PAX2* conserved sequence against the *Drosophila melanogaster*, *Mus musculus*, and human genome sequences. These data revealed that, in *D. melanogaster*, only the *sv/dPax2*-related CRE of 255 bp with 1.6E−142 Blast e-value matches the cutoff criteria of >62% sequence identity over at least 55 bp with minimum 1^e-1^ confidence level as the BLAST e-value whereas all other identified sequences were of 18 bp or shorter length (*SI Appendix*, Supplementary Dataset S1). Of note, BLAT searches of the *sv/PAX2* conserved sequence against the mouse and human genome identified CREs only related to mouse *Pax2* and human *PAX2* and no other genomic sequence (*SI Appendix*, Supplementary Dataset S1). In addition, using the JASPAR algorithm (59), we identified potential transcription factor binding sites that match stretches of the MLAGAN-aligned *sv/PAX2* conserved CRE sequence (*SI Appendix*, Supplementary Dataset S1).

Following this strategy, we used DTB-AMMC–specific CRE sequences for *dachshund* and *engrailed/invected* and identified human CREs conserved among vertebrates that direct MHB-specific expression in mouse for the *dachshund* homologs *Dach1/DACH1* (Fig. 5 *D*–*F*) and for the *engrailed/invected* homologs *Engrailed2/EN2* (*SI Appendix*, Fig. S10). Further bioinformatics analysis identified core CRE sequences associated with *dac* and *DACH1*, *en/inv* and *EN2* and *sv* and *PAX2* in *Drosophilidae* and vertebrate genomes (*SI Appendix*, Supplementary Datasets S1–S3). As was the case for the *sv/PAX2* conserved CRE sequences, BLAST/BLAT searches against the *D. melanogaster*, *M. musculus*, and human genomes revealed for the *D. melanogaster* genome that only the *en/inv*-related CRE of 616 bp with 0.0 Blast e-value (*SI Appendix*, Supplementary Dataset S2) and *dac*-related CRE of 247 bp with 9.4E-138 Blast e-value (*SI Appendix*, Supplementary Dataset S3) match the cutoff criteria whereas all other identified sequences are of maximum 23 bp (for *en/inv*-related) or 30 bp (for *dac*-related) or shorter length. Also, BLAT searches of the *invected/engrailed-EN2* and *dachshund/DACH1* conserved sequences against the mouse and human genome only identified CREs for mouse *En2* and human *EN2*, and mouse *Dach1* and human *DACH1* respectively, whereas no other genomic sequences can be identified (*SI Appendix*, Supplementary Datasets S2 and S3). Furthermore, and again using the JASPAR algorithm, we identified potential transcription factor binding sites that match stretches of the MLAGAN-aligned *invected/engrailed-EN2* (*SI Appendix*, Supplementary Dataset S2) and *dachshund/DACH1* (*SI Appendix*, Supplementary Dataset S3) conserved CRE sequences, respectively. Together, these data suggest ancestral noncoding regulatory sequences and their function predate the radiation of insect-specific DTB and vertebrate-specific MHB circuits and morphologies.

## Discussion

We have identified gene regulatory and character identity networks that underlie the formation of the deutocerebral–tritocerebral boundary in *Drosophila.* Mutant analyses reveal that *otd* + *wg* and *msh* + *vnd*, acting along the AP and DV body axes, respectively, are required for the formation of the embryonic DTB, and that FGF8-like signaling is necessary for its developmental maintenance. Genetic tracing experiments, together with the analysis of CREs for *engrailed/invected*, *dachshund*, and *shaven/dPax2*, as well as behavioral analysis after synaptic inactivation, show that embryonic DTB lineages give rise to neural circuits in the AMMC/Wedge complex of the adult brain that mediate balance and motor coordination in *Drosophila*. Together, these findings establish a ground pattern of DTB formation and derived circuit function in *Drosophila* that corresponds to the ground pattern and gene regulatory and character identity networks of the vertebrate MHB and derived midbrain–cerebellar circuits (*SI Appendix*, Fig. S11).

**Fig. 6.**
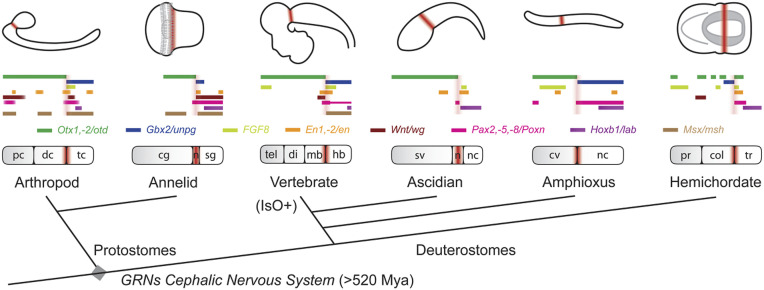
Phylogenetic comparison of DTB/MHB-related molecular signatures in nervous systems of extant Bilateria. Schematic diagram of homologous gene expression in central nervous system of arthropod *D. melanogaster*, annelid *Platynereis dumerilii*, vertebrate *M. musculus*, ascidian *C. intestinalis*, amphioxus *Branchiostoma floridae*, and ectodermal nervous system of hemichordate *Saccoglossus kowalevski.* Embryos and bar diagrams are oriented anterior to the left, dorsal up; brown coloring indicates boundary region. Multilevel correspondences of arthropod DTB and vertebrate MHB ground pattern organization suggest ancestral origin of gene regulatory networks (GRNs) for cephalic nervous systems >520 million years ago (Mya) that predate the radiation into protostomes and deuterostomes, and a suggested origin of the MHB-specific and proliferation related isthmic organizer (IsO) function in the vertebrate lineage. cg, cerebral ganglion; col, collar; cv, cerebral vesicle; dc, deutocerebrum; di, diencephalon; hb, hindbrain; mb, midbrain; n, neck; nc, nerve cord; pc, protocerebrum; pr, proboscis; sg, segmental ganglia; sv, sensory vesicle; tc, tritocerebrum; tel, telencephalon; tr, trunk.

In contrast to a previous gene expression study pointing toward the protocerebral/deutocerebral boundary (60), our findings based on gene regulation and function identify the *Drosophila* DTB as the boundary between the rostral brain and its genetically distinct caudal nervous system. These data imply that caudal domains of the arthropod deutocerebrum and its circuits in *Drosophila* correspond to the vertebrate MHB and its derived principle proprioceptive circuits (*SI Appendix*, Fig. S11). It must be emphasized here that these circuits are not ascribed to the cerebellum, the anlage of which forms as an asegmental volume within *Gbx2* and non-*Hox* expression domains of the developing MHB (30–33, 36, 61). Rather, our comparative analysis suggests that vestibular/balance-related circuits characterize the ancestral territory from which the DTB and MHB evolved, and that MHB-derived cerebellar circuitry is a vertebrate innovation. In support of this notion, FGF8 signaling in vertebrates acts as a secondary organizer in boundary development of the MHB and by promoting growth essential for the formation of the tectum and cerebellum (30–34, 36, 61). We did not observe FGF8-like organizer activity in flies but a role in the maintenance of the DTB boundary, suggesting an ancestral boundary-related function for FGF8 (61). A comparable phenotype has been described in ascidian larvae mutant for *Ffg8/17/18* (62). Normally expressed in the visceral ganglion, knockdown of *Fgf8/17/18* altered *Otx*, *Engrailed*, and *Pax2/5/8* gene expression in the anterior adjacent neck region of *Ciona*, essentially leading to a posterior expansion of rostral central nervous system (CNS) identity (62). These data suggest that, similar to the situation in the embryonic brain of *Drosophila*, FGF8 signaling in the tadpole larvae of *C. intestinalis* delineates the boundary between the rostral brain and the caudal nervous system. Moreover, the observed absence of extended proliferative activity in *Drosophila* (ref. 34 and this paper) and *Ciona* (62) suggests that growth-related organizer activity of FGF8 is a vertebrate innovation whereas the boundary region is defined by expression patterns of genes homologous to those observed at the DTB/MHB (Fig. 6). Indeed, despite comparable expression domains (11, 12, 63, 64), no phenotypic cerebellum is found in ascidians, nor in hemichordates and cephalochordates, none of which can be assumed as proxies for ancestral vertebrates, but all of which may represent highly derived and evolutionarily simplified crown species. However, ancestral circuits mediating vestibular and motor (balance) coordination, which are specified by genes and regulatory networks homologous to those described in this paper, are found in lampreys and hagfish, the persisting ancient lineages of early vertebrates (2), and maybe also in the chordate brain of *C. intestinalis* tadpole larvae (65).

The observed correspondences in circuit formation extend to behaviors they regulate. Synaptic inactivation of DTB-derived subcircuits of the AMMC/Wedge complex results in flies with impaired balance, defective action initiation and maintenance, and compromised sequences of motor actions. These AMMC circuits have been shown to mediate auditory and vestibular information processing and coordination (41, 42), suggesting that the DTB-derived AMMC/Wedge circuits integrate mechanosensory submodalities for motor homeostasis. These functions correspond to the activity of MHB-derived acoustic and vestibular receptor pathways in vertebrates (41, 44) which, when impaired in inherited disorders, affect both auditory and vestibular functions such as seen in ataxic patients (66). The observed correspondences therefore suggest that, similar to MHB-derived circuits, the DTB-derived AMMC/Wedge circuits in arthropods are required for sensorimotor integration, body posture, and motor coordination. These findings identify correspondences between ground patterns of the insect DTB and vertebrate MHB that extend beyond spatiotemporal expression patterns and functions of homologous genes, to neural circuits and behavior.

The present results identify CREs associated with highly conserved developmental control genes (67–70) regulating boundary formation between the rostral brain and the caudal nervous system in insects and vertebrates. Core elements of the identified CREs are highly conserved, as demonstrated by sequence identities across large phylogenetic distances and by the identification of potential transcription factor binding sites that are located within these conserved CREs. Although there is evidence that orthologous CREs can be completely divergent at the sequence level (62), our findings demonstrate that the identified conserved CREs are employed for the formation of circuits with comparable roles in neuronal processing. These data suggest the identified CREs are ancestral noncoding regulatory sequences with which insect-specific DTB and vertebrate-specific MHB circuits and morphologies evolved.

In conclusion, the corresponding ground patterns of insect DTB and vertebrate MHB suggest the early appearance in bilaterian evolution of a cephalic nervous system that evolved predictive motor homeostasis before the divergence of the protostome lineages and before the origin of deuterostomes. Based on the observed correspondences, we hypothesize that the retention across phyla of conserved regulatory mechanisms is necessary and sufficient for the formation and function of neural networks for adaptive behaviors common to all animals that possess a brain.

## Materials and Methods

### Fly Strains and Genetics.

The wild-type strain used was Oregon R. The following mutant alleles and characterization constructs were used to investigate expression and function: *P{en2.4-Gal4}e16E, UAS-mCD8::GFP*^*LL5*^ *and poxn*^*brain*^*-Gal4* as well as *UAS-mCD8::GFP, tub-FRT-CD2-FRT-Gal4, UAS-FLP/CyO GMR Dfd YFP* (71); *otd*^*JA101*^ (14); *P{lacZ}unpg*^*f85*^ (an *unpg-lacZ* reporter gene that expresses cytoplasmic β-galactosidase in the same pattern as endogenous *unpg*) (34); *P{lacZ}Pax2*^Δ*122*^ (a *Pax2-lacZ* reporter gene that expresses β-galactosidase in the same pattern as endogenous *Pax2*) (34); *P{3*′*lacZ}unpg*^*r37*^ (*unpg*-null allele with a *unpg-lacZ* reporter gene that expresses nuclear β-galactosidase in the same pattern as endogenous *unpg*) (34); *wg*^*CX4*^ and *wg-lacZ* (Bloomington); *msh*^Δ*68*^ (72)*; vnd*^*6*^ (73)*;* the deficiency *Df(2R)BSC25* that removes the FGF8-like 1 and FGF8-like 2 loci together with adjacent regions and *htl*^*AB42*^ (37) *UAS-FGF8-like 1* (37); and *ems2.6 (72.5)-Gal4* (73).

To generate *Poxn*^*brain*^*-Gal4* flies, the *Poxn* brain enhancer (74) was amplified by PCR from genomic DNA. The PCR product was subcloned into the *pPTGal* vector using XbaI and NotI sites, followed by sequencing; the genomic region 2R:11723830 to 11725559 was inserted into *pPTGal*. Primer sequences are as follows: forward, 5′-gct​cat​taa​tga​cca​tga​aa-3′; reverse, 5′-aag​cgg​ccg​cgt​taa​gta​acg​ctc​ggt​gg-3′. Transgenesis was performed by BestGene Inc.

For lineage tracing, the following strains were used: *w*^*1118*^ (control), *P{en2.4-Gal4}e16E, UAS-mCD8::GFP*^*LL5*^, or *poxn*^*brain*^*-Gal4* were crossed to *UAS-mCD8::GFP, tub-FRT-CD2-FRT-Gal4, UAS-FLP/CyO GMR Dfd YFP*. Offspring were raised at 18 °C to suppress random leaky FLP activity.

For behavioral experiments, we used *UAS-TNT-E* (47) crossed to AMMC-specific *Gal4* lines. Corresponding controls for *Gal4* driver and for UAS responder line were generated by backcrossing to *w*^*1118*^. All behavioral experiments were carried out in a temperature-controlled chamber at 25 °C.

### In Situ Hybridization, Immunocytochemistry, and Image Analysis.

For in situ hybridization experiments, digoxigenin (DIG)-labeled sense and antisense RNA probes were generated in vitro with a DIG labeling kit (Roche diagnostics) and hybridized to *Drosophila* whole-mount embryos, following standard procedures (75).

Whole-mount immunocytochemistry was performed as previously described (76, 77). Primary antibodies were rabbit anti-Otd (34), used 1:100; rabbit anti-Msh (72), used 1:500; rabbit anti-Vnd (73), used 1:200; rabbit anti-sv/dPax2 (34), used 1:50; monoclonal anti-Poxn antibodies (78), used 1:20; rabbit anti-horseradish peroxidase (HRP) (fluorescein isothiocyanate [FITC]-conjugated; Jackson Immunoresearch), used 1:50; mouse anti-En (Developmental Studies Hybridoma Bank [DSHB]), used 1:1; rabbit anti-β-Gal, used 1:200 (Milan Analytica); mouse anti-β-Gal (DSHB), used 1:100; rabbit anti-Lab (79), used 1:50; rat anti-Ems (80), used 1:2,000; mouse anti-Bruchpilot (nc82; DSHB), used 1:20; mouse anti-Synapsin (3C11; DSHB), used 1:50; rabbit anti-GFP (Invitrogen), used 1:500; rabbit anti-GABA (Sigma-Aldrich), used 1:1,000. Secondary antibodies were Alexa-568–conjugated goat anti-mouse, Alexa-568–conjugated goat anti-rabbit, Alexa-568–conjugated goat anti-rat, Alexa-488–conjugated goat anti-mouse, Alexa-488–conjugated goat anti-rabbit, and Alexa-488–conjugated goat anti-rat (Molecular probes), all used 1:150. Embryos, larval CNS, and adult brains were mounted in Vectashield H-1000 (Vector).

Fluorescence samples were scanned and recorded with a Leica TCS SP5 confocal microscope. Z-projections were created and analyzed using FIJI. Images were processed using Adobe Photoshop, and figures were constructed in Adobe Illustrator.

### SING Assay.

Groups of 10 flies with shortened wings of the same age, sex, and genotype were placed in a vertical column (19 cm long, 2 cm diameter). The wings were clipped under sedation (with CO_2_) at least 24 h prior to testing. They were suddenly startled by gently tapping them down, to which *Drosophila* responds by climbing up. After 10 s, it was counted how many flies were above the 2 cm mark, and the trial was repeated 15 times for each tube and the average was calculated. For each genotype, 10 groups of females and 10 groups of males were tested. Flies were reared at 25 °C and were maintained under a 12 h light/dark cycle. Flies with an average age of 5 d were tested at room temperature, under the same light conditions. All assays were performed at the same time of day.

### Motor Behavior Analysis.

Control and experimental flies were reared at 18 °C, and adult mated females up to 5 d posteclosion were transferred to 25 °C for behavioral analyses. Mechanical stimuli trains consisted of five pulses of 200 ms each, spaced by 800 ms. Motor behavior parameters were determined as previously described (38, 81, 82).

### Bioinformatics Analyses and Identification of CREs.

The Janelia Gal4 collection (https://flweb.janelia.org/cgi-bin/flew.cgi) (49) was screened for AMMC-specific GFP expression patterns. All hits were cross-checked to be verified/excluded from the Janelia/Bloomington list (see https://bdsc.indiana.edu/stocks/gal4/gal4_janelia_info.html). For each hit, annotated left and right primers were used to BLAST the *Drosophila* genome annotated at the *Ensembl* genome browser (http://www.ensembl.org/index.html) to determine the position and sequence within genome version BDGP6, or, where known, the sequence was extracted from the respective gene map annotation in JBrowse (flybase.org/). The resulting sequence was compared against available Vienna Tiles (VT) enhancer sequences and their annotated expression pattern determined for DTB expression patterns (https://enhancers.starklab.org) (51). *Drosophila*-specific, noncoding regulatory sequences were then used for BLAT searches (58) to screen the mouse (GRCm38.p5) and human (GRCh38.p7) genome annotated at Ensemble (https://www.ensembl.org/index.html) to identify any potential corresponding sequences. Any matching sequences were scrutinized for further analysis on the basis of criteria that have been used previously to identify transphyletic *cis*-regulatory DNA sequences (57). These criteria were as follows: 1) The sequences are linked to the same homologous genes in the different species; 2) there is a minimum of 62% sequence identity over at least 55 bp with minimum 1e^−1^ confidence level as the BLAST e-value; 3) the CREs are not unannotated protein sequences; and 4) the CREs are not repetitive elements.

Because the algorithm used by BLAST/BLAT does not support the first stated threshold criterion, our search was restricted to and thus focused on the genomic regions encompassing homologous genes. In the case of more than one homolog (e.g., *DACH1* and *DACH2*), both homologs were included in the search. In the case of *EN2*, we additionally included the intergenic region between the two *Drosophila* homologs, *invected* and *engrailed*.

The resulting sequences were then used for refined comparisons using pair-wise and multiple sequence alignment algorithms including EMBOSS Matcher and t-coffee (https://www.ebi.ac.uk/services). To carry out sequence alignments, which automatically indicated whether any CNS regulatory elements might be covered by the input sequences, we used the MLAGAN algorithm (http://genome.lbl.gov/vista/lagan/submit.shtml). Detected CNS CREs were then further scrutinized using the VISTA enhancer browser (https://enhancer.lbl.gov/frnt_page_n.shtml), which provides human and mouse regulatory sequences and their expression pattern at embryonic stage E11.5 in transgenic mouse embryos expressing LacZ under the control of the respective regulatory sequence (53, 56). The relevant images of LacZ expression were extracted and reproduced with permission by Len Pennacchio, Lawrence Berkeley National Laboratory. Identified MHB-specific regulatory sequences were utilized to perform multiple sequence alignment with the respective DTB→AMMC-specific regulatory elements; any matches were reconfirmed and quantified using the EMBOSS Matcher and CLUSTAL Omega (https://www.ebi.ac.uk/Tools/msa/clustalo/) algorithms. Potential transcription factor binding sites (TFBS) that match stretches of the MLAGAN-aligned conserved CRE sequences were identified using the JASPAR algorithm (jaspar.genereg.net/) (59).

Maximum likelihood phylogenetic trees were inferred by the Genome-to-Genome-Distance Calculator (GGDC) available at the Leibniz Institute German Collection of Microorganisms and Cell Cultures (https://ggdc.dsmz.de/phylogeny-service.php) from a MUSCLE multiple sequence alignment with Randomized Axelerated Maximum Likelihood (83).

### Statistical Analysis.

Each dataset was tested for normality using the Anderson–Darling test with α = 0.05. If every dataset under comparison was normal and the variances were similar (Hartley’s fmax was calculated in each case and used as a cutoff for variance ratio), then a one-way ANOVA test was used to determine whether any differences existed between groups. If significance was found for ANOVA with α = 0.05, then pair-wise comparisons were made using a post hoc Tukey–Kramer test, again with α = 0.05. If any of the datasets were found not to be normally distributed, then a Kruskal–Wallis test was used to determine any overall differences between the groups with α = 0.05. If significance was achieved, a post hoc pairwise Mann–Whitney *U* test with Dunn–Sidak correction was used to compare groups with α = 0.05. For each test group, two controls were used corresponding to the two genetic elements that were altered in the group under analysis. For example, *R11A07 > TNT* would be tested against TNT control and *R11A07 > w1118*. For a result to be considered significant, the experimental group had to be significantly different from both controls and the controls not be significantly different from one another. All calculations were performed using MATLAB.

## Supplementary Material

Supplementary File

Supplementary File

Supplementary File

## Data Availability

All data of this study are included in the manuscript and *SI Appendix*.

## References

[1] Robertson B.., The lamprey blueprint of the mammalian nervous system. Prog. Brain Res. 212, 337–349 (2014). 25194205 10.1016/B978-0-444-63488-7.00016-1

[2] Sugahara F.., Evidence from cyclostomes for complex regionalization of the ancestral vertebrate brain. Nature 531, 97–100 (2016). 26878236 10.1038/nature16518

[3] Brown M., Keynes R., Lumsden A., The Developing Brain, (Oxford Univ. Press, 2001).

[4] Strausfeld N. J., Arthropod Brains: Evolution, Functional Elegance and Historical Significance, (Belknap Press of Harvard Univ. Press, 2012).

[5] Cong P., Ma X., Hou X., Edgecombe G. D., Strausfeld N. J., Brain structure resolves the segmental affinity of anomalocaridid appendages. Nature 513, 538–542 (2014). 25043032 10.1038/nature13486

[6] Ghysen A., The origin and evolution of the nervous system. Int. J. Dev. Biol. 47, 555–562 (2003). 14756331

[7] Hirth F., Reichert H., “Basic nervous system types: One or many?” in Evolution of Nervous Systems, Bullock T. H., Ed. (Elsevier, 2005), Vol. I, pp. 55–72.

[8] Hirth F., On the origin and evolution of the tripartite brain. Brain Behav. Evol. 76, 3–10 (2010). 20926853 10.1159/000320218

[9] Strausfeld N. J., Hirth F., Deep homology of arthropod central complex and vertebrate basal ganglia. Science 340, 157–161 (2013). 23580521 10.1126/science.1231828

[10] Wolff G. H., Strausfeld N. J., Genealogical correspondence of a forebrain centre implies an executive brain in the protostome-deuterostome bilaterian ancestor. Philos. Trans. R. Soc. Lond. B Biol. Sci. 371, 20150055 (2016). 26598732 10.1098/rstb.2015.0055 PMC4685588

[11] Holland N. D., Early central nervous system evolution: An era of skin brains? Nat. Rev. Neurosci. 4, 617–627 (2003). 12894237 10.1038/nrn1175

[12] Holland L. Z.., Evolution of bilaterian central nervous systems: A single origin? Evodevo 4, 27 (2013). 24098981 10.1186/2041-9139-4-27 PMC3856589

[13] Hirth F., Reichert H., Conserved genetic programs in insect and mammalian brain development. BioEssays 21, 677–684 (1999). 10440864 10.1002/(SICI)1521-1878(199908)21:8<677::AID-BIES7>3.0.CO;2-8

[14] Hirth F.., Developmental defects in brain segmentation caused by mutations of the homeobox genes *orthodenticle* and *empty spiracles* in *Drosophila*. Neuron 15, 769–778 (1995). 7576627 10.1016/0896-6273(95)90169-8

[15] Simeone A., Otx1 and Otx2 in the development and evolution of the mammalian brain. EMBO J. 17, 6790–6798 (1998). 9843484 10.1093/emboj/17.23.6790 PMC1171026

[16] Nagao T.., Developmental rescue of Drosophila cephalic defects by the human Otx genes. Proc. Natl. Acad. Sci. U.S.A. 95, 3737–3742 (1998). 9520436 10.1073/pnas.95.7.3737 PMC19906

[17] Leuzinger S.., Equivalence of the fly orthodenticle gene and the human OTX genes in embryonic brain development of Drosophila. Development 125, 1703–1710 (1998). 9521908 10.1242/dev.125.9.1703

[18] Acampora D.., Murine Otx1 and Drosophila otd genes share conserved genetic functions required in invertebrate and vertebrate brain development. Development 125, 1691–1702 (1998). 9521907 10.1242/dev.125.9.1691

[19] Acampora D.., OTD/OTX2 functional equivalence depends on 5′ and 3′ UTR-mediated control of Otx2 mRNA for nucleo-cytoplasmic export and epiblast-restricted translation. Development 128, 4801–4813 (2001). 11731460 10.1242/dev.128.23.4801

[20] Hanks M. C.., *Drosophila* engrailed can substitute for mouse Engrailed1 function in mid-hindbrain, but not limb development. Development 125, 4521–4530 (1998). 9778510 10.1242/dev.125.22.4521

[21] Strausfeld N. J., Hirth F., Homology versus convergence in resolving transphyletic correspondences of brain organization. Brain Behav. Evol. 82, 215–219 (2013). 24296550 10.1159/000356102

[22] Fiore V. G., Dolan R. J., Strausfeld N. J., Hirth F., Evolutionarily conserved mechanisms for the selection and maintenance of behavioural activity. Philos. Trans. R. Soc. Lond. B Biol. Sci. 370, 20150053 (2015). 26554043 10.1098/rstb.2015.0053 PMC4650127

[23] Fiore V. G.., Changing pattern in the basal ganglia: Motor switching under reduced dopaminergic drive. Sci. Rep. 6, 23327 (2016). 27004463 10.1038/srep23327 PMC4804216

[24] Strausfeld N. J., Hirth F., Introduction to “Homology and convergence in nervous system evolution”. Philos. Trans. R. Soc. Lond. B Biol. Sci. 371, 20150034 (2016). 26598720 10.1098/rstb.2015.0034 PMC4685576

[25] Strausfeld N. J., Ma X., Edgecombe G. D., Fossils and the evolution of the arthropod brain. Curr. Biol. 26, R989–R1000 (2016). 27780074 10.1016/j.cub.2016.09.012

[26] Erwin D. H., Davidson E. H., The evolution of hierarchical gene regulatory networks. Nat. Rev. Genet. 10, 141–148 (2009). 19139764 10.1038/nrg2499

[27] Wagner G. P., The developmental genetics of homology. Nat. Rev. Genet. 8, 473–479 (2007). 17486120 10.1038/nrg2099

[28] Wagner G. P., Homology, Genes and Evolutionary Innovation, (Princeton Univ. Press, 2014).

[29] Arendt D.., The origin and evolution of cell types. Nat. Rev. Genet. 17, 744–757 (2016). 27818507 10.1038/nrg.2016.127

[30] Rhinn M., Brand M., The midbrain–hindbrain boundary organizer. Curr. Opin. Neurobiol. 11, 34–42 (2001). 11179870 10.1016/s0959-4388(00)00171-9

[31] Wurst W., Bally-Cuif L., Neural plate patterning: Upstream and downstream of the isthmic organizer. Nat. Rev. Neurosci. 2, 99–108 (2001). 11253000 10.1038/35053516

[32] Dworkin S., Jane S. M., Novel mechanisms that pattern and shape the midbrain-hindbrain boundary. Cell. Mol. Life Sci. 70, 3365–3374 (2013). 23307071 10.1007/s00018-012-1240-x PMC11113640

[33] Harada H., Sato T., Nakamura H., Fgf8 signaling for development of the midbrain and hindbrain. Dev. Growth Differ. 58, 437–445 (2016). 27273073 10.1111/dgd.12293

[34] Hirth F.., An urbilaterian origin of the tripartite brain: Developmental genetic insights from *Drosophila*. Development 130, 2365–2373 (2003). 12702651 10.1242/dev.00438

[35] Levin M.., The mid-developmental transition and the evolution of animal body plans. Nature 531, 637–641 (2016). 26886793 10.1038/nature16994 PMC4817236

[36] Sato T., Joyner A. L., The duration of Fgf8 isthmic organizer expression is key to patterning different tectal-isthmo-cerebellum structures. Development 136, 3617–3626 (2009). 19793884 10.1242/dev.041210 PMC2761110

[37] Gryzik T., Müller H. A., FGF8-like1 and FGF8-like2 encode putative ligands of the FGF receptor Htl and are required for mesoderm migration in the *Drosophila* gastrula. Curr. Biol. 14, 659–667 (2004). 15084280 10.1016/j.cub.2004.03.058

[38] Stathopoulos A., Tam B., Ronshaugen M., Frasch M., Levine M., Pyramus and thisbe: FGF genes that pattern the mesoderm of Drosophila embryos. Genes Dev. 18, 687–699 (2004). 15075295 10.1101/gad.1166404 PMC387243

[39] Shaw R. E.., *In vivo* expansion of functionally integrated GABAergic interneurons by targeted increase in neural progenitors. EMBO J. 37, 98163 (2018). 10.15252/embj.201798163 PMC6028031 29728368

[40] Bridi J. C., Ludlow Z. N., Hirth F., Lineage-specific determination of ring neuron circuitry in the central complex of *Drosophila*. Biol. Open 8, bio045062 (2019). 31285267 10.1242/bio.045062 PMC6679397

[41] Kamikouchi A.., The neural basis of *Drosophila* gravity-sensing and hearing. Nature 458, 165–171 (2009). 19279630 10.1038/nature07810

[42] Vaughan A. G., Zhou C., Manoli D. S., Baker B. S., Neural pathways for the detection and discrimination of conspecific song in D. melanogaster. Curr. Biol. 24, 1039–1049 (2014). 24794294 10.1016/j.cub.2014.03.048

[43] Matsuo E.., Organization of projection neurons and local neurons of the primary auditory center in the fruit fly *Drosophila melanogaster*. J. Comp. Neurol. 524, 1099–1164 (2016). 26762251 10.1002/cne.23955

[44] Tsubouchi A.., Topological and modality-specific representation of somatosensory information in the fly brain. Science 358, 615–623 (2017). 29097543 10.1126/science.aan4428

[45] Zinyk D. L., Mercer E. H., Harris E., Anderson D. J., Joyner A. L., Fate mapping of the mouse midbrain-hindbrain constriction using a site-specific recombination system. Curr. Biol. 8, 665–668 (1998). 9635195 10.1016/s0960-9822(98)70255-6

[46] Baumann O.., Consensus paper: The role of the cerebellum in perceptual processes. Cerebellum 14, 197–220 (2015). 25479821 10.1007/s12311-014-0627-7 PMC4346664

[47] Sweeney S. T., Broadie K., Keane J., Niemann H., O’Kane C. J., Targeted expression of tetanus toxin light chain in Drosophila specifically eliminates synaptic transmission and causes behavioral defects. Neuron 14, 341–351 (1995). 7857643 10.1016/0896-6273(95)90290-2

[48] White K. E., Humphrey D. M., Hirth F., The dopaminergic system in the aging brain of *Drosophila*. Front. Neurosci. 4, 205 (2010). 21165178 10.3389/fnins.2010.00205 PMC3002484

[49] Jenett A.., A GAL4-driver line resource for *Drosophila* neurobiology. Cell Rep. 2, 991–1001 (2012). 23063364 10.1016/j.celrep.2012.09.011 PMC3515021

[50] Oberdick J., Baader S. L., Schilling K., From zebra stripes to postal zones: Deciphering patterns of gene expression in the cerebellum. Trends Neurosci. 21, 383–390 (1998). 9735946 10.1016/s0166-2236(98)01325-3

[51] Kvon E. Z.., Genome-scale functional characterization of *Drosophila* developmental enhancers in vivo. Nature 512, 91–95 (2014). 24896182 10.1038/nature13395

[52] Madeira F.., The EMBL-EBI search and sequence analysis tools APIs in 2019. Nucleic Acids Res. 47, W636–W641 (2019). 30976793 10.1093/nar/gkz268 PMC6602479

[53] Visel A., Minovitsky S., Dubchak I., Pennacchio L. A., VISTA Enhancer Browser–a database of tissue-specific human enhancers. Nucleic Acids Res. 35, D88–D92 (2007). 17130149 10.1093/nar/gkl822 PMC1716724

[54] Woolfe A.., Highly conserved non-coding sequences are associated with vertebrate development. PLoS Biol. 3, e7 (2005). 15630479 10.1371/journal.pbio.0030007 PMC526512

[55] Waterman M. S., Eggert M., A new algorithm for best subsequence alignments with application to tRNA-rRNA comparisons. J. Mol. Biol. 197, 723–728 (1987). 2448477 10.1016/0022-2836(87)90478-5

[56] Pennacchio L. A.., In vivo enhancer analysis of human conserved non-coding sequences. Nature 444, 499–502 (2006). 17086198 10.1038/nature05295

[57] Royo J. L.., Transphyletic conservation of developmental regulatory state in animal evolution. Proc. Natl. Acad. Sci. U.S.A. 108, 14186–14191 (2011). 21844364 10.1073/pnas.1109037108 PMC3161536

[58] Kent W. J., BLAT–the BLAST-like alignment tool. Genome Res. 12, 656–664 (2002). 11932250 10.1101/gr.229202 PMC187518

[59] Fornes O.., JASPAR 2020: Update of the open-access database of transcription factor binding profiles. Nucleic Acids Res. 48, D87–D92 (2020). 31701148 10.1093/nar/gkz1001 PMC7145627

[60] Urbach R., A procephalic territory in *Drosophila* exhibiting similarities and dissimilarities compared to the vertebrate midbrain/hindbrain boundary region. Neural Dev. 2, 23 (2007). 17983473 10.1186/1749-8104-2-23 PMC2206033

[61] Butts T., Green M. J., Wingate R. J., Development of the cerebellum: Simple steps to make a “little brain”. Development 141, 4031–4041 (2014). 25336734 10.1242/dev.106559

[62] Imai K. S., Stolfi A., Levine M., Satou Y., Gene regulatory networks underlying the compartmentalization of the *Ciona* central nervous system. Development 136, 285–293 (2009). 19088089 10.1242/dev.026419

[63] Pani A. M.., Ancient deuterostome origins of vertebrate brain signalling centres. Nature 483, 289–294 (2012). 22422262 10.1038/nature10838 PMC3719855

[64] Cao C.., Comprehensive single-cell transcriptome lineages of a proto-vertebrate. Nature 571, 349–354 (2019). 31292549 10.1038/s41586-019-1385-y PMC6978789

[65] Ryan K., Lu Z., Meinertzhagen I. A., The CNS connectome of a tadpole larva of *Ciona intestinalis* (L.) highlights sidedness in the brain of a chordate sibling. eLife 5, e16962 (2016). 27921996 10.7554/eLife.16962 PMC5140270

[66] Barsottini O. G., Pedroso J. L., Martins C. R. Jr., França M. C. Jr., Albernaz P. M., Deafness and vestibulopathy in cerebellar diseases: A practical approach. Cerebellum 18, 1011–1016 (2019). 31154624 10.1007/s12311-019-01042-4

[67] Wray G. A., The evolutionary significance of cis-regulatory mutations. Nat. Rev. Genet. 8, 206–216 (2007). 17304246 10.1038/nrg2063

[68] Carroll S. B., Evo-devo and an expanding evolutionary synthesis: A genetic theory of morphological evolution. Cell 134, 25–36 (2008). 18614008 10.1016/j.cell.2008.06.030

[69] Shubin N., Tabin C., Carroll S., Deep homology and the origins of evolutionary novelty. Nature 457, 818–823 (2009). 19212399 10.1038/nature07891

[70] Alberch P., From genes to phenotype: Dynamical systems and evolvability. Genetica 84, 5–11 (1991). 1874440 10.1007/BF00123979

[71] Roy B.., Metamorphosis of an identified serotonergic neuron in the *Drosophila* olfactory system. Neural Dev. 2, 20 (2007). 17958902 10.1186/1749-8104-2-20 PMC2129096

[72] Sprecher S. G., Hirth F., Expression and function of the columnar patterning gene *msh* in late embryonic brain development of *Drosophila*. Dev. Dyn. 235, 2920–2929 (2006). 16929521 10.1002/dvdy.20936

[73] Sprecher S. G.., The columnar gene vnd is required for tritocerebral neuromere formation during embryonic brain development of *Drosophila*. Development 133, 4331–4339 (2006). 17038518 10.1242/dev.02606

[74] Boll W., Noll M., The Drosophila Pox neuro gene: Control of male courtship behavior and fertility as revealed by a complete dissection of all enhancers. Development 129, 5667–5681 (2002). 12421707 10.1242/dev.00157

[75] Tautz D., Pfeifle C., A non-radioactive in situ hybridization method for the localization of specific RNAs in Drosophila embryos reveals translational control of the segmentation gene hunchback. Chromosoma 98, 81–85 (1989). 2476281 10.1007/BF00291041

[76] Hirth F., Hartmann B., Reichert H., Homeotic gene action in embryonic brain development of Drosophila. Development 125, 1579–1589 (1998). 9521896 10.1242/dev.125.9.1579

[77] Bello B. C., Hirth F., Gould A. P., A pulse of the Drosophila Hox protein Abdominal-A schedules the end of neural proliferation via neuroblast apoptosis. Neuron 37, 209–219 (2003). 12546817 10.1016/s0896-6273(02)01181-9

[78] Hassanzadeh GH G.., Isolation and characterization of single-chain Fv genes encoding antibodies specific for *Drosophila* Poxn protein. FEBS Lett. 437, 75–80 (1998). 9804175 10.1016/s0014-5793(98)01204-6

[79] Sprecher S. G.., Hox gene cross-regulatory interactions in the embryonic brain of Drosophila. Mech. Dev. 121, 527–536 (2004). 15172684 10.1016/j.mod.2004.04.009

[80] Walldorf U., Gehring W. J., Empty spiracles, a gap gene containing a homeobox involved in Drosophila head development. EMBO J. 11, 2247–2259 (1992). 1376248 10.1002/j.1460-2075.1992.tb05284.x PMC556692

[81] Faville R., Kottler B., Goodhill G. J., Shaw P. J., van Swinderen B., How deeply does your mutant sleep? Probing arousal to better understand sleep defects in *Drosophila*. Sci. Rep. 5, 8454 (2015). 25677943 10.1038/srep08454 PMC4326961

[82] Mazaud D.., Transcriptional regulation of the Glutamate/GABA/Glutamine cycle in adult glia controls motor activity and seizures in *Drosophila*. J. Neurosci. 39, 5269–5283 (2019). 31064860 10.1523/JNEUROSCI.1833-18.2019 PMC6607755

[83] Meier-Kolthoff J. P.., Complete genome sequence of DSM 30083(T), the type strain (U5/41(T)) of Escherichia coli, and a proposal for delineating subspecies in microbial taxonomy. Stand. Genomic Sci. 9, 2 (2014). 25780495 10.1186/1944-3277-9-2 PMC4334874

