# Supplementary material for: Ancestral regulatory mechanisms specify conserved midbrain circuitry in arthropods and vertebrates
Source: Proc Natl Acad Sci U S A. 2020 Aug 3;117(32):19544–55. doi: 10.1073/pnas.1918797117 (PMC7431035; doi:10.1073/pnas.1918797117)
Supplement: Supplementary File [file pnas.1918797117.sapp.pdf]

PNAS MS# 2019-18797

SUPPLEMENTARY INFORMATION

Ancestral regulatory mechanisms specify conserved midbrain circuitry in arthropods and vertebrates

Jessika C. Bridi[1,†], Zoe N. Ludlow[1,†], Benjamin Kottler[1], Beate Hartmann[2], Lies Vanden Broeck[3], Jonah Dearlove[1], Markus Göker[4], Nicholas J. Strausfeld[5], Patrick Callaerts[3] and Frank Hirth[1,*]

[†]These authors contributed equally to this work.

Lead-Correspondence to: Frank.Hirth@kcl.ac.uk
Co-correspondence: flybrain@arizona.edu

This pdf file includes:

Figs. S1 to S11
Tables S1 to S2
Captions for movies S1 to S2
Captions for databases S1 to S3
References for SI reference citations
Data Set S1 to S3

Other Supplementary Information for this manuscript includes the following:

Movies S1 and S2

www.pnas.org/cgi/doi/10.1073/pnas.1918797117

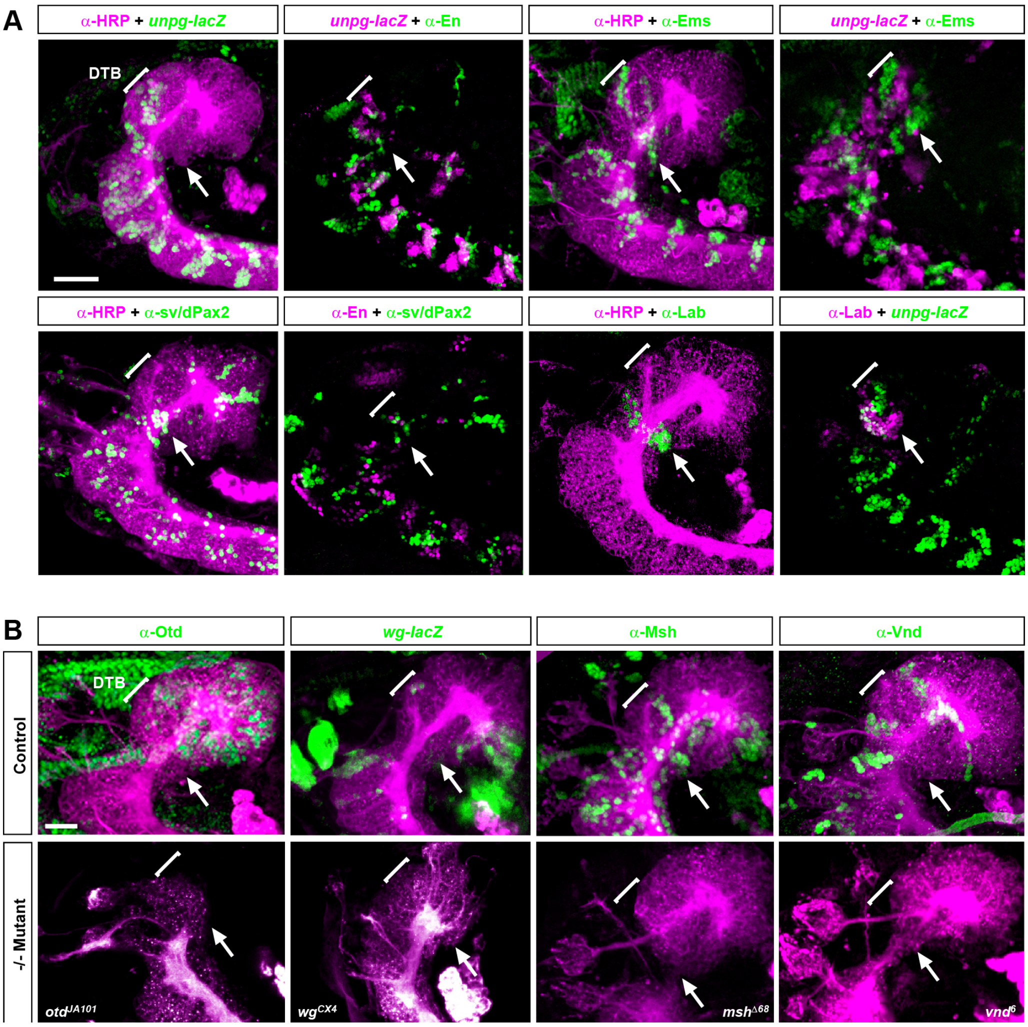

Fig. S1. Expression and function of genes that characterize the DTB region in the embryonic brain of *Drosophila.* (A) confocal images of stage 13-15 embryonic brains, lateral views; brackets indicate position of the deutocerebral-tritocerebral boundary (DTB) region, arrows highlight DTB-specific expression domains. From left to right, upper row: anterior-most expression of *Gbx* homologue *unplugged* (*unpg*) demarcates the DTB. *engrailed* (*en*) expression at the DTB coincides with *unpg* expression. Anterior-most expression of *empty spiracles* (*ems*) coincides with the DTB. Anterior-most expression domains of *unpg* and *ems* within the DTB. Lower row: prominent *sv/dPax2* expression domain within the DTB. DTB-specific expression of *sv/dPax2* coincides with *engrailed* expression at the DTB. The Hox1 ortholog *labial* (*lab*) demarcates the posterior part of the DTB and partially overlaps with the anterior *unpg* expression domain. (B) expression and function of genes involved in anterior-posterior (AP) and dorso-ventral (DV) axis specification. The *Otx* homologue *orthodenticle* (*otd*) posterior-most expression in the forebrain demarcates the DTB. HRP-labelled embryonic brain of *otd* null mutant reveals patterning defects, deleting all structures anterior to the tritocerebrum, including the DTB. Expression of *wingless* (*wg-lacZ*) in the anterior-most part of the embryonic brain and in a pattern coinciding with the DTB. HRP-labelled embryonic brain of *wg* null mutant reveals brain patterning severely affecting the DTB. Expression of *muscle-specific homeobox* (*msh*) in the anterior embryonic brain, including prominent DTB-specific expression pattern. HRP-labelled embryonic brain of *msh* null mutant reveals DTB patterning defects. Expression of *ventral nervous system defective* (*vnd*) in the anterior embryonic brain, including prominent DTB-specific expression pattern. HRP-labelled embryonic brain of *vnd* null mutant reveals severe DTB patterning defects. Scale bars: 20μm (A), 10μm (B).

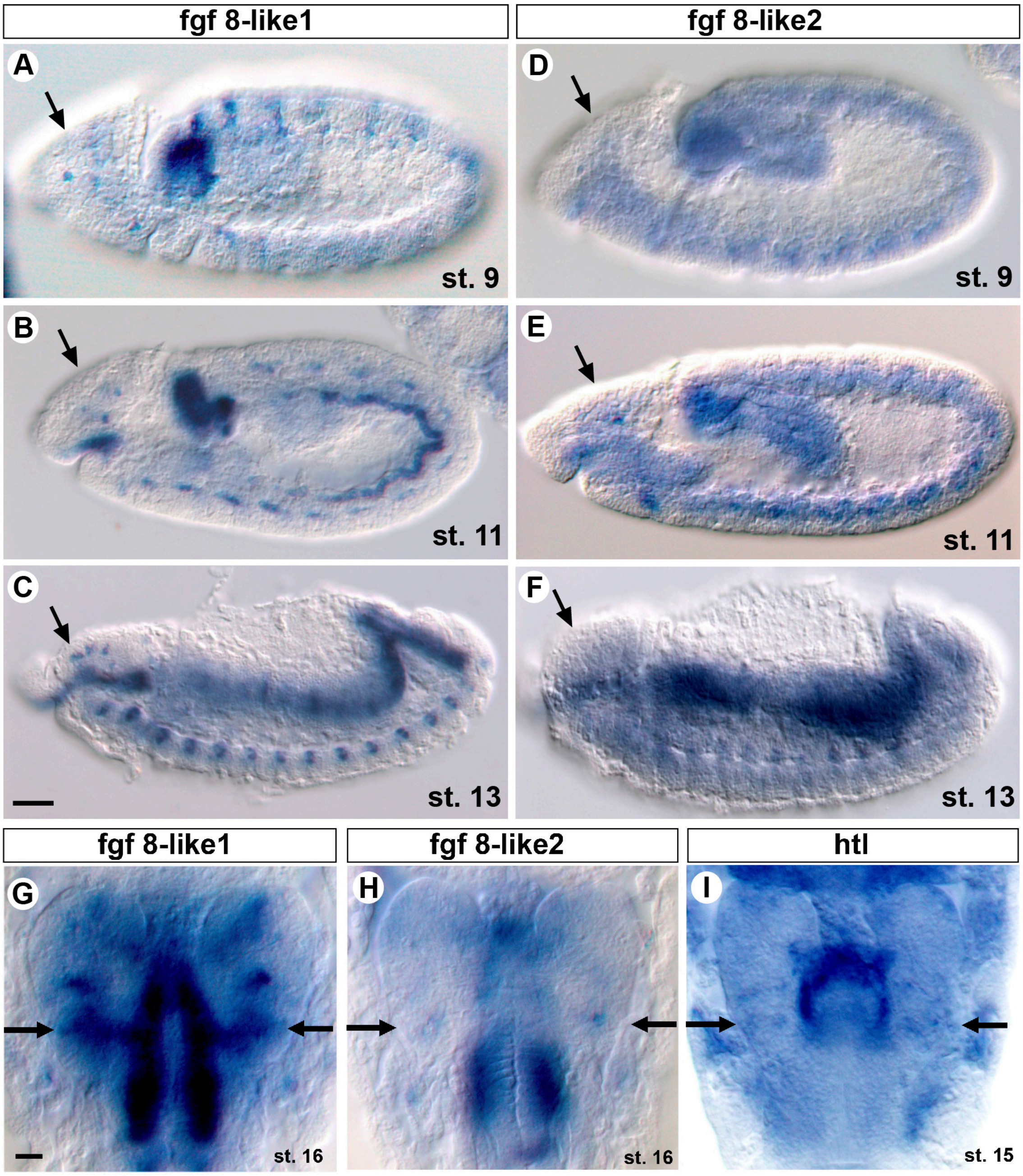

Fig. S2. Expression of *FGF8-like1* and *FGF8-like2* in the embryonic nervous system and brain of *Drosophila.* Whole-mount in situ hybridization of transcripts of (A-C) *FGF8-like1 (thisbe, ths)* and, (D-F) *FGF8-like2 (pyramus, pyr)* in wildtype embryos at indicated stages. Lateral views, anterior is to the left; arrows demarcate the primordium of the deutocerebral-tritocerebral boundary (DTB) region. (G, H) Anterior brains of stage 16 embryos, dorsal views; arrows demarcate *FGF8-like1* and *FGF8-like2* expression in the DTB. (I) In situ hybridization of FGF8-like receptor *heartless* (*htl*) transcripts in stage 16 anterior brain of wildtype embryo; arrow demarcates *htl* expression in the DTB. Scale bars: 20μm (C), 10μm (G).

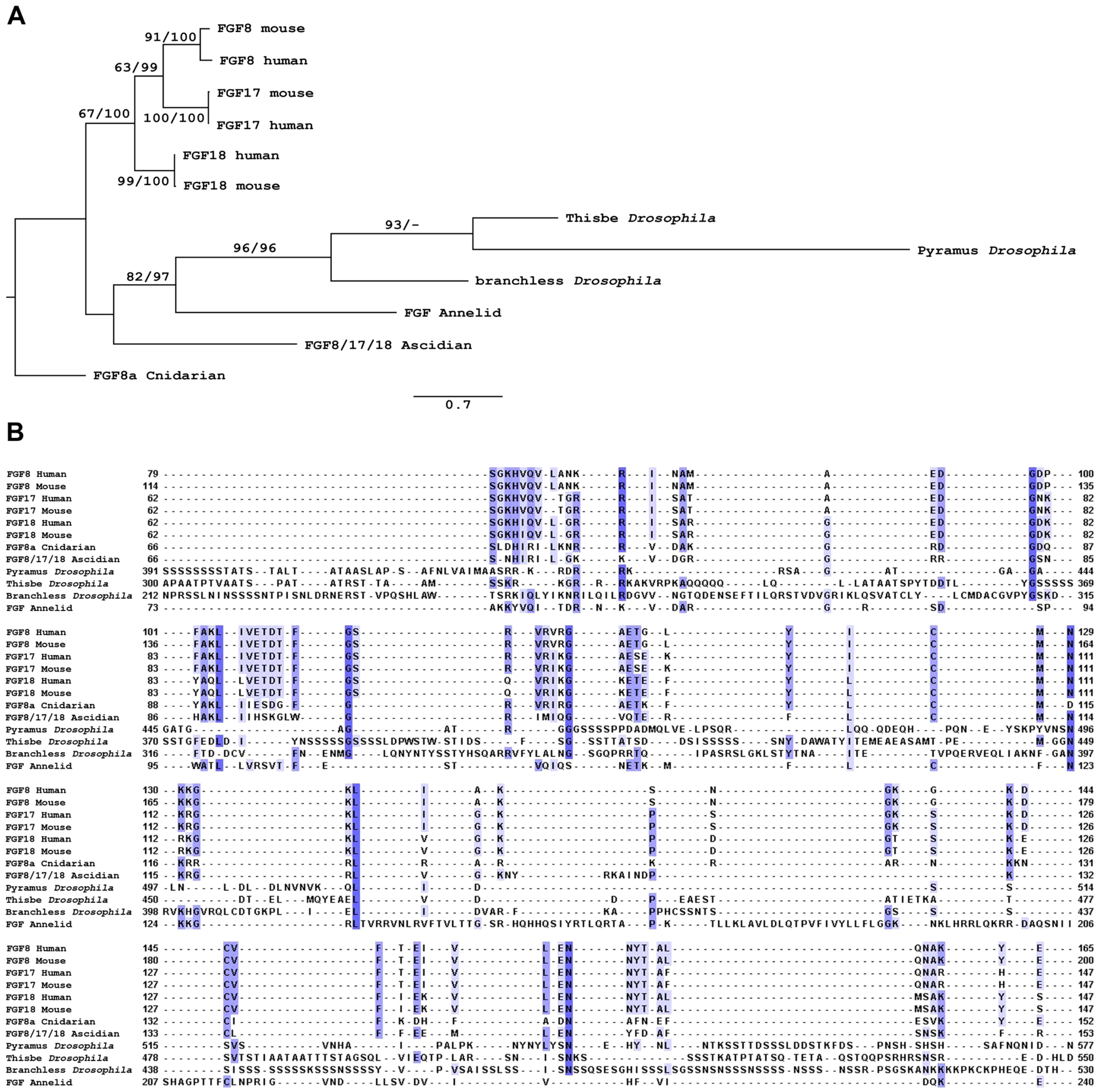

Fig. S3. Phylogenetic comparison of *Drosophila FGF8-like1* and *FGF8-like2* proteins. (A) Phylogenetic tree, and (B) partial sequence comparison of Fibroblast growth factor 8 family homologs of human FGF8, FGF17 and FGF18; mouse FGF8, FGF17 and FGF18; Cnidarian *Nematostella vectensis* FGF8a; Ascidian *Ciona intestinalis* FGF8/17/18; *Drosophila melanogaster* Pyramus, Thisbe and Branchless; and Annelid *Capitella teleta* FGF. Maximum likelihood (ML) phylogenetic tree in (A) was inferred by the GGDC web server (https://ggdc.dsmz.de/phylogeny-service.php) from a MUSCLE multiple sequence alignment with Randomized Axelerated Maximum Likelihood (RAxML); support values on the branches are from ML bootstrapping with RAxML (left) and from maximum parsimony (MP) bootstrapping with Tree analysis using New Technology (TNT) (right) when larger than 60%. Sequence comparison in (B) was generated using the Kalign neighbour joining algorithm available at the European Bioinformatics Institute (www.ebi.ac.uk/Tools/msa/kalign/). Protein sequences were derived from UniProt, accession numbers P55075, hFGF8; P37237, mFGF8; O60258, hFGF17; P63075, mFGF17; O76093, hFGF18; O89101, mFGF18; A7YAZ8, NEMVE FGF8a; Q8I6J5, CIOIN FGF 8/17/18; B9ZW35, DROME Pyramus; Q6Q7I9, DROME Thisbe; Q9VDT9, DROME branchless; X2AP22, CAPTE FGF.

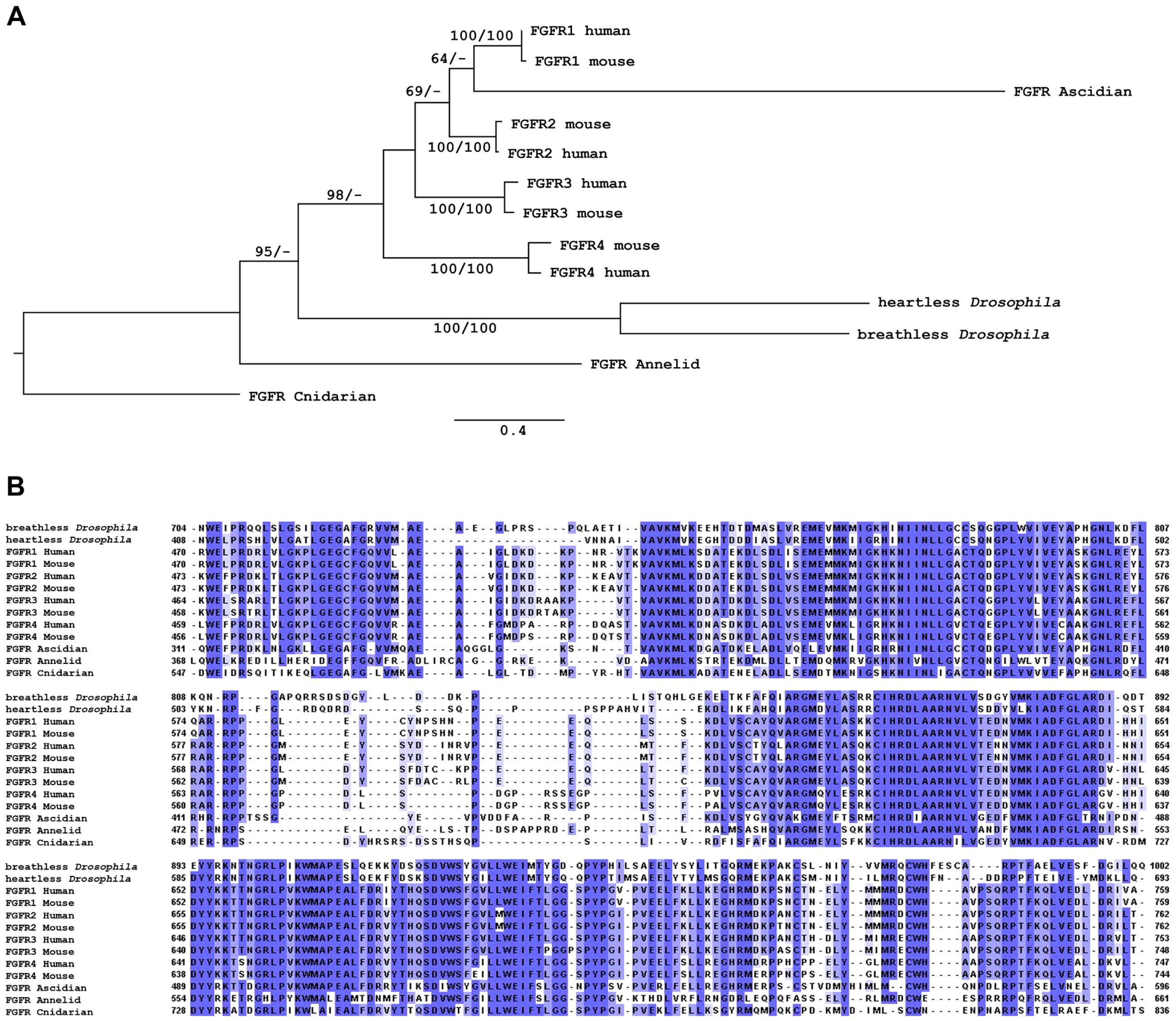

Fig. S4. Phylogenetic comparison of *Drosophila* FGF receptor proteins. (A) Phylogenetic tree, and (B) partial sequence comparison of Fibroblast growth factor receptor family homologs of *Drosophila melanogaster* breathless and heartless; human and mouse FGFR1, FGFR2, FGFR3 and FGFR4; Ascidian *Ciona intestinalis* FGFR; Annelid *Capitella teleta* FGFR; and Cnidarian *Nematostella vectensis* FGFR. Maximum likelihood (ML) phylogenetic tree in (A) was inferred by the GGDC web server (https://ggdc.dsmz.de/phylogeny-service.php) from a MUSCLE multiple sequence alignment with Randomized Axelerated Maximum Likelihood (RAxML); support values on the branches are from ML bootstrapping with RAxML (left) and from maximum parsimony (MP) bootstrapping with Tree analysis using New Technology (TNT) (right) when larger than 60%. Sequence comparison in (B) was generated using the Kalign neighbour joining algorithm available at the European Bioinformatics Institute (www.ebi.ac.uk/Tools/msa/kalign/). Protein sequences were derived from UniProt, accession numbers Q09147, DROME breathless; Q07407, DROME heartless; P11362, hFGFR1; P16092, mFGFR1; P21802, hFGFR2; P21803, mFGFR2; P22607, hFGFR3; Q61851, mFGFR3; P22455, hFGFR4; Q03142, mFGFR4; R7TKL8, CAPTE FGFR; Q4H3K6, CIOIN FGFR; B2WR86, NEMVE FGFR.

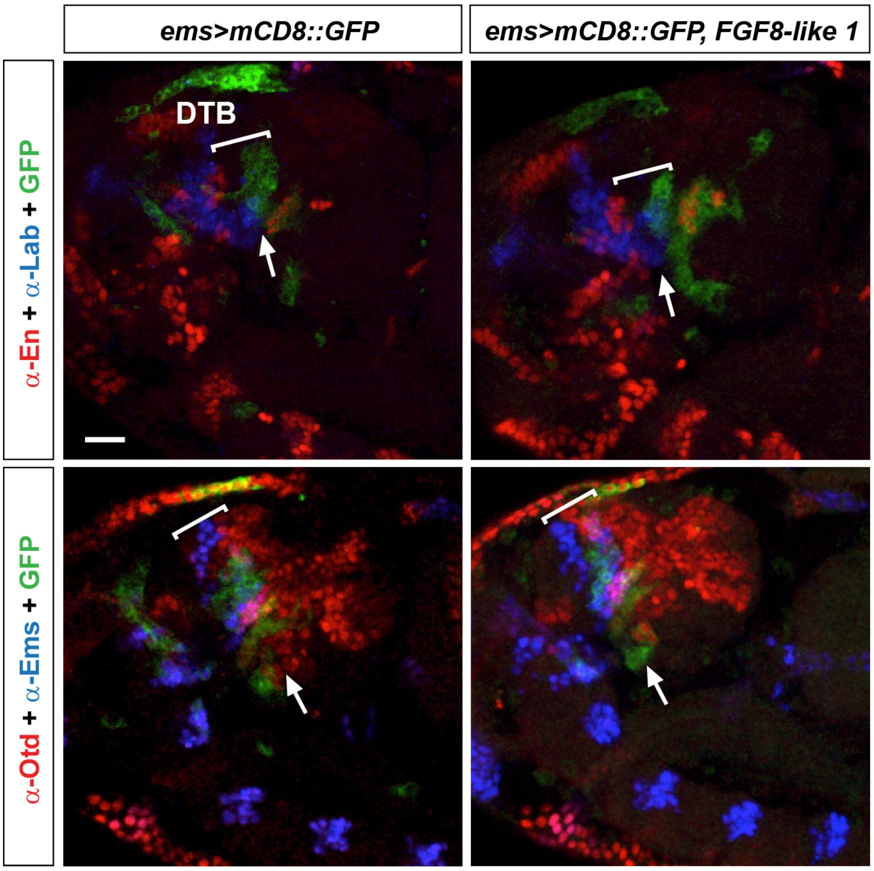

Fig. S5. FGF8 signalling in the embryonic DTB does not exert organiser activity. Ectopic expression of *FGF8-like1* does not affect DTB formation**.** Confocal images of developing embryonic brain of control (*ems-Gal4/UAS-mCD8::GFP*) and experimental flies (*ems-Gal4/UAS-mCD8::GFP; UAS-FGF8-like1)*; anterior is to the left. DTB-specific (bracket) expression patterns of *engrailed* and *labial*, as well as of *otd* and *ems* are unaltered in *ems-Gal4/UAS-mCD8::GFP; UAS-FGF8-like1* flies compared to controls (arrows). Scale bar: 10µm.

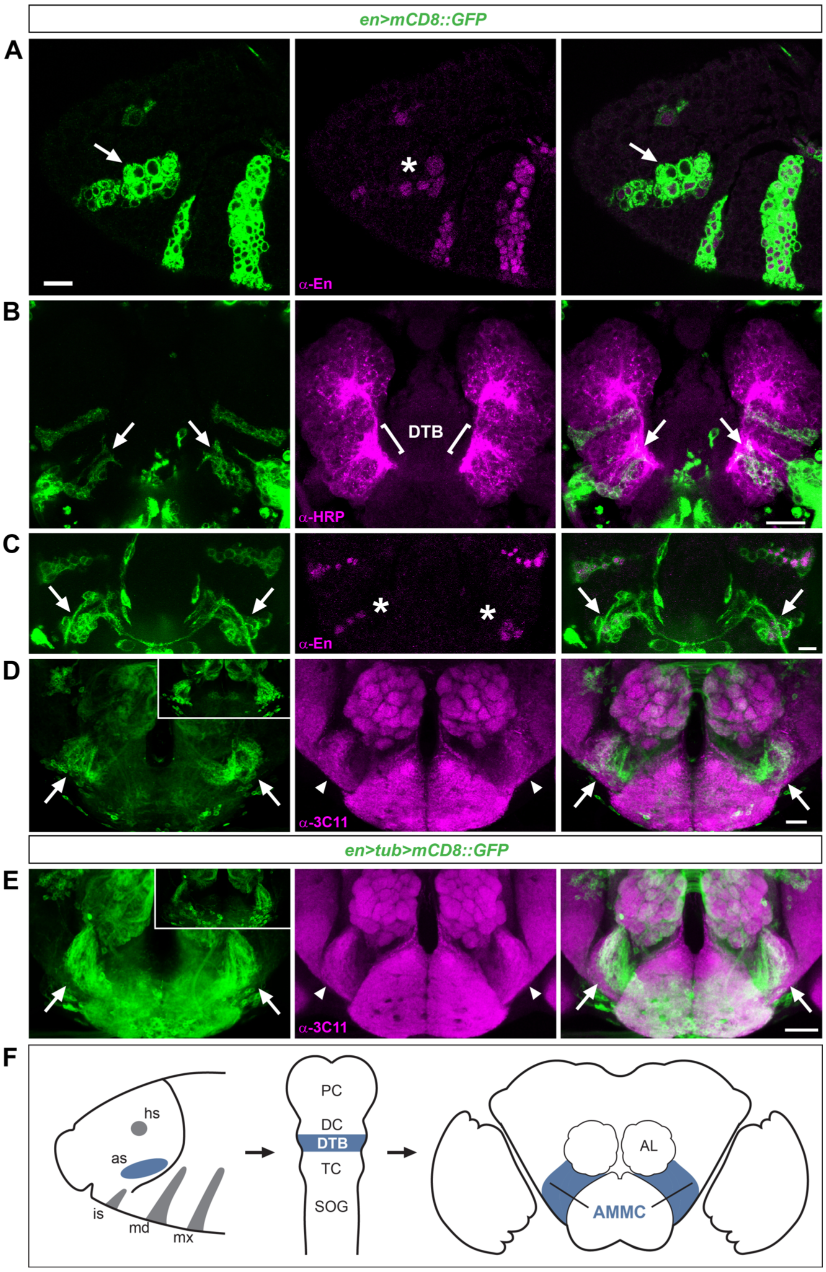

Fig. S6. The embryonic DTB region gives rise to neurons of the AMMC in the adult brain of *Drosophila.* Confocal images of developing embryonic (A-C) and adult brain (D, E); anterior to the left in A; and frontal in B-E. (A) at embryonic stage 11, *en-Gal4/UAS-mCD8::GFP* mediated cell labelling identifies anti-En positive neuroblasts in the procephalic neurectoderm, including NBs (asterisk) of the deutocerebral-tritocerebral neurectoderm boundary (DTB, arrows). (B) by embryonic stage 15, *en-Gal4/UAS-mCD8::GFP* identifies En-NB derived lineages, including DTB lineages (arrows) that (C) express Engrailed; their axonal projections include the developing antennal nerve (arrows). (D) in the adult brain, *en-Gal4/UAS-mCD8::GFP* visualises neurons, projections and axon terminals of the antennal mechanosensory motor centre (AMMC, arrows). (E) lineage tracing of *en* expressing central brain NB lineages. Confocal images of *en>tub>mCD8::GFP* identify AMMC neurons (inset) and axon terminals within the AMMC (arrows). (F) left, schematic of Engrailed expression domains in the procephalic neuroectoderm of stage 11 embryo, including head spot (hs), antennal spot (as) and intercalary spot (is); middle, schematic of stage 15 embryonic brain with protocerebrum (PC), deutocerebrum (DC), tritocerebrum (TC) and subesophageal ganglion (SOG); right, adult brain; AL, antennal lobe. Scale bars: 7.5μm (A), 10μm (B), 50μm (D, E).

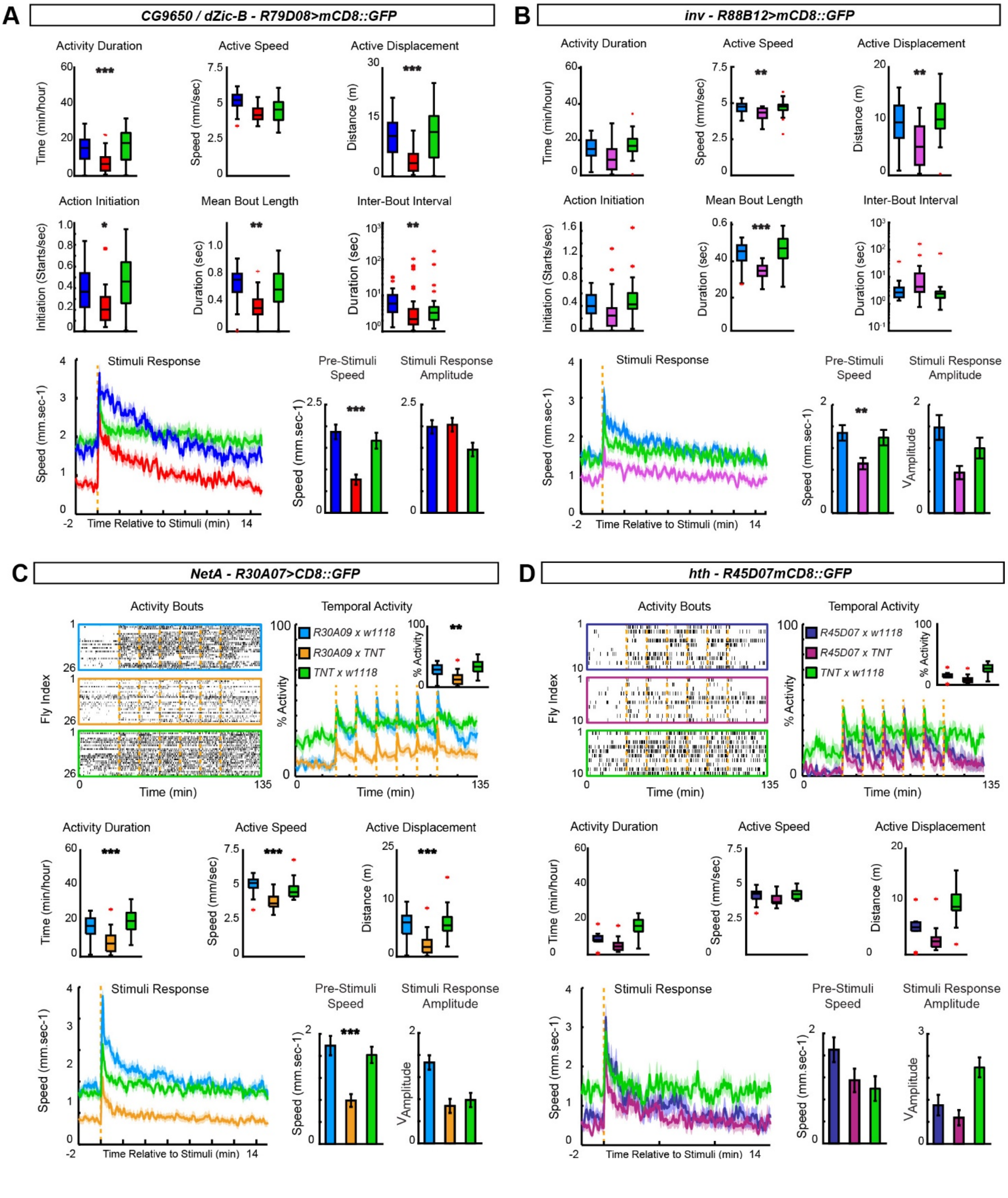

Fig. S7. Motor coordination in *Drosophila* is mediated by DTB-derived AMMC neurons. Motor kinematics and stimulus response (lower lane) of (A) *R79D08>TNT* and (B) *inv R88B12>TNT* with respective UAS/+ and Gal4/+ control flies; colour code indicate genotypes. Motor behaviour of (C) *R30A07>TNT* and (D) *R45D07>TNT* with respective UAS/+ and Gal4/+ control flies; top left, raster plots of activity bouts, each lane one individual fly; middle lane, motor kinematics; bottom lane, stimulus response. Mean ± Standard Error of the Mean (SEM), asterisks indicate p<0.05 (*), p<0.01 (**) or p<0.001 (***).

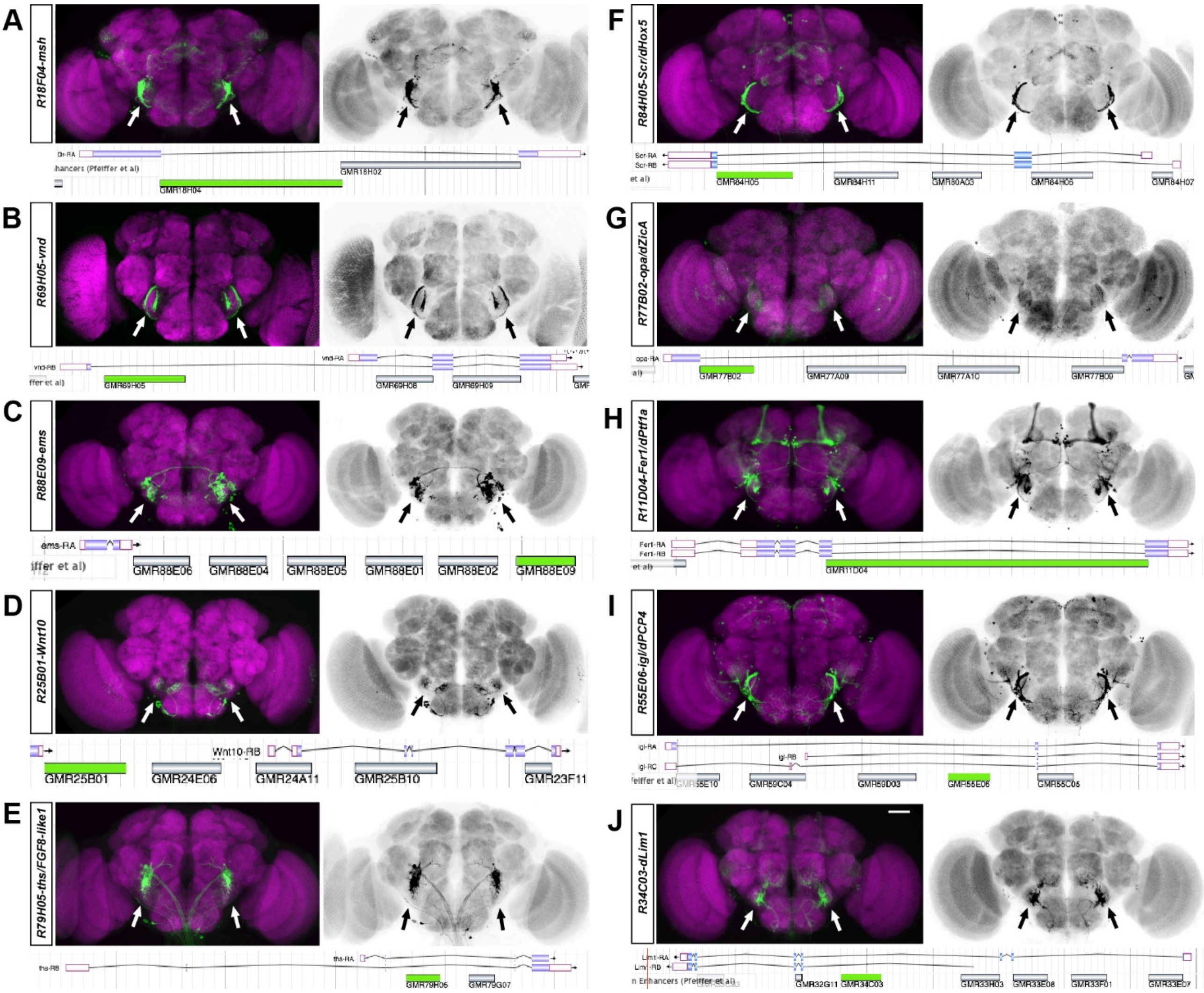

Fig. S8. Non-coding regulatory elements direct AMMC-specific expression in adult brain of *Drosophila.* Non-coding cis-regulatory elements (CREs) identified by the Janelia FlyLight project drive Gal4 expression of *UAS-mCD8::GFP* targeted to the antennal mechanosensory motor centre (AMMC). Confocal images of adult brains immunolabelled with anti-Brp/nc82 (magenta) and mCD8::GFP (green); black and white images are inversions to highlight GFP-labelled structures; dorsal is up (original confocal stacks are derived from the Janelia FlyLight repository). (A) *msh*-specific *R18F04>mCD8::GFP*. (B) *vnd*-specific *R69H05>mCD8::GFP*. (C) *ems*-specific *R88E09>mCD8::GFP.* (D) *Wnt10*-specific *R25B01>mCD8::GFP.* (E) *ths/FGF8-like1*-specific *R79H05>mCD8::GFP.* (F) *Scr*-specific *R84H05>mCD8::GFP.* (G) *opa/dZic4*-specific *R77B06>mCD8::GFP.* (H) *Fer1/dPtf1a*-specific *R11D04>mCD8::GFP.* (I) *igl/dPCP4*-specific *R55E06-Gal4/UAS-mCD8::GFP*. (J) *dLim1*-specific *R34C03>mCD8::GFP* (Janelia FlyLight database, with permission; data from ref. 49). Arrows indicate GFP-labelled AMMC-specific neurons and/or projections/arborisations targeted by respective CRE. The genomic position of each CRE is depicted underneath the respective labelled brain and highlighted in green. Scale bar in J: 50μm.

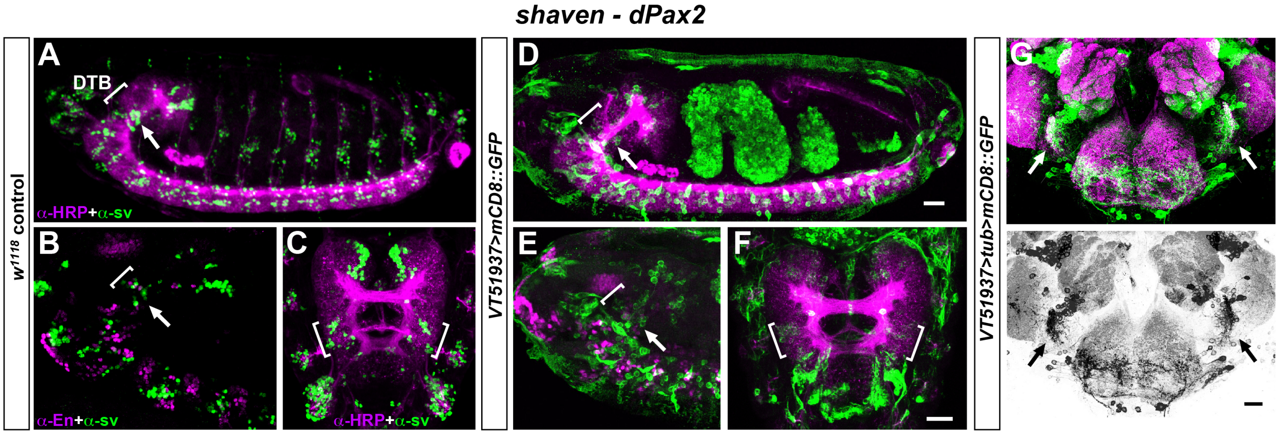

H CRE sequence comparison

```
shaven   CAACCAATTTTAGTT----TTCAG---CCCTCCACTCA------AATACATTGAAATGTCCA-TGAAGCGGGATGTACTTTT---CGCGCA----TCCCA
mPax2    CAAGCAATTTTAATATTTCTTCAGGGACTTTCCAGCAACAGCACAAGTCATTAATTCACCCTCCCAAATTGCTTATTCAATAGCAGGAACATGGCTCCTG
hPAX2    CAAGCAATTTTAATATTTCTTCAGGGACTTTCCAGCAACAGCACAAGTCATTAATTCACCCTCCCAAATTGCTTATTCAATAGCAGGAACATGGCTCCTG
         *** ******** *     *****   *  ****   *      **  **** *     **    **   *  * * *  *     *  **    ***

shaven   AACATATGTCTCA--A-TTGTGTGCACTGTCGAAATATCCAATTGCTCATCCCG-TTTCAAGCT-GGTACTCAAAAAT--ATATAAAAC-CTGAAACTGT
mPax2    AATAA-AGTCCCAGAATTTATGTG-AC--TTGCACGAGTCAGGAGGTCAAACAACTGTTATGGAGCGAAGTTAAAAATCCAAATAAAATATTGACACTTT
hPAX2    AATAA-AGTCCCAGAATTTATGTG-AC--TCGCACGAGTCAGGAGGTCAAACAACTGTTATGGAGCGAAGTTAAAAATCCAAATAAAATATTGACACTTT
         ** *   *** **  * ** **** **  * * *  *  **   * ***  *   * * * *    * * * ******  * ******   *** *** *

shaven   TGTCTTAAGG-GCACAAACTAGGCAGAGTTAACTTCTGCCCTTACAACCCCCTGTAGCATATGGTAACCTTAACAATCTGTAAAAAAAACCTAAAAGATA
mPax2    TTTGGGGAGGGGAAGAGAGGAGGCCAAGGTATTT------AAAATAGGTTTTTGTTGCA-ATG----CCTC------CAGGATAAAGAAT-GGAAGGGGA
hPAX2    TTTGGGGAGGGGGAGGTGGGAAGCAAAGGTATTT------AAAATAGGTTTTTGTTGCA-ATG----CCCC------CAGGATAAAGAAT-GGAAGGAGA
         * *    *** * *      * **  ** **  *         * *      *** *** ***    **        * * * *** **    ** *  *

shaven   CTTAAATCAATGCTATCCCGGAGGTCCTATGAGTTTTAATAGGTGTATTTGGGTACCGCATTTTGTAATTATTAAAGTTTATTGCA-AA
mPax2    CTGAAGACAAG------TTGGAGTT--TATAAAA-TCAATGGAATTTATTGCACATC-TACTTGGCATTTAATAAT-TCTCTCATT-TC
hPAX2    CTGAAGACAAG------TTGGAGTT--TATAAAA-TCAATGGAATTTATTGCACAGC-TACTTGGCATTTAATAAT-TCTCTCATTTCC
         ** **  ***         **** *  *** *   * *** *   *  ***   * *  * ** * * *** ***  * * *
```

I Intragenic location of CRE

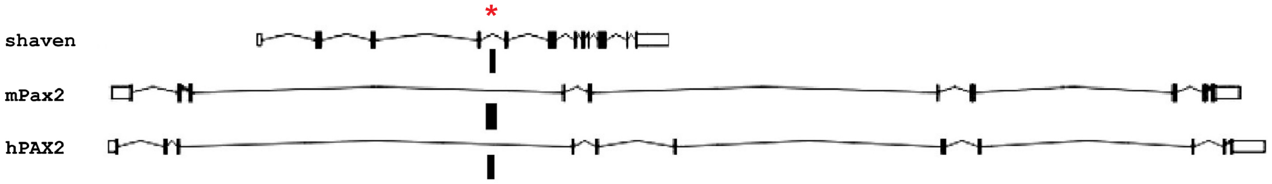

Fig. S9. Conserved non-coding regulatory sequence of *shaven/dPax2* directs DTB-AMMC specific expression in *Drosophila.* (A-C) confocal images of anti-sv/dPax2 immunolabelling (green, yellow) in embryonic CNS (A, lateral, anterior to left) and brain (B, lateral; C, frontal) co-immunolabelled with anti-HRP (A, C, magenta) or anti-Engrailed (B, magenta); brackets indicate deutocerebral-tritocerebral boundary (DTB) region; note sv/dPax2 expression within DTB (arrows). (D-F) lacZ expression directed by *sv/dPax2*-specific regulatory element VT51937, including DTB (brackets, arrows). (G) *sv/dPax2-VT51937>tub>mCD8::GFP* mediated genetic tracing identifies AMMC neurons and projections (arrows). (H) Comparison of non-coding regulatory sequences (CREs) of *Drosophila melanogaster sv/dPax2* that comprise parts of *VT51937*, mouse *mPax2* and human *hPAX2* (asterisks denote identical amino acids), and (I) their comparable intragenic locations (black bar, see arrow above exon-intron annotation) within genomic loci of, respectively, *sv/dPax2*, *mPax2* and *hPAX2.* Scale bars: 20μm.

invected - ENGRAILED 2

A CRE Sequence comparison

```
invected  TGAAAACTGAAGACTGCAACCAGGGACCACACACGACGATCGAGGCTGTGCCACAAGCTGCTCTGGGCAACGGAAGGCGGCAACATCGACAGTAGTTTTG
mEn2      TCAAACTTGCATA-TGCAACGAGGT-----GCTG--------------------GA---GCTTTGT---------GGCGGAAATAT-------AATTAAA
hEN2      TCAAACTTGAATA-TGCAATGAGAC-----GCGG--------------------GA---GCTTTGT---------GGCGGAAATAT-------AATTAAA
          * ***  ** * * *****  **        *                       *   *** **          ***** ** **       * **

invected  CGGCTCGTTTGCTGCGTTGACTCTTCGCCGAGTCCCGTGATCATGGACATGATCATCATGACGTACGATGATCGTGGGGTCCGCAGAACGTCATCATCGC
mEn2      -----------CTG-------------GTGAGA--------GATGTAAAAGA------TGA-------AGAA--TGGG---------ATGACATCAGA--
hEN2      -----------CTG-------------GGGAAA--------GATGTAAAAGC------TGA-------AGAA--TGGG---------ATGACATCAGG--
                     ***               **           *** * * *       ***        **   ****         * * *****

invected  GAAGATCTTCCTCCTCCTGATGCGGCGCCAAATTGCAGCAAAGTAAAACAGCGTTTAGCATTGTGCTTGTAATGATCTTGGTCAGGCA-ATCCGAGGGCC
mEn2      -CTGATTTCAC-ACT--TG--GCA--GTCCGATAGCTGGG--CCCAAGCCGGG------------CTTGCCACG-------CAAGGCAGATCAAAGTGCT
hEN2      -CTGATTTCAC-ACT--CG--GCA--GTCGGATCGCTGGG--CCCAAGCCGCGCT----------CTTGCCACG-------CAAGGCAGATCAAAGTGCC
             *** *  *  **   *  **   * *  ** ** *       ** * * *            ****  * *         ***** ***  ** **

invected  TGGCGTCCACAAATCTACAGAAGAACCCAAGTCTGGAGATTCAAAGAGAGGTCCCCGAGATTCTGGGCGACTTAAGAGAAGAACAGCGCAACTCGTTCGC
mEn2      CTGCCACCACTAA--------A-AAGGCAAA--GGGAGACTTAAGTA------TGCTA-ATC--------CCCAGGACAAATA-----------------
hEN2      CTGCCACCGCTAA--------A-AAGGCAAA--AGGGGACTTAAGTA------TGCTA-ATC--------CCCAGGACAAATA-----------------
            **  ** * **        * **  ***    ** ** * **  *        * * **         *  * ** **  *

invected  TGAGTTAGCGCCTTTGCAATGGGTGTAATAATTTATGTATTTTGATTAGGCCGAAGTTTCTGTGACTGTGGCTCGGCTTTTGCCTTTTAATTGATAAGTG
mEn2      -----------------------------TATTTA---ATCTTGTTAGAATACAAGTTAATG---CTGCAGTGCAG---T-GGCT-GAACTTGGTCAGGC
hEN2      -----------------------------TATTTA---ATCTTGTTAGAATACAAGTTAATG---CTGCAGCTCAG---T-GGCT-GAACTTGGTCAGAC
                                      ** ****   ** *** *       *****  **   ***  *  * *   * * **   * *** * **

invected  TGTTTGAGATACTTT-TTAATGAAGCTAATGAAACATGGTGAGCTTTCAGATATGTCTATATCTTTCAAGGGT-TTAGGCACTCCGGTAATTTAATAAAC
mEn2      AGG-AGAGACCCATTTTTATCTTAGCTA-TGAA------TGAGGT---GAA--------GCTCTT-----GGTTTTATTCA--TCAGTAATAAAA-GTAC
hEN2      TGT-AGAGATCCATTTTTATTTTAGCTA-TGAA------TGAGCT---AGA--------CCTCTT-----GGT-TTATTCA--TCGGTAATAAAA-GTAA
           *   ****  * ** ***    ***** ****      **** *    *           ****     *** ***  **   * *****  **   *

invected  ATTTAAATCTTTAAATGAA
mEn2      ATTTA-----TAAAACGAA
hEN2      ATTTA-----CAAAACGAA
          *****       *** ***
```

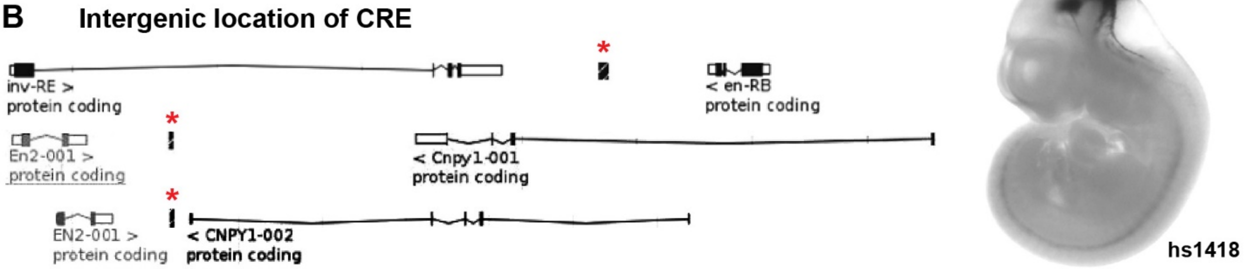

Fig. S10. Conserved non-coding regulatory sequence of *engrailed/invected* and *ENGRAILED 2* directs MHB-specific expression in mouse. (A) sequence comparison of non-coding cis regulatory elements (CREs) of *Drosophila melanogaster inv/dEn2*, mouse *Engrailed 2 (mEn2)* and human *ENRGAILED 2 (hEN2)* aligned towards consensus sequence with human CRE hs1418 reported in VISTA enhancer database (asterisks denote identical amino acids). (B) comparable intragenic CRE location (black bar) of, respectively, *Drosophila inv/dEn2*, mouse *mEn2* and human *hEN2*. Note inverted orientation of *mEn2* and *hEN2* compared to *Drosophila invected* and *engrailed.* (C) whole-mount E11.5 mouse embryo (VISTA database, with permission; data from ref. 54) showing LacZ expression pattern in the midbrain hindbrain boundary region (MHB, arrow) targeted by human EN2-specific CRE, *hs1418*.

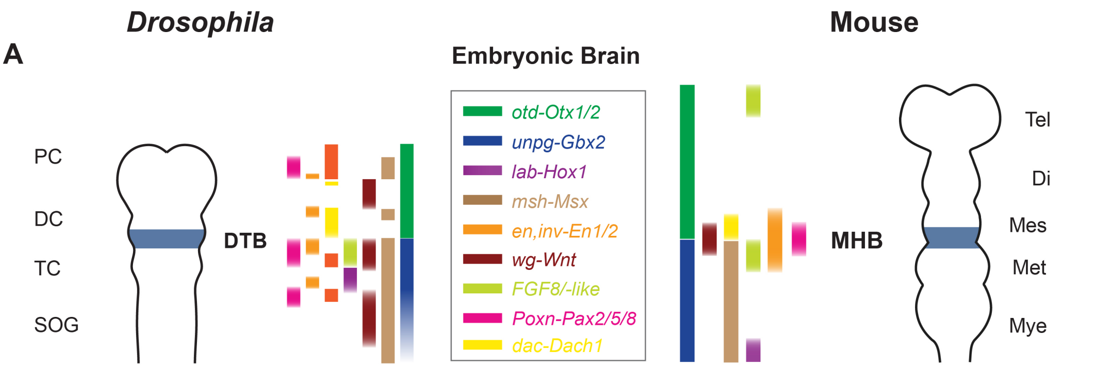

B **Developmental Genetics**

DTB development		MHB development
Formation of proto- and deutocerebrum	*otd-Otx1/2*	Formation of fore- and midbrain
Restriction of *otd* and *lab* expression positioning of DTB	*unpg-Gbx2*	Restriction of *Otx2* and *Hox1b* expression, positioning of MHB
Formation and specification of DTB	*msh-Msx*	Midbrain formation
DTB formation	*en,inv-En1/2*	MHB formation
DTB formation	*wg-Wnt*	MHB formation
Maintenance of DTB and derived structures	*pyramus & thisbe - FGF8*	Formation and maintenance of MHB and derived structures, organizer activity
Specification of DTB	*Poxn-Pax2/5/8*	Formation and specification of MHB

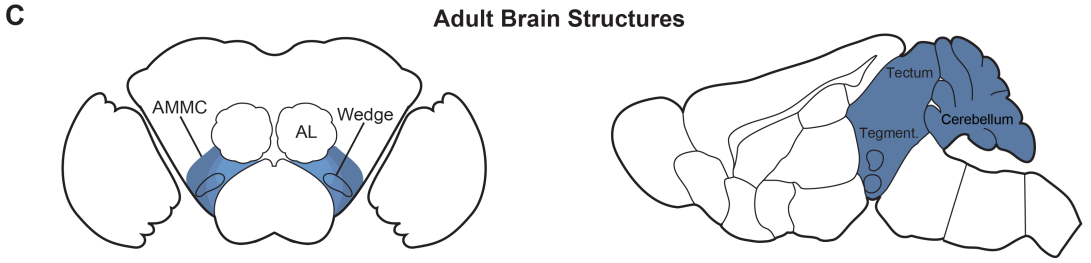

D **Neural Circuits & Behaviour**

DTB-Derived	MHB-Derived
Antennal mechanosensory and motor centre (AMMC), Wedge	*Cerebellum, Tectum, Tegmentum*
Sensory Integration	Sensory Integration
Control of Auditory & Visual Responses	Control of Auditory & Visual Responses
Vestibular pathways	Vestibular pathways
Balance	Balance
Motor Coordination	Motor Coordination
Body Posture	Muscle Tone & Body Posture

Fig. S11. Comparative analysis of ground pattern organization of the *Drosophila* DTB and the mouse MHB. (A) schematic of embryonic *Drosophila* and mouse brain showing expression patterns of homologous genes along neuraxes; anterior is to the top. (B) summary of DTB/MHB-related functions of gene homologues. (C) schematic of adult *Drosophila* and mouse brain; highlighted in blue are structures derived from, respectively, DTB and MHB regions. (D) DTB and MHB derived neural circuits in the adult brain and behavioral manifestations mediated by DTB/MHB-derived neural circuits. Abbreviations: AL, antennal lobe; AMMC, antennal mechanosensory motor center; dc, deutocerebrum; di, diencephalon; DTB, deutocerebral-tritocerebral boundary; hb, hindbrain; mb, midbrain; Mes, Mesencephalon; Met, Metencephalon; Mye, Myelencephalon; MHB, midbrain hindbrain boundary; pc, protocerebrum; SOG, Subesophageal ganglion; tc, tritocerebrum; tel, telencephalon.

Table S1. Statistical analysis of SING data

All genotypes	Genotype tested	Tests done (alpha=0.05)<br>KW = Kruskal-Wallis with post hoc Mann-Whitney U test<br>ANOVA = ANOVA with post hoc Tukey-Kramer	p value for group test (alpha = 0.05)	**p values for pairwise tests**<br>(alpha=0.05, Dunn-Sidak corrected value = 0.01695)<br>e.g. ctrl1 = TNT/w or nbc/w, ctrl2 = Gal4/w,<br><u>subject = Gal4>TNT</u><br>N/A signifies that no pairwise test was carried out because the group test did not show significant differences between groups			Significance<br>(requires $p \leq 0.05$ for group test AND ctrls not significantly different to one another AND subject significantly different to both controls)	Difference compared with controls
				ctrl1 vs ctrl2	subj vs ctrl1	subj vs ctrl2		
	R11A07>TNT	ANOVA	0.0000	Not Significant	Significant	Significant	Significant	Decrease
	R37F03>TNT	KW	0.0000	0.0199	0.0021	0.0000	Significant	Decrease
	R52F05>TNT	ANOVA	0.0001	Not Significant	Significant	Significant	Significant	Decrease
	R51D11>TNT	ANOVA	0.0000	Not Significant	Significant	Significant	Significant	Decrease
	R79D08>TNT	KW	0.0000	0.6454	0.0000	0.0000	Significant	Decrease
	R70G01>TNT	ANOVA	0.0000	Not Significant	Significant	Significant	Significant	Decrease
	R24C06>TNT	ANOVA	0.0000	Not Significant	Significant	Significant	Significant	Decrease
	R45D07>TNT	ANOVA	0.0000	Not Significant	Significant	Significant	Significant	Decrease
	R22B11>TNT	KW	0.0000	0.1594	0.0000	0.0000	Significant	Decrease
	R55C02>TNT	ANOVA	0.0006	Not Significant	Significant	Significant	Significant	Decrease
	R87G01>TNT	KW	0.0016	0.4323	0.0047	0.0012	Significant	Decrease
	R30A07>TNT	ANOVA	0.0000	Not Significant	Significant	Significant	Significant	Decrease
	R19E09>TNT	KW	0.0000	0.0166	0.0000	0.0000	Not Significant	No difference
	R25B01>TNT	KW	0.0000	0.0222	0.0000	0.0000	Significant	Decrease
	R17A10>TNT	KW	0.0007	0.0498	0.0123	0.0006	Significant	Decrease

Table S2. *Drosophila* genes, their vertebrate homologs and role in the development of the cerebellum and cerebellar neural circuits.

Drosophila	Vertebrate	Ref.	Embryonic	Postnatal
orthodenticle	*Otx1*	(1)	-	EGL
	Otx2	(1)	Cerebellar precursor cells	EGL and IGL
unplugged	*Gbx2*	(2)	MHB	-
vnd	*Nkx6.1*	(3)	MHB	?
msh	*Msx2*	(4)	MHB	IGL, Purkinje cells
engrailed/invected	*En-1/En-2*	(5)	MHB	Granule cells
wingless	*Wnt-1*	(5)	Rostral to MHB	
	Wnt-3	(5)	MHB	Purkinje cells
	Wnt-7a	(5)	uniform brain expr.	Granule cells
pyramus/thisbe	*FGF8*	(6)	MHB	Cerebellum
shaven/dPax2	*Pax2*	(5)	MHB	Cerebellum
Poxn	*Pax2/5/8*	(5)	MHB	Granule cells
Enhancer of split	*HES1/HES-3*	(7)	MHB	Purkinje cells
Eyeless	*Pax6*	(8)	Cerebellum	EGL, granule cells
Odd-paired	*Zic1/2*	(9)	Granule cell precursor	Granule cells
CG9650	*Zic1/2*	(9)	Granule cell precursor	Granule cells
atonal/cato	*Math1*	(10)	EGL	Granule cells
	NeuroD	(11)	EGL	IGL, granule cells
Sex combs reduced	*HoxA5*	(12)	Hindbrain	Purkinje cells
Fer1	*Ptf1a*	(13)	Cerebellum	GABAergic neurons
igloo	*PCP4*	(14)	Cerebellum	Purkinje cells

Modified and updated after (15-17). Abbreviations: EGL, external granule cell layer; IGL, internal granule cell layer; MHB, midbrain hindbrain boundary; Ref., reference.

Movie S1. Startle-induced negative geotaxis of *R52F05/w*^{1118} control flies.

Movie S2. Startle-induced negative geotaxis assay of *R52F05>TNT* flies.

Additional data set 1 (separate file). Super-conserved CRE sequences for *shaven/PAX2*.

Additional data set 2 (separate file). Super-conserved CRE sequences for *invected/engrailed* and *EN2*.

Additional data set 3 (separate file). Super-conserved CRE sequences for *dachshund/DACH1*.

SI References

1. Frantz, G.D., Weimann, J.M., Levin, M.E., McConnell SK. *Otx1* and *Otx2* define layers and regions in developing cerebral cortex and cerebellum. *J. Neurosci.* **14**, 5725-5740 1994.
2. Wassarman, K.M. *et al.* Specification of the anterior hindbrain and establishment of a normal mid/hindbrain organizer is dependent on *Gbx2* gene function. *Development* **124**, 2923-2934 (1997).
3. Ma, P., Xia, Y., Ma, L., Zhao, S., Mao, B. Xenopus *Nkx6.1* and *Nkx6.2* are required for mid-hindbrain boundary development. *Dev. Genes Evol*. **223**, 253-259 (2013).
4. Satokata, I. *et al. Msx2* deficiency in mice causes pleiotropic defects in bone growth and ectodermal organ formation. *Nat. Genet.* **24**, 391-395 (2000).
5. Joyner, A.L. *Engrailed*, *Wnt* and *Pax* genes regulate midbrain--hindbrain development. *Trends Genet.* **12**, 15-20 (1996).
6. Sato, T. & Joyner, A.L. The duration of FGF8 isthmic organizer expression is key to patterning different tectal-isthmo-cerebellum structures. *Development* **136**, 3617-3626 (2009).
7. Hirata H, Tomita K, Bessho Y, Kageyama R. *Hes1* and *Hes3* regulate maintenance of the isthmic organizer and development of the mid/hindbrain. *EMBO J.* **20**, 4454-4466 (2001).
8. Swanson, D.J., Goldowitz, D. Experimental *Sey* mouse chimeras reveal the developmental deficiencies of Pax6-null granule cells in the postnatal cerebellum. *Dev. Biol.* **351**, 1-12 (2011).
9. Aruga, J., Inoue, T., Hoshino, J., Mikoshiba, K. *Zic2* controls cerebellar development in cooperation with *Zic1*. *J. Neurosci*. **22**, 218-25 (2002).

10. Ben-Arie, N. *et al. Math1* is essential for genesis of cerebellar granule neurons. *Nature* **390**, 169-172 (1997).
11. Miyata, T., Maeda, T., Lee, J.E. *NeuroD* is required for differentiation of the granule cells in the cerebellum and hippocampus. *Genes Dev.* **13**, 1647-1652 (1999).
12. Sanlioglu, S., Zhang, X., Baader, S.L., Oberdick, J. Regulation of a Purkinje cell-specific promoter by homeodomain proteins: repression by *engrailed-2* vs. synergistic activation by *Hoxa5* and *Hoxb7*. *J. Neurobiol.* **36**, 559-571 (1998).
13. Hoshino, M. Molecular machinery governing GABAergic neuron specification in the cerebellum. *Cerebellum* **5**, 193-198 (2006).
14. Wei, P., Blundon, J.A., Rong, Y., Zakharenko, S.S., Morgan, J.I. Impaired locomotor learning and altered cerebellar synaptic plasticity in *pep-19/PCP4*-null mice. *Mol. Cell. Biol.* **31**, 2838-2344 (2011).
15. Oberdick, J., Baader, S.L., Schilling, K. From zebra stripes to postal zones: deciphering patterns of gene expression in the cerebellum. *Trends Neurosci.* **9**, 383-390 (1998).
16. Butts, T., Green, M.J., Wingate. R.J. Development of the cerebellum: simple steps to make a 'little brain'. *Development* **141**, 4031-4041 (2014).
17. Leto, K. *et al.* Consensus Paper: Cerebellar Development. *Cerebellum* **15**, 789-828 (2016).

SUPPLEMENTARY DATA SET S1

shaven/PAX2 conserved CRE sequence

***sv/dPax2* VT51937**

```
caacatcatcgtagaattttggccgtgccacgtggcagttcctaagtcgacactgtcccacattgctattatttacgcgcatccacttt
gaccataatttatgttggtagcagtcaacgaggccaacgagagcagctggaaaacctttaatcccatgttttatttgaaattagctgaa
ccctgtttaaatgtaaattttatgaaattttaaaaatgttaaagcccaaatagccgaacgtcctaaaaacatgtaattacgaaggtttt
tcttcaatcttacgattagtcgttttggaactatggaatatggttgtcccgtgttactcgtacctgttatggctttcaaacaagactag
tttggttaggaacgaatatggacccttattataaaatttttcgaagaataatgttacaaaatgttctttatatttaacaaaaagcata
caagtcattatctgttgatcgagtatttcaaatgtcaagtctcatctctattctgcatgctagaaaatgtaattttaattttaatttt
aaccgatctcaacacgtgcagttataatcaattcgattcgtgttaccgccgggtcccgcaaaaagtaacaaacgtctagattggcatgt
ggccgctggtaatgcaaaagttgcgacggacaagggtaattcatggtataatcaaggaggaagaaaaatttcataatgcgacaatgtgt
gatgtgatggcacttgaacgatgattagggtggcggtgattcaatcgaataaaaattgtgcgtacggcggtgccaggactccccttata
ctgattcccaggagactgtcacttaataaagtgactttcatagcactcaaccaatttagttttcagccctccactcaaatacattgaa
atgtccatgaagcgggatgtacttttcgcgcatcccaaacatatgtctcaattgtgtgcactgtcgaaatatccaattgctcatcccgt
ttcaagctggtactcaaaaatatataaaacctgaaactgttgtcttaagggcacaaactaggcagagttaacttctgcccttacaaccc
cctgtagcatatggtaaccttaacaatctgtaaaaaaaacctaaaagatacttaaatcaatgctatcccggaggtcctatgagttttaa
taggtgtatttgggtaccgcattttgtaattattaaagtttattgcaaaattgtacacaaatactaacacgacacgtttggcatctctt
gaatatgcagggtgtgtgctggacagaaggacaagccatgtccatgtatggactgaccaatattatttatatatctatattgcctcggg
aacgctttttacttgttacaacgaattttactttctataacattttattatcgtattagtataaaataataatacgataatattacga
ttacgattagtacgatctttaaaaacttattacatagttttgatttatatattttgaccacaaaaggcctgtaagacttagtagagcca
ccaaaactttaagtataagtactgaagtcataattagagattataaataacaatgatttacctacctaccctcatgggtaggtgatttt
aagtaatatgaacgttaaagtgttcatggctaacctggggaacagtcacttcttggatatctccatcttccttgcaagaagtgcaaggg
gaaccatagtggtctatcctcttttaaatattatatgtatgtacaactgattgtttattgtagttagttgtgtacaattgatttgttga
taagtatgtaaaaagtagaagaaaacgttctgacccaataaagttcttataatcttggttaggatcacttgccgagtgattgtggccat
gtccaaaactgttaaatatataattcttttctaactttattacaagactatttctgatccttcgctaacaaaatgttgaaattgggtaa
gggggatctgatagagactaggaactattgcggttcctattatttttagtaggcttaaataaaatttaccatttcccttctaaccgtg
aagagcgttgattttatttaactaagtcacttaaaatcttttcacttaatttcagaattgtaaggaataaagccgccgagaaagccaaa
cacgtacatcatcaccagcagcatcatgtttctcagagtctgggtgggggcatattgccacggaaagtgttgacagcagcacagga
```

Green highlighted sequence depicts sv/Pax2 conserved sequence

EMBOSS MATCHER sv conserved against VT51937

```
sv_CNS_CRE1        1 CAACCAATTTTAGTTTTCAGCCCTCCACTCAAATACATTGAAATGTCCAT     50
                     ||||||||||||||||||||||||||||||||||||||||||||||||||
sv_VT51937       849 CAACCAATTTTAGTTTTCAGCCCTCCACTCAAATACATTGAAATGTCCAT    898

sv_CNS_CRE1       51 GAAGCGGGATGTACTTTTCGCGCATCCCAAACATATGTCTCAATTGTGTG    100
                     ||||||||||||||||||||||||||||||||||||||||||||||||||
sv_VT51937       899 GAAGCGGGATGTACTTTTCGCGCATCCCAAACATATGTCTCAATTGTGTG    948

sv_CNS_CRE1      101 CACTGTCGAAATATCCAATTGCTCATCCCGTTTCAAGCTGGTACTCAAAA    150
                     ||||||||||||||||||||||||||||||||||||||||||||||||||
sv_VT51937       949 CACTGTCGAAATATCCAATTGCTCATCCCGTTTCAAGCTGGTACTCAAAA    998

sv_CNS_CRE1      151 ATATATAAAACCTGAAACTGTTGTCTTAAGGGCACAAACTAGGCAGAGTT    200
                     ||||||||||||||||||||||||||||||||||||||||||||||||||
sv_VT51937       999 ATATATAAAACCTGAAACTGTTGTCTTAAGGGCACAAACTAGGCAGAGTT   1048

sv_CNS_CRE1      201 AACTTCTGCCCTTACAACCCCCTGTAGCATATGGTAACCTTAACAATCTG    250
                     ||||||||||||||||||||||||||||||||||||||||||||||||||
sv_VT51937      1049 AACTTCTGCCCTTACAACCCCCTGTAGCATATGGTAACCTTAACAATCTG   1098

sv_CNS_CRE1      251 TAAAAAAAACCTAAAAGATACTTAAATCAATGCTATCCCGGAGGTCCTAT    300
                     ||||||||||||||||||||||||||||||||||||||||||||||||||
sv_VT51937      1099 TAAAAAAAACCTAAAAGATACTTAAATCAATGCTATCCCGGAGGTCCTAT   1148

sv_CNS_CRE1      301 GAGTTTTAATAGGTGTATTTGGGTACCGCATTTTGTAATTATTAAAGTTT    350
                     ||||||||||||||||||||||||||||||||||||||||||||||||||
sv_VT51937      1149 GAGTTTTAATAGGTGTATTTGGGTACCGCATTTTGTAATTATTAAAGTTT   1198

sv_CNS_CRE1      351 ATTGCAAA    358
                     ||||||||
sv_VT51937      1199 ATTGCAAA   1206

Identity: 358/358 (100.0%) - # Similarity:   358/358 (100.0%)
Length: 358 - Gaps: 0/358 (0.0%) - Score: 1790
```

Human PAX2 - GRCh38 10:100767614-100768004

```
CAAGCAATTTTAATATTTCTTCAGGGACTTTCCAGCAACAGCACAAGTCATTAATTCACCCTCCCAAATTGCTTATTCAATAGCAGGAA
CATGGCTCCTGAATAAAGTCCCAGAATTTATGTGACTCGCACGAGTCAGGAGGTCAAACAACTGTTATGGAGCGAAGTTAAAAATCCAA
ATAAAATATTGACACTTTTTTGGGGAGGGGGAGGTGGGAAGCAAAGGTATTTAAAATAGGTTTTTGTTGCAATGCCCCCAGGATAAAGA
ATGGAAGGAGACTGAAGACAAGTTGGAGTTTATAAAATCAATGGAATTTATTGCACAGCTACTTGGCATTTAATAATTCTCTCATTTCC
```

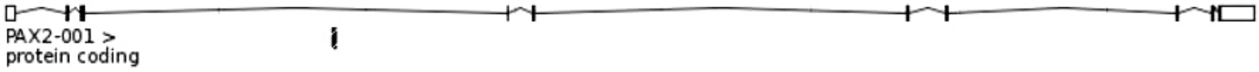

Mouse Pax2 - GRCm38 19:44778358-44778747

```
CAAGCAATTTTAATATTTCTTCAGGGACTTTCCAGCAACAGCACAAGTCATTAATTCACCCTCCCAAATTGCTTATTCAATAGCAGGAA
CATGGCTCCTGAATAAAGTCCCAGAATTTATGTGACTTGCACGAGTCAGGAGGTCAAACAACTGTTATGGAGCGAAGTTAAAAATCCAA
ATAAAATATTGACACTTTTTTGGGGAGGGGAAGAGAGGAGGCCAAGGTATTTAAAATAGGTTTTTGTTGCAATGCCTCCAGGATAAAGA
ATGGAAGGGGACTGAAGACAAGTTGGAGTTTATAAAATCAATGGAATTTATTGCACATCTACTTGGCATTTAATAATTCTCTCATTTC
```

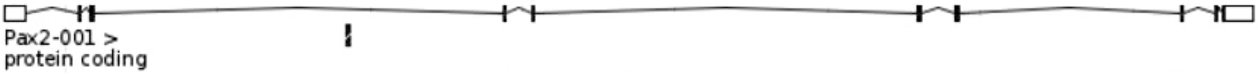

Chicken scaffold >chromosome:Gallus_gallus-5.0:6:17159089:17159892:-1

```
CCTGGCACTGGAAGGGACAGCAAGGATGGAAGGCTGCTGTCAGCTGCACGGTGTGTGCGCAGGAATTCCCCTTCCTCTCCCAAGTGCGG
CTGTTTGTAGCCATTAAAAAGCCCAGGTCCTCATGCTGGGTGTTCAGTGAGTATTGATTCAGTCTGCAAGCAATTTTAATATTTCTTCA
GGGACTTTCCAGCAACAGCACAAGTCATTAATTCACCCTCCCAAATTGCTTATTCAATAGCAGGAACATGGCTCCTGAATAAAGTCCCA
GAATTTATGTGACTCGCATGAGTCAGGAGGTCAAACAACTGTTATGGAGCGAAGTTAAAAATCCAAATAAAATATTGACACTTTTTTGG
GGA
```

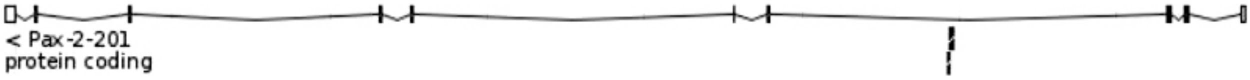

Zebrafish Pax2a - GRCz10 13:29665906-29666048

```
GCACATGCAAATATTAAAATCCACAAATCTAATCTAAAATGGCTTTTTAAGTTCATATAGATGGTTTGTGAAATTGTTTTATTATTATT
ATTATTATTATTAATTTATTGTATTTAATTTTACATTAGTA
```

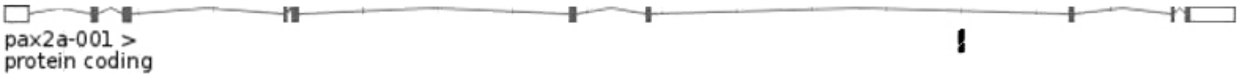

MLAGAN comparison fly-mouse-human sv/Pax2

sequence1	FlyShavenBDGP6_4_1083735_1116126_1:1-32392 (+)
sequence2	mousePax2_GRCm38_19_44769940_44788670_1:1-18731 (+)
sequence3	humanPAX2_GRCh38_10_100759566_100779388_1:1-19823 (+)

Conservation Visible

ATGC CNS

```
sequence1(+)   000021153   TTCATAGCACTCAACCAATTTTAGTTTTCAGCCCTCCACTCAAATACATT   000021202
sequence2(+)   000008430   ----CAGTCTGCAAGCAATTTTAATATT----TCTTCA-----------   000008459
sequence3(+)   000008060   ----CAGTCTGCAAGCAATTTTAATATT----TCTTCA-----------   000008089

sequence1(+)   000021203   GAAATGTCCATGAAGCGGGATGTACTTTTCGCGCATCCCAAACATATGTC   000021252
sequence2(+)   000008460   ----------------GGGA-----CTTTCCAGCAAC---AGCACAAGTC   000008485
sequence3(+)   000008090   ----------------GGGA-----CTTTCCAGCAAC---AGCACAAGTC   000008115

sequence1(+)   000021253   -TCAATTGTGTGCACTGTCGAAATATCCAATTGCTCATCCCGTTTCAAG-   000021300
sequence2(+)   000008486   ATTAATT-----CACCCTC------CCAAATTGCTTATTCAATAGCAGGA   000008524
sequence3(+)   000008116   ATTAATT-----CACCCTC------CCAAATTGCTTATTCAATAGCAGGA   000008154

sequence1(+)   000021301   --CTGGTACTCAAAAATATATAAAACCTGAAACTGTTGTCTTAAGGGCAC   000021348
sequence2(+)   000008525   ACATGGCTCCTGA------ATAAAGTCCCAGAAT-TTATGTGACTTGCAC   000008567
sequence3(+)   000008155   ACATGGCTCCTGA------ATAAAGTCCCAGAAT-TTATGTGACTCGCAC   000008197

sequence1(+)   000021349   AAACTAGGCAGAGTTAACTTCTGCCCTTACAACCCCCTGTAGCATATGGT   000021398
sequence2(+)   000008568   GAGTCAGG--AGGTCAA-----------ACAAC------TGTTATGGAGC   000008598
sequence3(+)   000008198   GAGTCAGG--AGGTCAA-----------ACAAC------TGTTATGGAGC   000008228

sequence1(+)   000021399   AACCTTAACAATCTGTAAAAAAAACCTA---------------------   000021426
sequence2(+)   000008599   GAAGTTAAAAATCCAAATAAAATATTGACACTTTTTTGGGGAGGGGAAGA   000008648
sequence3(+)   000008229   GAAGTTAAAAATCCAAATAAAATATTGACACTTTTTTGGGGAGGGGGAGG   000008278

sequence1(+)   000021427   --------AAAGATACTTAAATCAA--TGCTATCCCGGAGGTCCTATGAG   000021466
sequence2(+)   000008649   GAGGAGGCCAAGGTATTTAAAATAGGTTTTTGTTGCAATGCCTCCAGGA-   000008697
sequence3(+)   000008279   TGGGAAGCAAAGGTATTTAAAATAGGTTTTTGTTGCAATGCCCCCAGGA-   000008327

sequence1(+)   000021467   TTTTAATAGGTGTATTTGGGTACCGCA-------------TTTTGTAATT   000021503
sequence2(+)   000008698   ---TAAAGAATGGA--AGGGGACTGAAGACAAGTTGGAGTTTATAAAATC   000008742
sequence3(+)   000008328   ---TAAAGAATGGA--AGGAGACTGAAGACAAGTTGGAGTTTATAAAATC   000008372

sequence1(+)   000021504   ATTAAAGTTTATTGCAAAATT-------GTACACAAATACTAACA-CGAC   000021545
sequence2(+)   000008743   AATGGAATTTATTGCACATCTACTTGGCATTTAATAATTCTCTCATTTCC   000008792
sequence3(+)   000008373   AATGGAATTTATTGCACAGCTACTTGGCATTTAATAATTCTCTCATTTCC   000008422
```

BLAST search results using *shaven/PAX2* conserved sequences

Shown are screenshots of the respective BLAST/BLAT searches.

Drosophila melanogaster >shaven/dPax2 conserved

caaccaattttagttttcagccctccactcaaatacattgaaatgtccatgaagcgggatgtacttttcgcgcatcccaaacatatgtc tcaattgtgtgcactgtcgaaatatccaattgctcatcccgtttcaagctggtactcaaaaatatataaaacctgaaactgttgtctta agggcacaaactaggcagagttaacttctgcccttacaacccccctgtagcatatggtaaccttaacaatctgtaaaa

Results for sv_supercons

**Job name**	sv_supercons
**Species**	Drosophila melanogaster
**Assembly**	BDGP6.22
**Search type**	BLASTN (NCBI BLAST)

Genomic Location	Overlapping Gene(s)	Orientation	Length	Score	E-val	%ID
4:1104898-1105152 [Sequence]	sv	Forward	255 [Sequence]	255	1.6E-142	100.0 [Alignment]
3L:19334323-19334340 [Sequence]	pip	Reverse	18 [Sequence]	18	0.44	100.0 [Alignment]
X:10767960-10767976 [Sequence]	Atg8a	Reverse	17 [Sequence]	17	1.7	100.0 [Alignment]
2R:10373590-10373606 [Sequence]	SLO2	Forward	17 [Sequence]	17	1.7	100.0 [Alignment]
3L:10831824-10831840 [Sequence]		Forward	17 [Sequence]	17	1.7	100.0 [Alignment]
3R:597129-597144 [Sequence]	Myo81F	Reverse	16 [Sequence]	16	6.9	100.0 [Alignment]
3R:6428201-6428216 [Sequence]	gpp	Reverse	16 [Sequence]	16	6.9	100.0 [Alignment]
3R:11410202-11410217 [Sequence]	Irbp	Forward	16 [Sequence]	16	6.9	100.0 [Alignment]
3R:14304543-14304558 [Sequence]	su(Hw)	Forward	16 [Sequence]	16	6.9	100.0 [Alignment]
3R:17418360-17418375 [Sequence]		Reverse	16 [Sequence]	16	6.9	100.0 [Alignment]
3R:19282259-19282274 [Sequence]		Reverse	16 [Sequence]	16	6.9	100.0 [Alignment]
3R:21841190-21841205 [Sequence]		Reverse	16 [Sequence]	16	6.9	100.0 [Alignment]
3R:23185715-23185730 [Sequence]	cnc	Reverse	16 [Sequence]	16	6.9	100.0 [Alignment]
3R:24794564-24794579 [Sequence]	Ets96B	Reverse	16 [Sequence]	16	6.9	100.0 [Alignment]

Mus musculus *>mPax2 conserved*

CAAGCAATTTTAATATTTCTTCAGGGACTTTCCAGCAACAGCACAAGTCATTAATTCACCCTCCCAAATTGCTTATTCAATAGCAGGAA CATGGCTCCTGAATAAAGTCCCAGAATTTATGTGACTTGCACGAGTCAGGAGGTCAAACAACTGTTATGGAGCGAAGTTAAAAATCCAA ATAAAATATTGACACTTTTTTGGGGAGGGGAAGAGAGGAGGCCAAGGTATTTAAAATAGGTTTTTGTTGCAATGCCTCCAGGATAAAGA ATGGAAGGGGACTGAAGACAAGTTGGAGTTTATAAAATCAATGGAATTTATTGCACATCTACTTGGCATTTAATAATTCTCTCATTTC

Results for mPax2 supercons

**Job name**	mPax2 supercons
**Species**	Mouse (Mus musculus)
**Assembly**	GRCm38
**Search type**	BLAT

Genomic Location	Overlapping Gene(s)	Orientation	Query start	Query end	Length	Score	E-val	%ID
19:44778376-44778730 [Sequence]	Pax2, Gm20395	Forward	1	355	355 [Sequence]	687.0	5.4e-198	100.00 [Alignment]

Homo sapiens *>hPAX2 conserved*

CAAGCAATTTTAATATTTCTTCAGGGACTTTCCAGCAACAGCACAAGTCATTAATTCACCCTCCCAAATTGCTTATTCAATAGCAGGAA CATGGCTCCTGAATAAAGTCCCAGAATTTATGTGACTCGCACGAGTCAGGAGGTCAAACAACTGTTATGGAGCGAAGTTAAAAATCCAA ATAAAATATTGACACTTTTTTGGGGAGGGGGAGGTGGGAAGCAAAGGTATTTAAAATAGGTTTTTGTTGCAATGCCCCCAGGATAAAGA ATGGAAGGAGACTGAAGACAAGTTGGAGTTTATAAAATCAATGGAATTTATTGCACAGCTACTTGGCATTTAATAATTCTCTCATTTCC

Results for PAX2 supercons

**Job name**	PAX2 supercons
**Species**	Human (Homo sapiens)
**Assembly**	GRCh38
**Search type**	BLAT

Genomic Location	Overlapping Gene(s)	Orientation	Query start	Query end	Length	Score	E-val	%ID
10:100767632-100767987 [Sequence]	PAX2	Forward	1	356	356 [Sequence]	689.0	1.0e-198	100.00 [Alignment]

Note, for *Drosophila melanogaster*, multiple hits are annotated (in order of appearance in BLAST search); however, only the *shaven/dPax2* conserved CRE matches the cutoff criteria of >62% sequence identity (column %ID) over at least 55 basepairs (column Length) with minimum 1^{e-1} confidence level as the BLAST e-value (column E-val). In the case of human and mouse searches with BLAT instead of BLAST but using the same cutoff criteria, the only CREs identified are those linked to human *PAX2* and mouse *Pax2*.

Inter-species EMBOSS Matcher sequence comparisons related to *shaven-PAX2* CRE
Shown are screen shots of EMBOSS matcher sequence comparisons using genomic regions of random genes of various species that also vary in length and exon-intron structure. Note only *Pax2*-related genomic region (here shown for zebrafish) matches the cutoff criteria for a minimum of 62% sequence identity over a length of at least 55 basepairs.

***Danio rerio Pax2a* genomic region against *Drosophila shaven_VT51937* CRE**

```
#=======================================
#
# Aligned_sequences: 2
# 1: DrPax2
# 2: shaven_VT51937
# Matrix: EDNAFULL
# Gap_penalty: 16
# Extend_penalty: 4
#
# Length: 91
# Identity:      59/91 (64.8%)
# Similarity:    59/91 (64.8%)
# Gaps:           7/91 ( 7.7%)
# Score: 95
#
#
#=======================================

DrPax2            42 GCTTTTTAAGTTCATATAGATGGTTTGTGAAATTGTTTTAT-TATTATTA     90
                     |||||||.|.||..||.| |.|..|| |.|..||.|.|.|. |.||||||
shaven_VT5193   1339 GCTTTTTTACTTGTTACA-ACGAATT-TTACTTTCTATAACATTTTATTA   1386

DrPax2            91 T--TATTATTATTAATTTATTGTATT-TAATTTTAC-ATTA    127
                     |  |||||.|||.||.|.||..||.. ||||.|||| ||||
shaven_VT5193   1387 TCGTATTAGTATAAAATAATAATACGATAATATTACGATTA   1427
```

***Pan troglodytes APP* genomic region against *Drosophila shaven_VT51937* CRE**

```
#=======================================
#
# Aligned_sequences: 2
# 1: PtAPP
# 2: shaven_VT51937
# Matrix: EDNAFULL
# Gap_penalty: 16
# Extend_penalty: 4
#
# Length: 624
# Identity:     338/624 (54.2%)
# Similarity:   338/624 (54.2%)
# Gaps:          78/624 (12.5%)
# Score: 186
#
#
#=======================================

PtAPP         163863 AATTGTATGTTGTATTATATAATTATATATTGTAATA-TATAGTATA-TA 163910
                     ||||.||..||.|||.|.||. |||| |||.|||.|| ||||..||| ||
shaven_VT5193   1360 AATTTTACTTTCTATAACATT-TTAT-TATCGTATTAGTATAAAATAATA   1407

PtAPP         163911 ATAT-ATAAT-TTATAAATATAATATATAAATTATATTTATAAA----TT 163954
                     |||. ||||| |||..|.||..||...||.  .||.||||.|||    ||
shaven_VT5193   1408 ATACGATAATATTACGATTACGATTAGTAC--GATCTTTAAAAACTTATT   1455

PtAPP         163955 ATAAAGTTTATATATAATATATTATAATCTAATATAA----TTATATTTA 164000
                     |.|.|||||..||.|| ||||||.|.|.|..|.|...    |.|.|.|||
shaven_VT5193   1456 ACATAGTTTTGATTTA-TATATTTTGACCACAAAAGGCCTGTAAGACTTA   1504

PtAPP         164001 TAA-----ATTATAACGTTTATTTGAAATATTGAAGTTTTGTATACTTAG 164045
                     ..|     |..|.|||.||.|.|..||.||.||||||.    |||.||||
shaven_VT5193   1505 GTAGAGCCACCAAAACTTTAAGTATAAGTACTGAAGTC----ATAATTAG   1550

PtAPP         164046 TGGATTTTAATACTTATGTCGTTTTCATTTTATTCCACTGTTACATTAAG 164095
                     .|..|.|.||||...|||.    ||.|..|...|.|.||..|...|....
shaven_VT5193   1551 AGATTATAAATAACAATGA----TTTACCTACCTACCCTCATGGGTAGGT   1596

PtAPP         164096 GAAGTAACTTACTACAAAAAT-AAATTATATTTGTCTTACATTTG----- 164139
                     ||..|.|..||.||..||..| |||.|.|...||.||.||.|..|
shaven_VT5193   1597 GATTTTAAGTAATATGAACGTTAAAGTGTTCATGGCTAACCTGGGGAACA   1646

PtAPP         164140 --CCCTTTTTACTCTACTTAAAATTGGAGAAAAAGAGTTCAAG------- 164180
                       |.|||.||......||..|..||......||..|||.||||
shaven_VT5193   1647 GTCACTTCTTGGATATCTCCATCTTCCTTGCAAGAAGTGCAAGGGGAACC   1696

PtAPP         164181 ATAGGGGATTT--CTGTCAGAAAGGAGAAATAGTTGTATTATGTGAAGTT 164228
                     ||||.||..|.  ||.|...|||....|.||...||||..|......|||
shaven_VT5193   1697 ATAGTGGTCTATCCTCTTTTAAATATTATATGTATGTACAACTGATTGTT   1746

PtAPP         164229 TAA-GTAGATATTCTTGTAGAGTATGGA---GAAAATAATGCTATAAAAT 164274
                     ||. ||||.||.|..||||.|  ||.||   |...||||...|.|||||.
shaven_VT5193   1747 TATTGTAGTTAGTTGTGTACA--ATTGATTTGTTGATAAGTATGTAAAAA   1794

PtAPP         164275 GTTGCCTTTTAAAAAAAAGTTCAGAA--AATATAGTTCCTAGC-TCTCTC 164321
                     ||.|      ||.||||.||||.||.  ||||.|||||.||.. |||...
shaven_VT5193   1795 GTAG------AAGAAAACGTTCTGACCCAATAAAGTTCTTATAATCTTGG   1838

PtAPP         164322 TAA--ATGACTAGTT---TGATTGTGACTGCTTTCCCAAAATTTGTAAAT 164366
                     |.|  ||.|||.|..   ||||||||.|  |.|..||||||.|..|||||
shaven_VT5193   1839 TTAGGATCACTTGCCGAGTGATTGTGGC--CATGTCCAAAACTGTTAAAT   1886

PtAPP         164367 TTAAAATTTATACATCACATGGAATATAGTTTTATTTCACATACATTCCA 164416
                     .||.||||..|...|.||.|. |.||.|....||||||..|| |.|||
shaven_VT5193   1887 ATATAATTCTTTTCTAACTTT-ATTACAAGACTATTTCTGAT-CCTTC--   1932

PtAPP         164417 GTAGGCTGAGAATAC-TTGAAATT 164439
                         |||.|.||.|. ||||||||
shaven_VT5193   1933 ----GCTAACAAAATGTTGAAATT   1952
```

Mus musculus Pax5 **genomic region against** ***Drosophila shaven_VT51937*** **CRE**

```
#=======================================
#
# Aligned_sequences: 2
# 1: mPax5
# 2: shaven_VT51937
# Matrix: EDNAFULL
# Gap_penalty: 16
# Extend_penalty: 4
#
# Length: 131
# Identity:      75/131 (57.3%)
# Similarity:    75/131 (57.3%)
# Gaps:           1/131 ( 0.8%)
# Score: 139
#
#
#=======================================

mPax5         126933 TTGTGAAAACACGTTTTACTTTCAAAAGCATTTTATTTATCTCATAGCAC 126982
                     ||||.|.|||...||||||||||.|.|.|||||||||....|..|||.|.
shaven_VT5193   1349 TTGTTACAACGAATTTTACTTTCTATAACATTTTATTATCGTATTAGTAT   1398

mPax5         126983 ATGAATCTTATCAATTGTTTTAACTAGTAGAAACAAGACTATATAGTATG 127032
                     |..|...|.||....|..|.|.||.|.||..|..|..||.||.|. ||..
shaven_VT5193   1399 AAAATAATAATACGATAATATTACGATTACGATTAGTACGATCTT-TAAA   1447

mPax5         127033 GAATTCTCTTTTTATTTTGTTTAATACATTT 127063
                     .|.||.|....|..|||||.||.|||.||||
shaven_VT5193   1448 AACTTATTACATAGTTTTGATTTATATATTT   1478
```

Homo spaiens HEY1 **genomic region against** ***Drosophila shaven_VT51937*** **CRE**

```
#=======================================
#
# Aligned_sequences: 2
# 1: hHEY1
# 2: shaven_VT51937
# Matrix: EDNAFULL
# Gap_penalty: 16
# Extend_penalty: 4
#
# Length: 111
# Identity:      65/111 (58.6%)
# Similarity:    65/111 (58.6%)
# Gaps:           7/111 ( 6.3%)
# Score: 93
#
#
#=======================================

hHEY1           2732 AACATCACCTTAAAGTCGTCAGTA-AAAGTAAAAAG--GAAAAAGGTACA   2778
                     |||||....|||..||. |.|||| |||.|||.||.  ||.||...|||.
shaven_VT5193   1375 AACATTTTATTATCGTA-TTAGTATAAAATAATAATACGATAATATTACG   1423

hHEY1           2779 CTTTCAGATAATTTTTTTTTTAAAGACTAAAGGTTTGTTGGTTTACTTTT   2828
                     .||.|...||.|....|.||||||.|||.|   ||...|.||||...|||
shaven_VT5193   1424 ATTACGATTAGTACGATCTTTAAAAACTTA---TTACATAGTTTTGATTT   1470

hHEY1           2829 ATCTTTTTTAA   2839
                     ||.|.||||.|
shaven_VT5193   1471 ATATATTTTGA   1481
```

Gallus gallus Fgf8 **genomic region against** ***Drosophila shaven_VT51937*** **CRE**

```
#=======================================
#
# Aligned_sequences: 2
# 1: gFgf8
# 2: shaven_VT51937
# Matrix: EDNAFULL
# Gap_penalty: 16
# Extend_penalty: 4
#
# Length: 50
# Identity:      32/50 (64.0%)
# Similarity:    32/50 (64.0%)
# Gaps:           0/50 ( 0.0%)
# Score: 88
#
#
#=======================================

gFgf8           3204 TTTTCAGATGTAAAGTTTTAGGAGTTGCAAGTCTGTAAATGTTAAAATAG   3253
                     |.||.|.|||||||.|||..|.|.||..|...|||.||.|...||||||
shaven_VT5193    182 TGTTTAAATGTAAATTTTATGAAATTTTAAAAATGTTAAAGCCCAAATAG    231
```

Petromyzon marinus **(lamprey)** ***Wnt1*** **genomic region against** ***Drosophila shaven_VT51937*** **CRE**

```
#=======================================
#
# Aligned_sequences: 2
# 1: lWnt1
# 2: shaven_VT51937
# Matrix: EDNAFULL
# Gap_penalty: 16
# Extend_penalty: 4
#
# Length: 30
# Identity:      23/30 (76.7%)
# Similarity:    23/30 (76.7%)
# Gaps:           0/30 ( 0.0%)
# Score: 87
#
#
#=======================================

lWnt1            856 ACGAACAGCACTTTCCCTAACAGTTTATTA    885
                     |||||....||||||..|||||.|||||||
shaven_VT5193   1357 ACGAATTTTACTTTCTATAACATTTTATTA   1386
```

***D. melanogaster shaven* gene locus and homology to other insects**

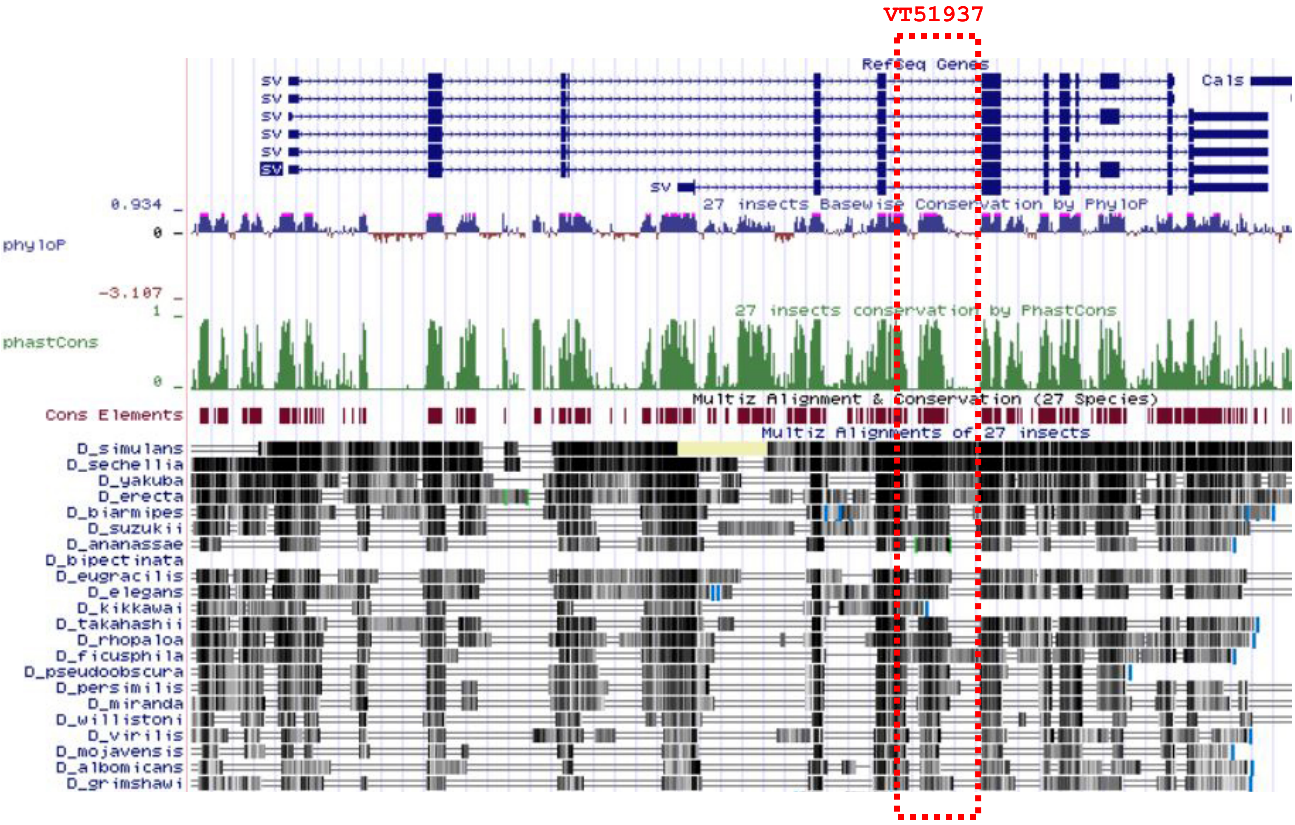

Image capture of UCSC Genome Browser on *D. melanogaster* Aug. 2014 (BDGP Release 6 + ISO1 MT/dm6) assembly, shown is the *shaven* gene locus. RefSeq Genes shows exon (blue bars) and intron structure; in red is shown Ensembl Gene annotation used for genome browsing. Green bar diagram indicates conservation among *Drosophilidae* species which are listed further below (black bars indicate sequence homology). Conserved elements are shown in dark red. Dashed red box indicates topology and extension of VT51937 CRE sequence determined in *Drosophila melanogaster*.

Examples of sv/Pax2 conserved sequence found in other *Drosophilidae*

D simulans scaffold

>chromosome:GCA_000259055.1:4:863940:864789:1

**CAACCAATTTTAGTTTTCAGCCCTCCACTCAAATACATTGAAATGTCCATGAAGCGGGATGTACTTTTCTCGCATCCCAA
ACATATGTCTCAATTGTGTGCACTGTCCAAATATCCAATTGCTCATCCCGTTTCAAGCTGGTACTCAAAAAAACATAAAA
CCTGAAACTGTTGTCTTCAGGGCACAAACTAGGCAGAGTTAACTTCTGCCCTTACAACCCCCTGTAGCATATGGTTACCT
TAACAATCTG**

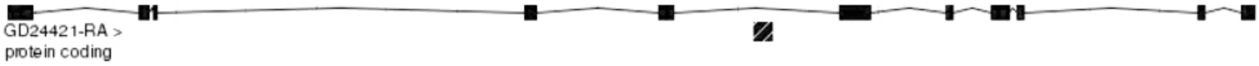

D sechellia scaffold

>supercontig:GCA_000005215.1:scaffold_40:169522:170371:1

**CAACCAATTTTAGTTTTCAGCCCTCCACTCAAATACATTGAAATGTCCATGAAGCGGGATGTACTTTTCTCGCATCCCAA
ACATATGTCTCAATTGTGTGCACTGTCCAAATATTCAATTGCTCATCCCGTTTCAAGCTGGTACTCAAAAAAACATAAAA
CCTGAAACTGTTGTCTTCAGGGCACAAACTAGGCAGAGTTAACTTCTGCCCTTACAACCCCCTGTAGCATATGGTTACCT
TAACAATCTG**

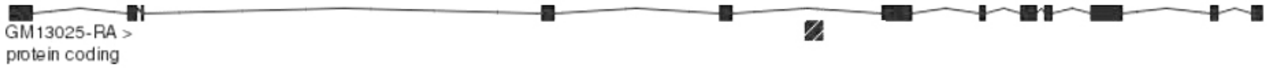

D yakuba scaffold

>chromosome:GCA_000005975.1:4:1205049:1205838:1

CAACCAATTTTAGTTTTCAGCCCTCCACTCAAATACATTGAAATGTGCATGT**GCGGGATGTACTTTTCTCGCATCCCAAA
CATATGTCTCAATTGTGTGCACTGTCCATATATCCAATTGCTCATCCCGTTTCAAGCTAGTACTCAAAAAAAAAGAAAAC**

CTAAAACTGTTGTCTTCAGGACACAAACTGGGCAGAATTAACTTCTGCCCTTACAACCCTCTGTAGCATATGGTTACCTTAA

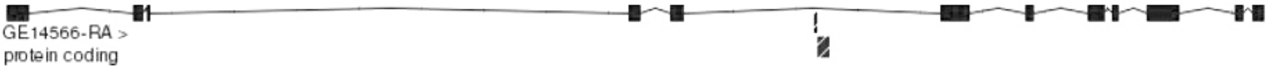

D pseudoobscura scaffold

>supercontig:GCA_000001765.2:Unknown_group_58:6806:7477:-1
TGTACTTTACTCACATCCCAAACATATGTCTCAATTGTGTGCACTGCCCAAATACCCAATTGCTCATCCCGTCTCTGAGCCGGCGCTTGCAGGAAAGAATCGAAAATCTGAAACTGATGTCAGCTTCCATCAT**TTGGACTGAATGGGCTTCTCTTCTT**

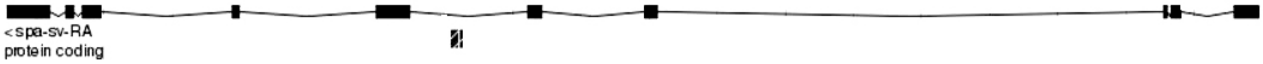

D ananassae

AACATATGTCTCAATTGTGTGCACTGCCCAAATATCAAATTGC

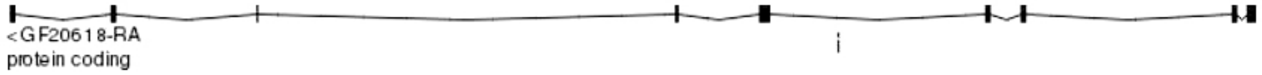

D willistoni

>supercontig:GCA_000005925.1:scf2_1100000004943:1805136:1805769:-1
CGCATCCCAAACATATGTTTCAATTGTGTTCACT

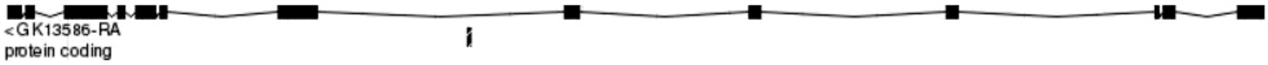

Shaven-PAX2 conserved CRE - potential transcription factor binding sites

Shaven/dPax2, *mDPax2* and *hPAX2* conserved CRE sequence alignment was generated using MLAGAN (http://genome.lbl.gov/vista/lagan/submit.shtml). Potential transcription factor binding sites were identified using JASPAR (http://jaspar.genereg.net/); potential binding sites are color coded as per legend below.

Seq1 = shaven supercons; Seq2 = mPax2 supercons; Seq3 – hPAX2 supercon

```
sequence1(+)    000000001  CAACCAATTTTAG---------------TTTTCAG---------------  000000020
sequence2(+)    000000001  CAAGCAATTTTAATATTTCTTCAGGGACTTTCCAGCAACAGCACAAGTCA  000000050
sequence3(+)    000000001  CAAGCAATTTTAATATTTCTTCAGGGACTTTCCAGCAACAGCACAAGTCA  000000050

sequence1(+)    000000021  --------CCCTCC----------ACTCAAATACATTGAAATGTC-CATG  000000051
sequence2(+)    000000051  TTAATTCACCCTCCCAAATTGCTTATTCAATAGCAGGAACATGGCTCCTG  000000100
sequence3(+)    000000051  TTAATTCACCCTCCCAAATTGCTTATTCAATAGCAGGAACATGGCTCCTG  000000100

sequence1(+)    000000052  AAGCGGG-----------ATGTACTTTTCGCGCATC------CCAAACAT  000000084
sequence2(+)    000000101  AATAAAGTCCCAGAATTTATGTGACTTGCACGAGTCAGGAGGTCAAACAA  000000150
sequence3(+)    000000101  AATAAAGTCCCAGAATTTATGTGACTCGCACGAGTCAGGAGGTCAAACAA  000000150

sequence1(+)    000000085  ATGTCTCAATTGTGTGCACT-----GTCGAAATATCCAATTGCTCATCCC  000000129
sequence2(+)    000000151  CTGTT---ATGGAGCGAAGTTAAAAATCCAAATA---AAATATTGACACT  000000194
sequence3(+)    000000151  CTGTT---ATGGAGCGAAGTTAAAAATCCAAATA---AAATATTGACACT  000000194

sequence1(+)    000000130  GTTTC---------AAGCTGGTACTCAAAAATATATAAAACCTGAAACTG  000000170
sequence2(+)    000000195  TTTTTGGGGAGGGGAAGAGAGGAGGCCAAGGTATTTAAAATAGGTTTTTG  000000244
sequence3(+)    000000195  TTTTTGGGGAGGGGGAGGTGGGAAGCAAAGGTATTTAAAATAGGTTTTTG  000000244

sequence1(+)    000000171  TTGTCTT--------------------AAGGGCAC---AAACTAGGCAGA  000000197
sequence2(+)    000000245  TTGCAATGCCTCCAGGATAAAGAATGGAAGGGGACTGAAGACAAGTTGGA  000000294
sequence3(+)    000000245  TTGCAATGCCCCCAGGATAAAGAATGGAAGGAGACTGAAGACAAGTTGGA  000000294

sequence1(+)    000000198  GTT--------------AACTTCTGCCCTTACAACCCCCTGTAGCATATG  000000233
sequence2(+)    000000295  GTTTATAAAATCAATGGAATTTATTGC---ACATCTACTTG--GCATTTA  000000339
sequence3(+)    000000295  GTTTATAAAATCAATGGAATTTATTGC---ACAGCTACTTG--GCATTTA  000000339

sequence1(+)    000000234  GTAACCTTAACAATCTGTAAAA  000000255
sequence2(+)    000000340  ATAATTCTCTCATTT------C  000000355
sequence3(+)    000000340  ATAATTCTCTCATTTC-----C  000000356
```

ACTCAAATA = *ventral nervous system defective (vnd)*
CAATTGC = *Distalless (Dll)*
TCATCC = *ocelliless/orthodenticle (oc/otd)*
AAATAT = *engrailed (en)*
TGGTAC = *sine occulis (so)*
TGAAAC = *so*
TGAAACTG = *Paired (Prd)*

SUPPLEMENTARY DATA SET S2

invected/engrailed - EN2 conserved CRE sequence

Fly inv/en conserved

```
Location: BDGP6:2R:11516158:11517373:1
TGAAAACTGAAGACTGCAACCAGGGACCACACACGACGATCGAGGCTGTGCCACAAGCTGCTCTGGGCAACGGAAGGCGGCAACATCGA
CAGTAGTTTTGCGGCTCGTTTGCTGCGTTGACTCTTCGCCGAGTCCCGTGATCATGGACATGATCATCATGACGTACGATGATCGTGGG
GTCCGCAGAACGTCATCATCGCGAAGATCTTCCTCCTCCTGATGCGGCGCCAAATTGCAGCAAAGTAAAACAGCGTTTAGCATTGTGCT
TGTAATGATCTTGGTCAGGCAATCCGAGGGCCTGGCGTCCACAAATCTACAGAAGAACCCAAGTCTGGAGATTCAAAGAGAGGTCCCCG
AGATTCTGGGCGACTTAAGAGAAGAACAGCGCAACTCGTTCGCTGAGTTAGCGCCTTTGCAATGGGTGTAATAATTTATGTATTTTGAT
TAGGCCGAAGTTTCTGTGACTGTGGCTCGGCTTTTGCCTTTTAATTGATAAGTGTGTTTGAGATACTTTTTAATGAAGCTAATGAAACA
TGGTGAGCTTTCAGATATGTCTATATCTTTCAAGGGTTTAGGCACTCCGGTAATTTAATAAACATTTAAATCTTTAAATGAA
```

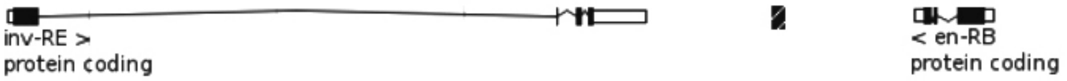

Human EN2 conserved

```
Location: Chr7 - 155471753 to 155472126 (+)
TCAAACTTGAATATGCAATGAGACGCGGGAGCTTTGTGGCGGAAATATAATTAAACTGGGGAAAGATGTAAAAGCTGAAGAATGGGATG
ACATCAGGCTGATTTCACACTCGGCAGTCGGATCGCTGGGCCCAAGCCGCGCTCTTGCCACGCAAGGCAGATCAAAGTGCCCTGCCACC
GCTAAAAAGGCAAAAGGGGACTTAAGTATGCTAATCCCCAGGACAAATATATTTTAATCTTGTTAGAATACAAGTTAATGCTGCAGCTC
AGTGGCTGAACTTGGTCAGACTGTAGAGATCCATTTTTATTTTAGCTATGAATGAGCTAGACCTCTTGGTTTATTCATCGGTAATAAAA
GTAAATTTACAAAACGAA
```

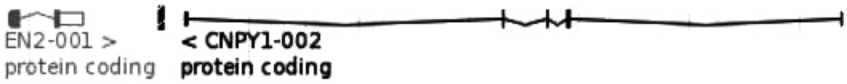

Mouse En2 conserved

```
Location: GRCm38:5:28178990:28179962:1
TCAAACTTGCATATGCAACGAGGTGCTGGAGCTTTGTGGCGGAAATATAATTAAACTGGTGAGAGATGTAAAAGATGAAGAATGGGATG
ACATCAGACTGATTTCACACTTGGCAGTCCGATAGCTGGGCCCAAGCCGGGCTTGCCACGCAAGGCAGATCAAAGTGCTCTGCCACCAC
TAAAAAGGCAAAGGGAGACTTAAGTATGCTAATCCCCAGGACAAATATATTTTAATCTTGTTAGAATACAAGTTAATGCTGCAGTGCAG
TGGCTGAACTTGGTCAGGCAGGAGAGACCCATTTTTATCTTAGCTATGAATGAGGTGAAGCTCTTGGTTTTATTCATCAGTAATAAAAG
TACATTTATAAAACGAA
```

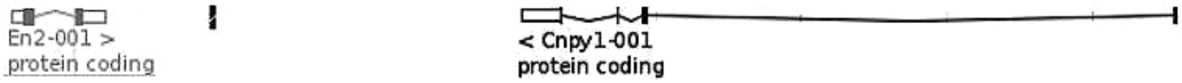

Chicken En

```
Location: Gallus_gallus-5.0:2:7916330:7917255:1
TCAAACTTGAATATGCAATGAAGCTCCAGAGCTTTGTGGCGGAAATATAATTAAACTGGTGAAAGATGTAAAAGATGAAGAATGGGGAT
GACATCAGGCCGATTTCACACTTGGCACTCGGATGGCTGGGCCCAAGCCGTGCTCTTGCCACGCAACACAGATCAAAGTGCACTGCCAC
TGCTAAAAAGGCAAAAGGGGACTTAAGTATGCTAATCCCCAGGACAAATATATTTTAATCTTGTTAGAATACAAGTTAATGCTGCAGCT
CAGTGACTGAACTTTGTCAGAGTGTAGAGATCCACTTTTACTTTAGCTATGAATGAGGT
```

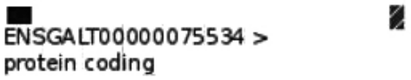

Zebrafish eng2b

```
Location: GRCz10:2:30000078:30000793:1
TCAAACTTGAATATGCAATGAAGAGCTGGGGCTTTGTGGCGGAAATATAATTAAGGCAGTGAAAGATGTAGAGGGTGAAGAATGGGGAT
GACATCAGGCCAATTTCACACTTGGCA
Location: GRCz10:2:30000221:30000844:1
CTTGCCACGCAACACAGATCAAA
Location: GRCz10:2:30000277:30000935:1
ATTTAAGTATGCTAATCTCCAGGACAAATATATTTTAATCTTGTTAGAATACAAGTTAA
```

Scaffold:

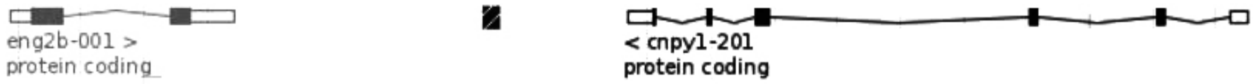

Fugu eng2b

Location: FUGU4:scaffold_142:748369:749065:1

GAGGTGCCCGGGCTTTGTGGCGGAAACATAATTAAAGTGGTGAAAGATGTAAAAGGTGAAGAATGGGGATGACATCAGGCCAATTTCAC
ACTTGGCA

Location: FUGU4:scaffold_142:748570:749245:1

TATGCTAATCCCCAGGACGAATATATTTTAATCTTGTTAGAATACAAGTTAATGCCGTGGCTCAGTGGCTGAACTT

Scaffold:

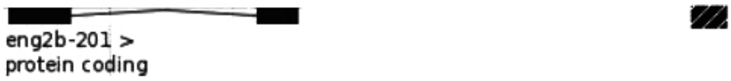

>Human|chr7:155264047-155265809 | element 1418 | positive | hindbrain (rhombencephalon)[4/5] | midbrain (mesencephalon)[4/5]

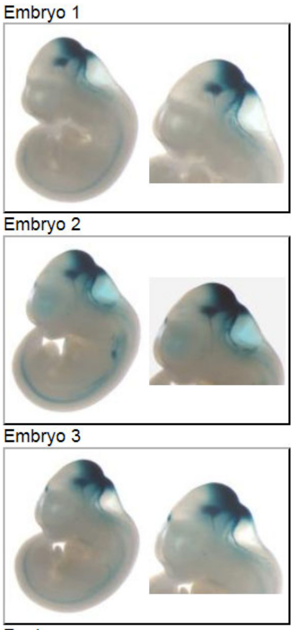

CAGTGACTCATCACTCGCAGAgagaaaaacatcttttatatgtagagacgggaacgcgccctgtggattcagagccccaaggaaacaaa
aacagcacgtcagaggaagaatgtcatacaaacccgctggagtcacaaggcaaaacagcccccatacctaaacgcccggcgtgacaatg
gcctttgtgcctggcgccgtcgggggagctgggcgcctagcgggtggggcaggcggaagttggacacatttccacgcaggtgaggcca
tccatggggtgatgcgggtgttggggctcgctgctttcgctccatgaagatttgcagcccccccagccaacccaccccatcccttccc
cccacaagggtatttcatccacactgtttttaaatgcttcctagtcaaacttgaatatgcaatgagacgcgggagctttgtggcggaa
atataattaaactggggaaagatgtaaaagctgaagaatgggatgacatcaggctgatttcacactcggcagtcggatcgctgggccca
agccgcgctcttgccacgcaaggcagatcaaagtgccctgccaccgctaaaaaggcaaaaggggacttaagtatgctaatccccaggac
aaatatattttaatcttgttagaatacaagttaatgctgcagctcagtggctgaacttggtcagactgtagagatccattttttattta
gctatgaatgagctagacctcttggtttattcatcggtaataaaagtaaatttacaaaacgaacccctctttgggctgtgtcaccccca
ccttgagctctccttactggggactggggaattccagaggcttccaagatgggagcatccctaacaccatccatccttctccgacctgt
ccccttctgtagagcatcctgatggccgaggaaggaactctgctcacgcagcagagaagaaacagggaaactcaaaaattaattaaaag
attctccaaagcaatctgtttccctttaggcttgtggtttgacaaccaaaccaattcctttctgtcagccctctcactttaatttttgc
aatgtcaattttcattactattaattatttgccaagctccaactgtacatttggcattgctctgatgagcaagaagctctcaggttcct
ccctctttaagtgtcgattcctggaagatatgaatatgcaagtttctgcgtgtgtgtacatgtaaaatattgggcacacatagaagaag
aaatgtcagcctgtagagctccccttgaaataaaaccactgtggtaaaatattaagcagaaccacggatatatctgcacagaggaatgt
gtgtgtgtgtgtgtgtgtgtgtgagagagagagagagagagagagagagagagagagagagcgagccagccagcgagccagcacttaac
tgtaaggttacagaaggttaaacggccctgcagagcctgcctgctaaatattgctgatggcagccgcgtctaaaaggcaatttctgtct
ttctgctctagcgtgtgttagaattccctctgcctcaggcagaaaacctgactcccactaaagtgtgctgcacagccctgcattccaaa
gcctgtctgtgcccttccggctccctggcctcagcagggggggcgggtgcaggaggctatgcgcgcacacacagtcccccaggcaggcc
aggcaactggcagcccgaggggaggggcagctggcagcagtatccccctcccCCACAGAGAGCTTGGAAGGT

Left, intergenic sequence of hs1418 enhancer element, conserved sequence highlighted in red. Right, three examples of hs1418 driven lacZ expression in mouse E11.5 brain specific to MHB (VISTA database, with permission; data from ref. 54).

EMBOSS MATCHER human EN2 consensus *inv/en* conserved with hs1418

```
# Length: 374 - # Identity: 374/374 (100.0%) - # Similarity: 374/374 (100.0%)
# Gaps: 0/374 ( 0.0%) - # Score: 1870

EMBOSS_001         1 TCAAACTTGAATATGCAATGAGACGCGGGAGCTTTGTGGCGGAAATATAA     50
                     ||||||||||||||||||||||||||||||||||||||||||||||||||
EMBOSS_001       402 TCAAACTTGAATATGCAATGAGACGCGGGAGCTTTGTGGCGGAAATATAA    451

EMBOSS_001        51 TTAAACTGGGGAAAGATGTAAAAGCTGAAGAATGGGATGACATCAGGCTG    100
                     ||||||||||||||||||||||||||||||||||||||||||||||||||
EMBOSS_001       452 TTAAACTGGGGAAAGATGTAAAAGCTGAAGAATGGGATGACATCAGGCTG    501

EMBOSS_001       101 ATTTCACACTCGGCAGTCGGATCGCTGGGCCCAAGCCGCGCTCTTGCCAC    150
                     ||||||||||||||||||||||||||||||||||||||||||||||||||
EMBOSS_001       502 ATTTCACACTCGGCAGTCGGATCGCTGGGCCCAAGCCGCGCTCTTGCCAC    551

EMBOSS_001       151 GCAAGGCAGATCAAAGTGCCCTGCCACCGCTAAAAAGGCAAAAGGGGACT    200
                     ||||||||||||||||||||||||||||||||||||||||||||||||||
EMBOSS_001       552 GCAAGGCAGATCAAAGTGCCCTGCCACCGCTAAAAAGGCAAAAGGGGACT    601

EMBOSS_001       201 TAAGTATGCTAATCCCCAGGACAAATATATTTTAATCTTGTTAGAATACA    250
                     ||||||||||||||||||||||||||||||||||||||||||||||||||
EMBOSS_001       602 TAAGTATGCTAATCCCCAGGACAAATATATTTTAATCTTGTTAGAATACA    651

EMBOSS_001       251 AGTTAATGCTGCAGCTCAGTGGCTGAACTTGGTCAGACTGTAGAGATCCA    300
                     ||||||||||||||||||||||||||||||||||||||||||||||||||
EMBOSS_001       652 AGTTAATGCTGCAGCTCAGTGGCTGAACTTGGTCAGACTGTAGAGATCCA    701

EMBOSS_001       301 TTTTTATTTTAGCTATGAATGAGCTAGACCTCTTGGTTTATTCATCGGTA    350
                     ||||||||||||||||||||||||||||||||||||||||||||||||||
EMBOSS_001       702 TTTTTATTTTAGCTATGAATGAGCTAGACCTCTTGGTTTATTCATCGGTA    751

EMBOSS_001       351 ATAAAAGTAAATTTACAAAACGAA    374
                     ||||||||||||||||||||||||
EMBOSS_001       752 ATAAAAGTAAATTTACAAAACGAA    775
```

MLAGAN_*inv/en-EN2*_conserved_CNS

```
sequence1(+)    000006950  ACT------GCAGACTGCAGACTGAAAACTGAAGACTGCAACCAGGGACC  000006993
sequence2(+)    000007120  TTTTAAAAGAAATGCTTCCTGGTCAAACTTGCATA-TGCAACGAGG----  000007164
sequence3(+)    000007093  GTTT--TTTAAATGCTTCCTAGTCAAACTTGAATA-TGCAATGAGA----  000007135

sequence1(+)    000006994  ACACACGACGATCGAGGCTGTGCCACAAGCTGCTCTGGGCAACG------  000007037
sequence2(+)    000007165  -TGCTGGAGCTTTGTGGCGGAAATATAA-TTAAACTGGTGAGAGATGTAA  000007212
sequence3(+)    000007136  -CGCGGGAGCTTTGTGGCGGAAATATAA-TTAAACTGGGGAAAGATGTAA  000007183

sequence1(+)    000007038  --------GAAGGCGGCAACATCGACAGTAGTTTTGCGGCTCGTTTGCTG  000007079
sequence2(+)    000007213  AAGATGAAGAATGGGATGACATCAG---------------------ACTG  000007241
sequence3(+)    000007184  AAGCTGAAGAATGGGATGACATCAG---------------------GCTG  000007212

sequence1(+)    000007080  CGTTGACTCTTCGCCGAGTCCCGTGATCATGGACATGATCATCATGACGT  000007129
sequence2(+)    000007242  ATTTCACACTTGGC--AGTCC-----------------------------  000007260
sequence3(+)    000007213  ATTTCACACTCGGC--AGTCG-----------------------------  000007231

sequence1(+)    000007130  ACGATGATCGTGGGGTCCGCAGAACGTCATCATCGCGAAGATCTTCCTCC  000007179
sequence2(+)    000007261  -----GATAGCTGGGCCCAAGCCGGG----------------CTT-----  000007284
sequence3(+)    000007232  -----GATCGCTGGGCCCAAGCCGCGCT--------------CTT-----  000007257

sequence1(+)    000007180  TCCTGATGCGGCGCCAAATTGCAGCAAAGTAAAACAGCGTTTAGCATTGT  000007229
sequence2(+)    000007285  -----------------------GCCACGCAAGGCAG-------------  000007298
sequence3(+)    000007258  -----------------------GCCACGCAAGGCAG-------------  000007271

sequence1(+)    000007230  GCTTGTAATGATCTTGGTCAGGCAATCCGAGGGCCTGGCGTCCAC---AA  000007276
sequence2(+)    000007299  ------------------------ATCAAAGTGCTCTGCCACCACTAAAA  000007324
sequence3(+)    000007272  ------------------------ATCAAAGTGCCCTGCCACCGCTAAAA  000007297

sequence1(+)    000007277  ATCTACAGAAGAACCCAAGTCTGGAGATTCAAAGAGAGGTCCCCGAGATT  000007326
sequence2(+)    000007325  AGGCAAAGGGAGACTTAAGTATGCTAATC---------------------  000007353
sequence3(+)    000007298  AGGCAAAAGGGGACTTAAGTATGCTAATC---------------------  000007326
```

```
sequence1(+)    000007327  CTGGGCGACTTAAGAGAAGAACAGCGCAA-CTCGTTCG---CTGAGTTAG  000007372
sequence2(+)    000007354  --------CCCAGGACAAATATATTTTAATCTTGTTAGAATACAAGTTAA  000007395
sequence3(+)    000007327  --------CCCAGGACAAATATATTTTAATCTTGTTAGAATACAAGTTAA  000007368

sequence1(+)    000007373  CGC--CTTTGCAATGGGTGTAATAATTTATGTATTTTGATTAGGCCGAAG  000007420
sequence2(+)    000007396  TGCTGCAGTGCAGTGGCTG-------------AACTTGGTCAGGCAGGA-  000007431
sequence3(+)    000007369  TGCTGCAGCTCAGTGGCTG-------------AACTTGGTCAGACTGTA-  000007404

sequence1(+)    000007421  TTTCTGTGACTGTGGCTCGGCTTTTGCCTTTTAATTGATAAGTGTGTTTG  000007470
sequence2(+)    000007432  -------------------------------------------------G  000007432
sequence3(+)    000007405  -------------------------------------------------G  000007405

sequence1(+)    000007471  AGATAC-TTTTTAATGAAGCTAATGAAACATGGTGAGCTTTCAGATATGT  000007519
sequence2(+)    000007433  AGACCCATTTTTATCTTAGCT-ATGAATGAGGTGAAGCTCTTGGTTTTAT  000007481
sequence3(+)    000007406  AGATCCATTTTTATTTTAGCT-ATGAATGAGCTAGACCTCTTGG-TTTAT  000007453

sequence1(+)    000007520  CTATATCTTTCAAGGGTTTAGGCACTCCGGTAATTTAATAAACATTTAAA  000007569
sequence2(+)    000007482  TCAT-----------------------CAGTAA-TAAAAGTACATTTATA  000007507
sequence3(+)    000007454  TCAT-----------------------CGGTAA-TAAAAGTAAATTTACA  000007479

sequence1(+)    000007570  TCTTTAAATGAATTTACTATGGCGATACGT------TGGCTTGACATCTT  000007613
sequence2(+)    000007508  -----AAACGAACCTCTCTTTGGACTGTGTCACCCCCACCTTAAGCTTCC  000007552
sequence3(+)    000007480  -----AAACGAACCCCTCTTTGGGCTGTGTCACCCCCACCTTGAGCTCTC  000007524
```

BLAST search results using *invected/engrailed - EN2* conserved sequence

Shown are screenshots of the respective BLAST searches.

Drosophila melanogaster >inv/en conserved

TGAAAACTGAAGACTGCAACCAGGGACCACACACGACGATCGAGGCTGTGCCACAAGCTGCTCTGGGCAACGGAAGGCGGCAACATCGA CAGTAGTTTTGCGGCTCGTTTGCTGCGTTGACTCTTCGCCGAGTCCCGTGATCATGGACATGATCATCATGACGTACGATGATCGTGGG GTCCGCAGAACGTCATCATCGCGAAGATCTTCCTCCTCCTGATGCGGCGCCAAATTGCAGCAAAGTAAAACAGCGTTTAGCATTGTGCT TGTAATGATCTTGGTCAGGCAATCCGAGGGCCTGGCGTCCACAAATCTACAGAAGAACCCAAGTCTGGAGATTCAAAGAGAGGTCCCCG AGATTCTGGGCGACTTAAGAGAAGAACAGCGCAACTCGTTCGCTGAGTTAGCGCCTTTGCAATGGGTGTAATAATTTATGTATTTTGAT TAGGCCGAAGTTTCTGTGACTGTGGCTCGGCTTTTGCCTTTTAATTGATAAGTGTGTTTGAGATACTTTTTAATGAAGCTAATGAAACA TGGTGAGCTTTCAGATATGTCTATATCTTTCAAGGGTTTAGGCACTCCGGTAATTTAATAAACATTTAAATCTTTAAATGAA

Results for inv/en_supercons

Job name	inv/en_supercons
Species	Drosophila melanogaster
Assembly	BDGP6.22
Search type	BLASTN (NCBI BLAST)

Genomic Location	Overlapping Gene(s)	Orientation	Length	Score	E-val	%ID
2R:11516458-11517073 [Sequence]		Forward	616 [Sequence]	616	0.0	100.0 [Alignment]
3R:6996923-6996943 [Sequence]	Antp	Reverse	21 [Sequence]	21	0.018	100.0 [Alignment]
2R:11507157-11507177 [Sequence]	inv	Forward	21 [Sequence]	21	0.018	100.0 [Alignment]
X:1765234-1765253 [Sequence]		Reverse	20 [Sequence]	20	0.071	100.0 [Alignment]
2R:22328568-22328590 [Sequence]	Gp150	Reverse	23 [Sequence]	19	0.28	95.7 [Alignment]
3L:22388357-22388375 [Sequence]	Ten-m, lncRNA:CR45962	Forward	19 [Sequence]	19	0.28	100.0 [Alignment]
4:290430-290448 [Sequence]	Syt7	Forward	19 [Sequence]	19	0.28	100.0 [Alignment]
X:9520410-9520427 [Sequence]	CG32700	Forward	18 [Sequence]	18	1.1	100.0 [Alignment]
X:16350737-16350754 [Sequence]	sl	Forward	18 [Sequence]	18	1.1	100.0 [Alignment]
3R:7761635-7761652 [Sequence]	Sp7	Reverse	18 [Sequence]	18	1.1	100.0 [Alignment]
3R:15483010-15483031 [Sequence]		Reverse	22 [Sequence]	18	1.1	95.5 [Alignment]
3L:20309157-20309174 [Sequence]	SCCRO4	Forward	18 [Sequence]	18	1.1	100.0 [Alignment]
3L:26762297-26762314 [Sequence]		Forward	18 [Sequence]	18	1.1	100.0 [Alignment]
2L:720411-720427 [Sequence]		Reverse	17 [Sequence]	17	4.4	100.0 [Alignment]

Mus musculus *>mEN2 conserved*

TCAAACTTGCATATGCAACGAGGTGCTGGAGCTTTGTGGCGGAAATATAATTAAACTGGTGAGAGATGTAAAAGATGAAGAATGGGATG ACATCAGACTGATTTCACACTTGGCAGTCCGATAGCTGGGCCCAAGCCGGGCTTGCCACGCAAGGCAGATCAAAGTGCTCTGCCACCAC TAAAAAGGCAAAGGGAGACTTAAGTATGCTAATCCCCAGGACAAATATATTTTAATCTTGTTAGAATACAAGTTAATGCTGCAGTGCAG TGGCTGAACTTGGTCAGGCAGGAGAGACCCATTTTTATCTTAGCTATGAATGAGGTGAAGCTCTTGGTTTTATTCATCAGTAATAAAAG TACATTTATAAAACGAA

Results for mEN2 supercons

Job name	mEN2 supercons
Species	Mouse (Mus musculus)
Assembly	GRCm38
Search type	BLAT

Genomic Location	Overlapping Gene(s)	Orientation	Query start	Query end	Length	Score	E-val	%ID
5:28179290-28179662 [Sequence]		Forward	1	373	373 [Sequence]	725.0	2.2e-209	100.00 [Alignment]

Homo sapiens *> hEN2 conserved*

TCAAACTTGAATATGCAATGAGACGCGGGAGCTTTGTGGCGGAAATATAATTAAACTGGGGAAAGATGTAAAAGCTGAAGAATGGGATG ACATCAGGCTGATTTCACACTCGGCAGTCGGATCGCTGGGCCCAAGCCGCGCTCTTGCCACGCAAGGCAGATCAAAGTGCCCTGCCACC GCTAAAAAGGCAAAAGGGGACTTAAGTATGCTAATCCCCAGGACAAATATATTTTAATCTTGTTAGAATACAAGTTAATGCTGCAGCTC AGTGGCTGAACTTGGTCAGACTGTAGAGATCCATTTTTATTTTAGCTATGAATGAGCTAGACCTCTTGGTTTATTCATCGGTAATAAAA GTAAATTTACAAAACGAA

Results for hEN2 supercons

Job name	hEN2 supercons
Species	Human (Homo sapiens)
Assembly	GRCh38
Search type	BLAT

Genomic Location	Overlapping Gene(s)	Orientation	Query start	Query end	Length	Score	E-val	%ID
7:155471753-155472126 [Sequence]		Forward	1	374	374 [Sequence]	727.0	4.2e-210	100.00 [Alignment]

Note, for *Drosophila melanogaster*, multiple hits are annotated (in order of appearance in BLAST search); however, only the *invected/engrailed* conserved CRE matches the cutoff criteria of >62% sequence identity (column %ID) over at least 55 basepairs (column Length) with minimum 1^{e-1} confidence level as the BLAST e-value (column E-val). In the case of human and mouse searches with BLAT instead of BLAST but using the same cutoff criteria, the only CREs identified are those linked to human *EN2* and mouse *En2*.

Inter-species EMBOSS Matcher sequence comparisons related to *inv/en-EN2* CRE

Shown are screen shots of EMBOSS matcher sequence comparisons using random intergenetic regions (to match the intergenetic topology of *inv-en* CRE) of various species that also vary in length. Note that only *inv/en*-related intergenetic CRE (here shown for chicken) matches the cutoff criteria for a minimum of 62% sequence identity over a length of at least 55 basepairs.

***Gallus gallus Ensgal-En2* intergenetic region against *Drosophila inv-en* CRE**

```
#=======================================
#
# Aligned_sequences: 2
# 1: gEnsgal-En2
# 2: inv-en
# Matrix: EDNAFULL
# Gap_penalty: 16
# Extend_penalty: 4
#
# Length: 65
# Identity:      41/65 (63.1%)
# Similarity:    41/65 (63.1%)
# Gaps:           5/65 ( 7.7%)
# Score: 73
#
#
#=======================================

gEnsgal-En2       87 GATGACATCAGGCCGATTTCACACTTGGCACTCGGATGGCTGGGCC-CAA    135
                     ||.|||..||..|.|....|||||..|.|..|||  .|||||.||| |||
inv-en             9 GAAGACTGCAACCAGGGACCACACACGACGATCG--AGGCTGTGCCACAA     56

gEnsgal-En2      136 GCCGTGCTCTTGCCA    150
                     ||  ||||||.|.||
inv-en            57 GC--TGCTCTGGGCA     69
```

***Danio rerio Sh2d3ca-urm1* intergenetic region against *Drosophila inv-en* CRE**

```
#=======================================
#
# Aligned_sequences: 2
# 1: DrSh2d3ca-urm1
# 2: inv-en
# Matrix: EDNAFULL
# Gap_penalty: 16
# Extend_penalty: 4
#
# Length: 123
# Identity:      76/123 (61.8%)
# Similarity:    76/123 (61.8%)
# Gaps:           5/123 ( 4.1%)
# Score: 156
#
#
#=======================================

DrSh2d3ca-urm 107975 AATATTTCAGTGAATTTAAGA--CTTTTTAAAGCAGAGAATTGTGATTTT 108022
                     |||...|.||||..|||.|||  ||||||||.|.||..|||......|..
inv-en           488 AATTGATAAGTGTGTTTGAGATACTTTTTAATGAAGCTAATGAAACATGG    537

DrSh2d3ca-urm 108023 TGAAATTTAAGACATTTATTTAATTTTTAAGACCTTGCGGACACCCTGTA 108072
                     |||..|||.|||.||.|.|.||..|||.|||...||..|.||.||. |||
inv-en           538 TGAGCTTTCAGATATGTCTATATCTTTCAAGGGTTTAGGCACTCCG-GTA    586

DrSh2d3ca-urm 108073 AT--AACAAATTTTAAAATGTTT 108093
                     ||  ||.|||..||.||||.|||
inv-en           587 ATTTAATAAACATTTAAATCTTT    609
```

***Pan troglodytes HOXA1-HOXA2* intergeneic region against *Drosophila inv-en* CRE**

```
#=======================================
#
# Aligned_sequences: 2
# 1: HOXA1-HOXA2
# 2: inv-en
# Matrix: EDNAFULL
# Gap_penalty: 16
# Extend_penalty: 4
#
# Length: 148
# Identity:      84/148 (56.8%)
# Similarity:    84/148 (56.8%)
# Gaps:          13/148 ( 8.8%)
# Score: 104
#
#
#=======================================

HOXA1-HOXA2     2696 GCAGAGTTTACGGGTCTTAGGATGTCTGTAATAAATAAATATACTCAAGT   2745
                     ||.|||||..||..| ||...|||..|||||||| |..||.||      |
inv-en           398 GCTGAGTTAGCGCCT-TTGCAATGGGTGTAATAA-TTTATGTA------T    439

HOXA1-HOXA2     2746 TTCAGGTATGCTTAAGCATCCGTGTATTTGGCTGGGCTACAATTTGTTAA   2795
                     ||....||.||..|||..||.|||..|.|||||.||||......|.||||
inv-en           440 TTTGATTAGGCCGAAGTTTCTGTGACTGTGGCTCGGCTTTTGCCTTTTAA    489

HOXA1-HOXA2     2796 TTCCTA--TGAAGTTGGCACA---TTTCATGAGGGGAAAGGGAGAAGG   2838
                     ||..||  ||...|||..|.|   |||.||||.|..||.|..|.|.||
inv-en           490 TTGATAAGTGTGTTTGAGATACTTTTTAATGAAGCTAATGAAACATGG    537
```

Mus musculus Hnrnpr-Hfr1d **intergenetic region against** ***Drosophila inv-en*** **CRE**

```
#=======================================
#
# Aligned_sequences: 2
# 1: mHnrnpr-Htr1d
# 2: inv-en
# Matrix: EDNAFULL
# Gap_penalty: 16
# Extend_penalty: 4
#
# Length: 117
# Identity:      70/117 (59.8%)
# Similarity:    70/117 (59.8%)
# Gaps:           9/117 ( 7.7%)
# Score: 114
#
#
#=======================================

mHnrnpr-Htr1d 180662 TTCAATGAGGTAAAGGATTGGATTGGGTGGTCTCAGGTTTATGTGGCTC 180711
                     |||..||||.||..|...|||.|.||||||...|.|  |||||||  .|.
inv-en           395 TTCGCTGAGTTAGCGCCTTTGCAATGGGTGTAATAA--TTTATGT--ATT    440

mHnrnpr-Htr1d 180712 TTGCCCAGTGCCACATTTCCTGGCTTTCAAGCTAGG----TGCTTTTTAT 180757
                     |||...|| |||..|.||.|||....|...|||.||    |||.|||||.
inv-en           441 TTGATTAG-GCCGAAGTTTCTGTGACTGTGGCTCGGCTTTTGCCTTTTAA    489

mHnrnpr-Htr1d 180758 AACGTAAGTCTGTTTGA 180774
                     ....|||||.|||||||
inv-en           490 TTGATAAGTGTGTTTGA    506
```

Homo spaiens ERN1-TEX2 **intergenetic region against** ***Drosophila inv-en*** **CRE**

```
#=======================================
#
# Aligned_sequences: 2
# 1: hERN1-TEX2
# 2: inv-en
# Matrix: EDNAFULL
# Gap_penalty: 16
# Extend_penalty: 4
#
# Length: 92
# Identity:      55/92 (59.8%)
# Similarity:    55/92 (59.8%)
# Gaps:           7/92 ( 7.6%)
# Score: 91
#
#
#=======================================

hERN1-TEX2     36587 TTCGTGCCTT---ATATGTTCTATTCAGTTATTTTTTTTGATAAAAATGA  36633
                     ||.|.|||||   |...|.|.||.|.|.||||.|.|||||||.|....||
inv-en           404 TTAGCGCCTTTGCAATGGGTGTAATAATTTATGTATTTTGATTAGGCCGA    453

hERN1-TEX2     36634 AATATCTTTTTCTGTCCCATTCAATCCTTTTCTTTTTGAATT  36675
                     |.|.|||.|..||||..|  ||.  .|||||...|||.||||
inv-en           454 AGTTTCTGTGACTGTGGC--TCG--GCTTTTGCCTTTTAATT    491
```

Petromyzon marinus **(lamprey)** ***Fgfr1op-tmem*** **intergenetic region against** ***Drosophila inv-en*** **CRE**

```
#=======================================
#
# Aligned_sequences: 2
# 1: lFgfr1op-tmem
# 2: inv-en
# Matrix: EDNAFULL
# Gap_penalty: 16
# Extend_penalty: 4
#
# Length: 136
# Identity:      77/136 (56.6%)
# Similarity:    77/136 (56.6%)
# Gaps:           8/136 ( 5.9%)
# Score: 89
#
#
#=======================================

lFgfr1op-tmem   9546 GCATGTTAATATTTCCATAAAATGGTGATAAATCTTGACTTAAAGCTTTC   9595
                     ||.|.|||||...|...|....|.|.||||..|.||.|  |.|||||...
inv-en           481 GCCTTTTAATTGATAAGTGTGTTTGAGATACTTTTTAA--TGAAGCTAAT    528

lFgfr1op-tmem   9596 GCAAC-TGCAG-GAATTCATATTTTTTTGGGTCTTTC---GGTAAAGCCA   9640
                     |.||| ||..| |..||||.||.|.|.|...||||||   |||..||.||
inv-en           529 GAAACATGGTGAGCTTTCAGATATGTCTATATCTTTCAAGGGTTTAGGCA    578

lFgfr1op-tmem   9641 CGTAGATAAAAATAATAAAAAATATCACGTTTCAAT   9676
                     |...|.|||.. |||||||.|.|...|..|||.|||
inv-en           579 CTCCGGTAATT-TAATAAACATTTAAATCTTTAAAT    613
```

***D. melanogaster invected and engrailed* gene loci and homology to other insects**

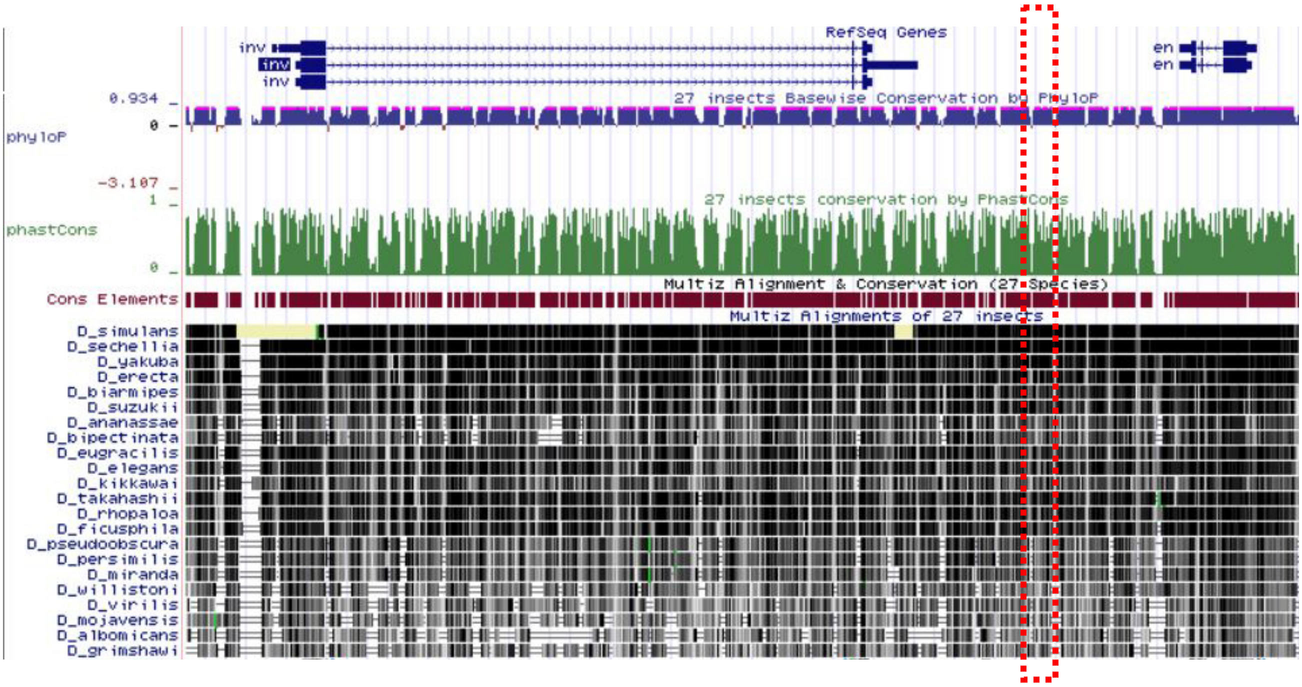

Image capture of UCSC Genome Browser on *D. melanogaster* Aug. 2014 (BDGP Release 6 + ISO1 MT/dm6) assembly, shown are the *invected* and *engrailed* gene loci. RefSeq Genes shows exon (blue bars) and intron structure; in red is shown Ensembl Gene annotation used for genome browsing. Green bar diagram indicates conservation among *Drosophilidae* species which are listed further below (black bars indicate sequence homology). Conserved elements are shown in dark red. Dashed red box indicates topology and extension of *inv/EN2* conserved CRE sequence determined in *Drosophila melanogaster*.

Examples of *inv/en-EN2* conserved sequence found in other *Drosophilidae*

***D pseudoobscura* scaffold**

>chromosome: GCA_000001765.2:3:4589216:4590358:1
GGGCAACGGAAGGCGGCAACATCGACAGTAGTTTTGCGGCTCGTTTGCTGCGTTGACTCCTCGCTGATCATCATCATCGTGCTCATCAT
CATCATCCACAGCCACATCCACAGCCACAGCCACATCCACATCAGGCTCCTCGACGGGTCTGGTCTTGTGTCTGGTGGTGC**TGCGGCGC**
CAAATTGCAGCAAAGTAAAACAGCGTTTAGCATTGTACTTGTAATGATCTTGGTCAGGCAATCCGCAGACTGCAATCTTCTCGAATGGC
ACATCCAGCGAAACGGAAGAGAGTGAGCCCCAAAGATACAGCACAGAGCTTAAGAGAATGCCAGAGAGGAGGAGACAGAGAGAGAGAGA
GAT**ACTCGTTCCCTGAGTTAGCGCCTT**GGAAA**GGGTGTAATAATTTATGTATTTTGATTAGGCCG**CAAGTCGCAGACTGAAACTGAGAC
GAAGACAGAGACAGAGTCAGAGTCGGAGGCTGCATCTTCAAGTATCTTTGAGATGCACAAATTGGA**TTTAATTGATAAATGCATTTGAG**
ATATATTTT

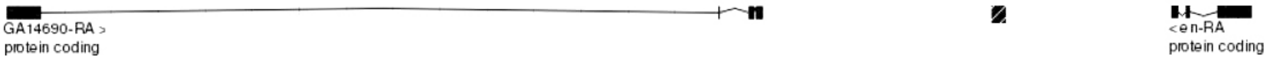

***D simulans* scaffold**

>chromosome:GCA_000259055.1:2R:5926335:5927548:1
TGAAGACTGAAGACTGCAACCAGGGACCACACACGACGATCGAGGCTGTGCCACAAGCTGCTCTGGGCAACGGAAGGCGGCAACATCGA
CAGTAGTTTTGCGGCTCGTTTGCTGCGTTGACTCTTCGCCGAGTCCCGTGATCATGGACATGATCATCATGACGAACGATGATCGTGGG
GTCCGCAGAACGTCATCATCGCGAAGATCTTCCTCCTCCTGATGCGGCGCCAAATTGCAGCAAAGTAAAACAGCGTTTAGCATTGTGCT
TGTAATGATCTTGGTCAGGCAATCCGAGGGCCTGGCGTCCACAAATCTACAGAAGAACCAGAACCCAGGTCTGGAGATTCAAAGAGAGG
TCCCCGAGATTCTTAAGAGAAGACCAGCGCAACTCGTTCGCTGAGTTAGCGCCTTTGCAAAGGGTGTAATAATTTATGTATTTTGATTA
GGCCGAAGTTTCTGTGACTGTGGCTCGGCTTTAGCCTTTTAATTGATAAGTGCGTTTGAGATACTTTTTAATGAAGCTAATGAAGCATG
GTGAGCTTTCAGATATGTCTCTATCTTTCAAGGGCTTAGGCATTGGGGTAGTTTAATACACATTTAAATCTTTGAATGAA

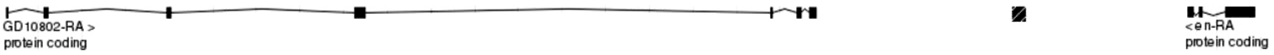

***D yakuba* scaffold**

>chromosome:GCA_000005975.1:2R:9558232:9558991:-1
TAATATGTATGCGATTCGGCGGCAGCCGCAAAAGCATCTGTGCGGAAACTAAGAGCGAGGATCGCCGGGCGAAGAGAGGCTCCGTTTTG
GCAATGGTCGATGGTCGATGCGAGTCCCCGAAAAATGCAC**TGCACTTAAAATTATTGACACTTTG**GCGATC**GATGCGGCTACGTGAGGC**

GGCTACCGTGATGCGGCTAAGAGAGCGGTTAAGAGAGCGAAAAGGGCGGAAGAATCCAGCCGAAAGACAGGCGGGCAGACAGAAGACTG
CAAACTGAAGACTGCAGACTGAAGACTGCAAAC**TGAAGACTGAAGACTGCAACCTGGGACCACACACGACGATCGAGGCTGTGCCACAA**
GCTCCTCTGGGCAACGGAAGGCGGCAACATCGACAGTAGTTTTGCGGCTCGTTTGCTGCGTTGACTCTTCGCCAAGTCGCTTGATCATG
GACATGATCATCATG

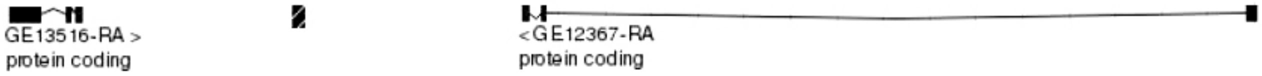

***D willistoni* scaffold**

>supercontig:GCA_000005925.1:scf2_1100000004558:2013519:2014160:-1
AACGATCATCATGATGACGGTTGTTGGGACG**GCGGCGCCAAATTGCAGTAAAGTAAAACAGCGTTTAGCATTG**CAGACTCGTCGTAATG
ATCTTGGTCAGGCAATGTGGCAATGTGCGTTAAATGAAGAGAAAATCTTCCCCAGAGAGAT**TTAAGAGAGTCTTCAAAGTGCTAAA**

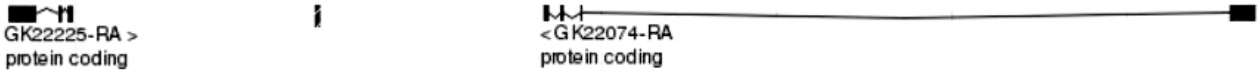

inv/en-EN2 conserved CRE - potential transcription factor binding sites

invected/engrailed, *mEn2* and *hEN2* conserved CRE sequence alignment was generated using MLAGAN (http://genome.lbl.gov/vista/lagan/submit.shtml). Potential transcription factor binding sites were identified using JASPAR (http://jaspar.genereg.net/); potential binding sites are color coded as per legend below.

Seq1 = inv/en supercons; Seq2 = mEn2 supercons; Seq3 – hEN2 supercon

```
sequence1(+)    000000001  TGAAAACTGAAGACTGCAACCAGGGACCACACACGACGATCGAGGCTGTG  000000050
sequence2(+)    000000001  TCAAACTTGCA-----------------------------------TATG  000000015
sequence3(+)    000000001  TCAAACTTGAA-----------------------------------TATG  000000015

sequence1(+)    000000051  CCACAAGCTGCTCTGGGCAACGGAAGGCGGCAACATCGACAGTAGTTTTG  000000100
sequence2(+)    000000016  CAACGAGGTGCT-----------------------------GGAGCTTTG  000000036
sequence3(+)    000000016  CAATGAGACGCG-----------------------------GGAGCTTTG  000000036

sequence1(+)    000000101  CGGCTCGTTTGCTGCGTTGACTCTTCGCCGAGTCCCGTGATCATGGACAT  000000150
sequence2(+)    000000037  TGG--------------------------------------CGGAAATAT  000000048
sequence3(+)    000000037  TGG--------------------------------------CGGAAATAT  000000048

sequence1(+)    000000151  GATCATCATGACGTACGATGATCGTGGGGTCCGCAGAACGTCAT--CATC  000000198
sequence2(+)    000000049  AATTAAACTGGTGAGAGAT----GTAAAAGATGAAGAATGGGATGACATC  000000094
sequence3(+)    000000049  AATTAAACTGGGGAAAGAT----GTAAAAGCTGAAGAATGGGATGACATC  000000094

sequence1(+)    000000199  GCGAAGATCTTCCTCCT-----CCTGATG--CGGCGCCAAATTGCA----  000000237
sequence2(+)    000000095  AGACTGATTTCACACTTGGCAGTCCGATAGCTGGGCCCAAGCCGG--GCT  000000142
sequence3(+)    000000095  AGGCTGATTTCACACTCGGCAGTCGGATCGCTGGGCCCAAGCCGCGCTCT  000000144

sequence1(+)    000000238  -GCAAAGTAAAACAGCGTTTAGCATTGTGCTTGTAATGATCTTGGTCAGG  000000286
sequence2(+)    000000143  TGCCACGCAAGGCAG-----------------------------------  000000157
sequence3(+)    000000145  TGCCACGCAAGGCAG-----------------------------------  000000159

sequence1(+)    000000287  CAATCCGAGGGCCTGGCGTCCAC---AAATCTACAGAAGAACCCAAGTCT  000000333
sequence2(+)    000000158  --ATCAAAGTGCTCTGCCACCACTAAAAAGGCAAAGGGAGACTTAAGTAT  000000205
sequence3(+)    000000160  --ATCAAAGTGCCCTGCCACCGCTAAAAAGGCAAAAGGGGACTTAAGTAT  000000207

sequence1(+)    000000334  GGAGATTCAAAGAGAGGTCCCCGAGATTCTGGGCGACTTAAGAGAAGAAC  000000383
sequence2(+)    000000206  GCTAATC-----------------------------CCCAGGACAAATAT  000000226
sequence3(+)    000000208  GCTAATC-----------------------------CCCAGGACAAATAT  000000228

sequence1(+)    000000384  AGCGCAA-CTCGTTCG---CTGAGTTAGCGC--CTTTGCAATGGGTGTAA  000000427
sequence2(+)    000000227  ATTTTAATCTTGTTAGAATACAAGTTAATGCTGCAGTGCAGTGGCTG---  000000273
sequence3(+)    000000229  ATTTTAATCTTGTTAGAATACAAGTTAATGCTGCAGCTCAGTGGCTG---  000000275

sequence1(+)    000000428  TAATTTATGTATTTTGATTAGGCCGAAGTTTCTGTGACTGTGGCTCGGCT  000000477
sequence2(+)    000000274  ----------AACTTGGTCAGGCAGGA-----------------------  000000290
sequence3(+)    000000276  ----------AACTTGGTCAGACTGTA-----------------------  000000292

sequence1(+)    000000478  TTTGCCTTTTAATTGATAAGTGTGTTTGAGATAC-TTTTTAATGAAGCTA  000000526
sequence2(+)    000000291  ---------------------------GAGACCCATTTTTATCTTAGCT-  000000312
sequence3(+)    000000293  ---------------------------GAGATCCATTTTTATTTTAGCT-  000000314

sequence1(+)    000000527  ATGAAACATGGTGAGCTTTCAGATATGTCTATATCTTTCAAGGGTTTAGG  000000576
sequence2(+)    000000313  ATGAATGAGGTGAAGCTCTTGGTTTTATTCAT------------------  000000344
sequence3(+)    000000315  ATGAATGAGCTAGACCTCTTGG-TTTATTCAT------------------  000000345

sequence1(+)    000000577  CACTCCGGTAATTTAATAAACATTTAAATCTTTAAATGAA  000000616
sequence2(+)    000000345  -----CAGTAA-TAAAAGTACATTTATA-----AAACGAA  000000373
sequence3(+)    000000346  -----CGGTAA-TAAAAGTAAATTTACA-----AAACGAA  000000374
```

AAATTGC = Distalless (Dll)
CAATCC = ocelliless/orthodenticle (oc/otd)
CAAAGTAAAA = tailless (tll)
AGATAC = sine occulis (so)
TGAAAC = so
TTAATGA = empty spiracles (ems), Sex combs reduced (Scr), unplugged (unpg)
TTTGATTA = invected (inv) and engrailed (en) as well as unpg
ATTTAATA = extradenticle (exd)

SUPPLEMENTAL DATA SET S3

dachshund/DACH1 conserved CRE sequence

R65A11 - dac

```
>BDGP6:2L:16481227:16484138:1
CGAAAATATAGCCCTTTTTCACGCTCTATTTATAGCATTCACATTCTTTCTTTTTTTTTGCACTTTTTAGCTGGCATATCCTTTCGACT
TCCGCCATTCGAGGCTCGCCCAATTTCCGTTTCGAGTTTAATTAATTTAATAAACAAATTCTTTTCGCTCTAAAAACTCTCAAGTGTAT
CGATACGATGCGTTTCTTTTTTTCCTTCGTTAAATAAATAATAACCAAAAAAAAAAAAACCAAAAAGTAGGAGGAGAAAAGTTATTGC
CATAGTTTTTTATTATACTTGTGTGTTTACCTTTCTGGTGGCTTGATCGATAGGCATCTGCAATTAAAAAGAGAAGAAGAAGAGACAA
GTGAGGCAAAATTGTTAAACGTTTTGTGTAAGCTTTAATACGAAAAACAAGTACTGCAACATAACGGAAGGAAACACGGCTTAAATTCG
GGGCACAAATGCTGAAAGGGAAGTTTTTCATTGACGGGTTCGTTCTGACGGACTTGCATTTTGGCGGGCAAGCGGGTGTGAAAATGCAC
ACGCCCCGAGAACCCCCCTTTCCACCCCCCCTGGACCCCTTTATCCAGCCCACTGGCCAAAAACAATTTGTAATTATCCACAGAGAGCG
CTGCCTTCAGCGGTTTCGCATTTCCCCTTTCGCTCGCTCTCCCAACTTGTTTCAATTTAGCGCAAAACTTTTTCAACCTAATAATAGGT
TTAACCGCATTTTTAACCGTTCCTCATGTTCGGTCCGGTTCGGTTTTCAAAACCGGGAATCGTACTTAGACTGGGTCTCCTTATTTCTG
TTCTGGCTCTCTGTACAACTTTTCATTGAGAAAAATGTAACTAGTTTTTCATAGCAACGGAATACAATTTAATCCAATAATCCAATAGT
TTAATCCAATACAAATGATATTACTACCATTTCTATTTTCGTTAATTTCGATTTGACTTATTTGGCTGGATTTACTTTTCAAAATATAT
GTTATCAATAAGACACAAACCTTACTTTTCTAGCTATTAACATAGTTTAAAAAAAAAAAAAAAACTAATAAAAATTACGTGAATCTAAAT
TTTTAAACCCGATATCCAAGAAGATCTCAATTTTTGCCTGTGTACTCAGTTCTCTGAACAAAGCGCATGTGCACTTTGGAGCACACTCC
ATACATGTGGCTCAGCCCTTTTCCATAATTAACTAGATGGTTTTCCATCGACTTCATTGTGGTCAGCGGCCAGTTCAACCCGTTCTTCA
CTGCAACCGAGAACTGTAAACACAAAAACCCCAGGACTCTACATTGGCTTAAAAAAATAAGAACAGAACAGAACCAAAACCAAAAAAAG
AAGGGATATTGAAATACAAGGTTGTAATCGTTTCGACTGTTGATGTCTCAATGCATGGGCAGTTCAGTTAGTAAATGTTTTTCAAAATC
TTTCAGGCAGGAGATGTTTAAATATCCATGAAATATTTGGATCTCCTGGGGATCAATCGGAATATTAGCCTTTAATTGTGTTGATCTTT
TAAGCCTTTTTGTATCTAATCTAAGCCATTCGATCTAATCACAATTTATAAATATCTGCATATTTCTGTATAAGTCTGCATCATTTGAC
GTAACTCTTTAAGTCTTTTGGCTTAAGTTGCAACTATAAGGAAGTATTTATTTTAGAGACACAAATATTTCAGTCGCCTTCATTTGAAC
AAATCGGCGAAAATTGGCTAGCTCGCCAAACTTTCTGTAACCAAGGACAATGGTTTTATTTTAAACCATTAAAAACTTTAGACCCACTA
GCTCCTAGATCCCCCTCAAAAGATTTAAAAAAAAAAAACACGATACCCATTTCTACTGAACTTCGTTTTTGCTTGTCGTTTTTTCCACT
CGAACGGAAATGAGCTGACAGCGCACCGCACACGTCGATTGCAGAAAAACATCGGATAAAACAGGAGGAAAAGTTGTGCAAGGTGGAAA
ACTGTTTTACCAACTATTGTTAGAGGCGTTCAAAAGAATTACGCAGCTTTCGGTTAGTTAGCAAGGGGTCACCGGGGAGCGTTACGTTT
GCATTGCGTATTTCCGCTAAATGTCATCGGAAAAGGCAAACGGCGAAATGCGAAACGAAAGTTTTTTGATTGCCCGTGTTAATCGATAT
CGATGCACAAACTATTTGCATTGCAACCGTTGCAAGAATATGCAAGAAGTTGGGGGCGGCCGCGGCAGGGGGTGGAAGTTGAGTGCGTA
AGTTGGCTAAAGCGGAAACAGGAAATGAGAAAATTTTGCAGAGCAAACCCCGAACTGGAAATGCAACTAACTGGGCACATGCACTTTGC
GAAATCATTGGATAGCGTTAAGAAATTTATTTTAAAATTGTAACTAACATTTAATCGTATTCAAAAGCAATTAAATCCCAATCCAATTC
TTATATAAAATCCTTACAAGATTATTCTATTTACTGTAAATCTAAGCAAAAACTCCCTTTGCAAAATATTCGCCTGCACAGCACAGATC
AGTGAAATAATCAAATGAAGTCTTGAAATAACGAAAAACCCCCAATTGCGTGTGGAACTGCCCCCAATGCTTTTGCTTCGGTTTCGTAC
CTGGCCGTGGTGCAGTCCCTGTAGAGGATGTCGAAGTCCTTGCAGCAGAGCAACTTGCAGCGATTGACGCCCGGCTGGATGGCGCCCAA
GCCGCGTAATATGCGCACCTGTTCCACATTGCAGACGAGCGGTACGATGTCAAGGCGCTTTAGTTTGGTATAAACCGTATGCAGACCGC
CGACCAAGTGCTTCAGGAAGAGCTCGAAGGCCTGCGGCAGGCAGAGCATCGTTTCGTTGCTAATTATGAATGCGGCGACCTTCTGACCC
CGGTACTCCACCAGCTTGCACTCATTGGCACTGGGATCCGAGGTGGAGATCGGCGGCGGTGAGT
```

Yellow highlighted sequence depicts dac conserved sequence

EMBOSS MATCHER R65411 - dac conserved

```
# Length: 247 - # Identity:     247/247 (100.0%) - # Similarity:   247/247 (100.0%)
# Gaps:            0/247 ( 0.0%) - # Score: 1235
R65A11dac         79 TCCTTTCGACTTCCGCCATTCGAGGCTCGCCCAATTTCCGTTTCGAGTTT    128
                     ||||||||||||||||||||||||||||||||||||||||||||||||||
Dacyellow          1 TCCTTTCGACTTCCGCCATTCGAGGCTCGCCCAATTTCCGTTTCGAGTTT     50

R65A11dac        129 AATTAATTTAATAAACAAATTCTTTTCGCTCTAAAAACTCTCAAGTGTAT    178
                     ||||||||||||||||||||||||||||||||||||||||||||||||||
Dacyellow         51 AATTAATTTAATAAACAAATTCTTTTCGCTCTAAAAACTCTCAAGTGTAT    100

R65A11dac        179 CGATACGATGCGTTTCTTTTTTTCCTTCGTTAAATAAATAATAACCAAAA    228
                     ||||||||||||||||||||||||||||||||||||||||||||||||||
Dacyellow        101 CGATACGATGCGTTTCTTTTTTTCCTTCGTTAAATAAATAATAACCAAAA    150

R65A11dac        229 AAAAAAAAACCAAAAAGTAGGAGGAGAAAAGTTATTGCCATAGTTTTTT    278
                     ||||||||||||||||||||||||||||||||||||||||||||||||||
Dacyellow        151 AAAAAAAAACCAAAAAGTAGGAGGAGAAAAGTTATTGCCATAGTTTTTT    200

R65A11dac        279 TATTATACTTGTGTGTTTACCTTTCTGGTGGCTTGATCGATAGGCAT    325
                     |||||||||||||||||||||||||||||||||||||||||||||||
Dacyellow        201 TATTATACTTGTGTGTTTACCTTTCTGGTGGCTTGATCGATAGGCAT    247
```

hs137 DACH1 intragenic enhancer

```
GCAATTTTGAAAAAGAAAACAATGGTTAGGAAGACTTTATTTTATTCTAATTATATTTGATACTTTAAATGTTTATCTATCTAATATAT
CTCTTATTAAACAATTCTTTAGCTCTGGTATATTTTTTAGTAAAGTTTTAATAACTTTCTCAGAATTAAAAATAGCATATGATATCAGA
```

GACCAACAATAAAAGTAATCGAATTTAGAGAAATGTTTAGAAAATAAATAGATCTATACATATTTTGTTAAAGACCAATCAATTGATCT
GCTGTTTTCTATGTAAAATGAAACAATGCTTATTATTAAAATATAGGCTGAATTCTTTGCCTGATTTTCCGCTTTTGTAAAATGCATTG
CTTGAGAAACAAGTCTTCAAAAAACAAGGCCTAAAAAAATCTACTACTCAGGGTGCCTTTGAGGATATTTAATTAAAATCTAATCCTGC
ATTCATTAAGGCTCACATAAATTAAGCTGTCATTCATAAGATTTATGGATTCTCATTTGCATATTGCATACAATTCATCAATTACTCAA
GTATGAAAGGAGCACATTTCCCTTGGAGCTGCCTGCTACCCTGCCAACATTTGAAATGAGGGAAAGAGCAAGACTGTCAGGCATTCACA
CAAACTTTCTTCCAAATGTCTGCTCCTTGATTAATCTAATTTTCTAGATCTTCCCTACAAGATACACCAACAGCCCTGGTGCACATTTC
TATTATTTCGCCTGTCTTCATCACCAAAATCTTCCAGTCTTTGAATACTTCTTCATGTCTGAGTGATTATTCTTTATTTGCTAATAATA
TGGCATTGATGATATTTCCCTATTCAAATGTGTTTGATATGTACAAAGATGTAGGTTATAGGTCTGTTGATTTTTAATGTTTATTATTT
GCTATCAACAATCAGCCAATAAAGAGCTGAAAATGAGGACATGGAAAAGGAAGAGAATGTTGCTGTGGAATGTAGTTGGCACAAAAGCC
TAGTTTCAGAGACTGTTAGCATTTCAGAAGAACCTGAAACAAAGCCCTGTAGTAAATCACTGATTCTATATTTAATATAAAAAATGGAG
TTCACTCAATCAGCTGCAAGTATATGCTACTGCACTTTTTGCAGTTCTAAGCAAAGTAGTAAGTAACCCAAATTAGTTTGTTTTCACTG
TGCATAAAGCATACTGTGTTAATTCACTTGTCTTCTCTGGGGAGTAGATCTTAATTTCAGTGGCACAAGTTGGAAGTAACAGTCCTTTC
TAGACCACACTCTGGAAACCTTCCAACAGGAATGTGATCAGGTGCGAAACTTAATGCAAAACACTCCTCATGTCCAGGATTTCTACAAT
TAAAATATATGAATGTATCAGCACTCTCAAAAATCTCTGCTCCTGAAAACATGCTGTCAATTAAATGTCAATTAAATAGAAAAACGGGA
AAAACTACCTTTCAAAATCATTAGTTTTCTATGAAGTGCATATACTTCAGTATAAAAAGAAAGCATTTTATTCAATTTAAATATTTAGA
AAATTCATTTGGTAACATGAGGGATTTTATTTTACTGTATGATACTTGCCAAAACTATGATCATATCTCTTTGGAAACACCCATTTAGA
ACCCAAAATTAGAATTTCCCAAATTAAAACCTTAACATCTCAGGTTAAGTGTTAAATTACCAAAGACACCTTTTGGTTGATTTTGCTCC
CGAAGAGTCTAAAGAAATGAGTAAATGACTCTAAGGACTATACTAGCATAATCATAAATATTTTCACTTTACAAAAACTGTGCCTCTAA
AGAGAAAAAAAATTGTATTCAGCAATAAAAATAATTTCCATGAATTCCTCACAAATACATTTAACATAGGAGAAATAAATGACTCTATAA
ATTTAATCAGAAGGATGAAATCATTAGCATCCCCAATTTATAAAATGACTTTCAAAACTGAAAGCAAACAACAGATAAGCTCATGCTTT
TTATTCATTCAAGTGGGATGGTTCTTCATAATTTTCAGAGATCCATGCCTGAGACACTACTGAGTGTATTGTCTGTCTTTTAACTCTTG
CATTCTCTACATTTCAGTTTTCTCAGCATGAGCTGTCTA

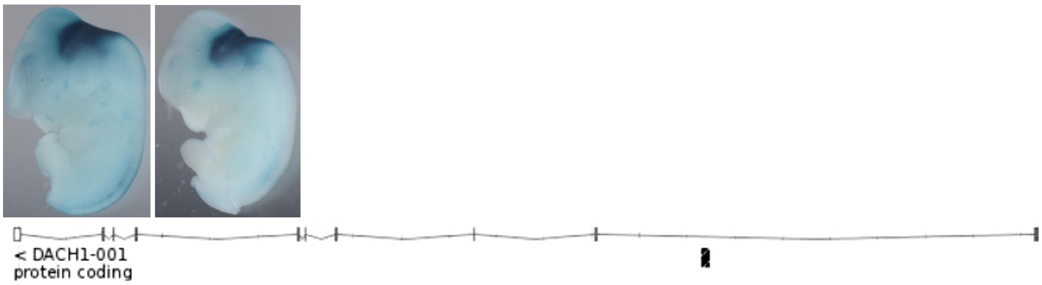

Left, two examples of hs137 driven lacZ expression in mouse E11.5 brain specific to MHB (VISTA database, with permission; data from ref. 54); right, intragenic location of hs137 enhancer element within human DACH1 locus.

EMBOSS MATCHER hs137 – dac conserved

```
# Length: 96 - # Identity: 60/96 (62.5%) - # Similarity: 60/96 (62.5%)
# Gaps: 5/96 (5.2%) - # Score: 108

hs137DACH1int   1049 ATTTAATATAAAAAATGGAGTTCACTCAATCAGCTG-CAAGTATAT-GCT   1096
                     |||||||| ||.||||....|||.|||.|..|.||. |||||.||| |.|
Dacsupercons      56 ATTTAATA-AACAAATTCTTTTCGCTCTAAAAACTCTCAAGTGTATCGAT    104

hs137DACH1int   1097 AC--TGCACTTTTTGCAGTTCTAAGCAAAGTAGTAAGTAACCCAAA   1140
                     ||  |||..||.||....|.||..|..||.||...|.|||||.|||
Dacsupercons     105 ACGATGCGTTTCTTTTTTTCCTTCGTTAAATAAATAATAACCAAAA    150
```

dac conserved

TCCTTTCGACTTCCGCCATTCGAGGCTCGCCCAATTTCCGTTTCGAGTTTAATTAATTTAATAAACAAATTCTTTTCGCTCTAAAAACT
CTCAAGTGTATCGATACGATGCGTTTCTTTTTTTCCTTCGTTAAATAAATAATAACCAAAAAAAAAAAAAAACCAAAAAGTAGGAGGAGA
AAAGTTATTGCCATAGTTTTTTTATTATACTTGTGTGTTTACCTTTCTGGTGGCTTGATCGATAGGCAT

mDach1 conserved

GGTTAAAGTGAATATTTTCAGCGTGAACTTGTCTCTTAATGTCCATTAGACTGACTTTCTTGCCCTTTGTAGCACATTTGTATTCTGTG
GAGAAAAGGAAAATTGATCCCTGAGGCCACTAATGGAAACACTTTCTATACCAGTTTTCAGTTTTTCAATTGATTGAATTAGATTTTAG
AAATGTTTAGACTAACAATATATGAGTTTAACCGAAAAATAGAAGAAGAAAAACAGCCAAGAAACTGGTAGTAAATGAGCCTTTATTGC
CTCAGGCATTGGCCTAGTTAATAAACTTTCATTGAAGGTTTCTTTCAACTGTTACGGACAATGAGAGGGGGAAAAAATCTTAAATTACA
GCCATGTGTGACTTTTAAAGCAGTCATTCTTTTTTATCTCAATACAACATTCCTTTGTCTAGCATCTCCATGAAAATTATCATTTGAAA
GGTTTATATCTTAAATAAAGGGGAAAAAACCATTTTCTTCTAAATACAATTAAAACTAGTTTGAAAAGTAAGAGGCTCAGGAAACAAGA
TCTGAATAAGTAATGTATTAGCAGGTAGAACATCTTGAAAACTTACTAAGAATGCT

hDACH1 conserved

GGTTAGACTGAAGATTTTGAGTGTGAACTTTTCTCTTAATGTCCATTAGACTGACTTTTCTTGGCCTTTGAAACACATTTGTATTCTGC
AGAGAAAAAGAAAAATTGATCCTTGAGGCCATTAATGGAAACACTCCCTATACCAGTTTTGAGTCTTTCAACTGATTGAATTAGATTTT

TTAAATGTTTGTACTAACAACATATGAGTTTAACCGAAAAATAGAAGAAGAAAAAACAGCCAAGAAACTGGTAATAAATGAACCTTTATT
GCCTCAGGCATTGGCCTAGTTAATAAACTTTCATTGAAGGTTTCTTTCAACTGTTACGGACAATGAGAGGAAAAAAATATTAAATTACA
GCCATGTGTGACTTTTAAAGCAGTCATTCTTTTTTATCTCAATACAATATTCCTTTGTCTAGTATTTCCATGAAAATTATCATTTGAAA
GGTTTATATCTTAAATAAAGAAAAAAGTTTCTTCCAAATACAACAAAAACTAGTTTAGAGAAATAGTATGCTGAATAAATGAGATCTAA
AAATATGCTACAACCATATTCATATGTAACATCTGAAAACCTATTAAGAGTGTT

BLAST search results using *dachshund/DACH1* conserved sequence

Shown are screenshots of the respective BLAST searches.

Drosophila melanogaster >dac conserved

*TCCTTTCGACTTCCGCCATTCGAGGCTCGCCCAATTTCCGTTTCGAGTTTAATTAATTTAATAAACAAATTCTTTTCGCTCTAAAAACT
CTCAAGTGTATCGATACGATGCGTTTCTTTTTTTCCTTCGTTAAATAAATAATAACCAAAAAAAAAAAAAACCAAAAAGTAGGAGGAGA
AAAGTTATTGCCATAGTTTTTTTATTATACTTGTGTGTTTACCTTTCTGGTGGCTTGATCGATAGGCAT*

Results for dac supercons

**Job name**	dac supercons
**Species**	Drosophila melanogaster
**Assembly**	BDGP6.22
**Search type**	BLASTN (NCBI BLAST)

Genomic Location	Overlapping Gene(s)	Orientation	Length	Score	E-val	%ID
2L:16481305-16481551 [Sequence]	dac	Forward	247 [Sequence]	247	9.4E-138	100.0 [Alignment]
2L:11885190-11885215 [Sequence]	Pde1c	Forward	26 [Sequence]	22	0.0017	96.2 [Alignment]
3R:8043864-8043893 [Sequence]	CG43060	Reverse	30 [Sequence]	22	0.0017	93.3 [Alignment]
2L:6448983-6449003 [Sequence]	retm	Forward	21 [Sequence]	21	0.0069	100.0 [Alignment]
2L:16962850-16962877 [Sequence]		Forward	28 [Sequence]	20	0.027	92.9 [Alignment]
3R:734997-735024 [Sequence]	Myo81F	Reverse	28 [Sequence]	20	0.027	92.9 [Alignment]
3R:2269896-2269923 [Sequence]	Myo81F	Forward	28 [Sequence]	20	0.027	92.9 [Alignment]
3R:13314161-13314188 [Sequence]		Reverse	28 [Sequence]	20	0.027	92.9 [Alignment]
2R:17168200-17168217 [Sequence]		Reverse	18 [Sequence]	18	0.42	100.0 [Alignment]
2R:17931574-17931595 [Sequence]		Forward	22 [Sequence]	18	0.42	95.5 [Alignment]
2R:23657555-23657572 [Sequence]	Pde8	Reverse	18 [Sequence]	18	0.42	100.0 [Alignment]
211000022280333:1-18 [Sequence]		Forward	18 [Sequence]	18	0.42	100.0 [Alignment]
2L:6449261-6449282 [Sequence]	retm	Reverse	22 [Sequence]	18	0.42	95.5 [Alignment]
2L:11004516-11004533 [Sequence]		Forward	18 [Sequence]	18	0.42	100.0 [Alignment]

Mus musculus *>mDach1 conserved*

GGTTAAAGTGAATATTTTCAGCGTGAACTTGTCTCTTAATGTCCATTAGACTGACTTTCTTGCCCTTTGTAGCACATTTGTATTCTGTG
GAGAAAAGGAAAATTGATCCCTGAGGCCACTAATGGAAACACTTTCTATACCAGTTTTCAGTTTTTCAATTGATTGAATTAGATTTTAG
AAATGTTTAGACTAACAATATATGAGTTTAACCGAAAAATAGAAGAAGAAAAACAGCCAAGAAACTGGTAGTAAATGAGCCTTTATTGC
CTCAGGCATTGGCCTAGTTAATAAACTTTCATTGAAGGTTTCTTTCAACTGTTACGGACAATGAGAGGGGGAAAAAATCTTAAATTACA
GCCATGTGTGACTTTTAAAGCAGTCATTCTTTTTTATCTCAATACAACATTCCTTTGTCTAGCATCTCCATGAAAATTATCATTTGAAA
GGTTTATATCTTAAATAAAGGGGAAAAAACCATTTTCTTCTAAATACAATTAAAACTAGTTTGAAAAGTAAGAGGCTCAGGAAACAAGA
TCTGAATAAGTAATGTATTAGCAGGTAGAACATCTTGAAAACTTACTAAGAATGCT

Results for mDach1 supercons

**Job name**	mDach1 supercons
**Species**	Mouse (Mus musculus)
**Assembly**	GRCm38
**Search type**	BLAT

Genomic Location	Overlapping Gene(s)	Orientation	Query start	Query end	Length	Score	E-val	%ID
14:97981009-97981208 [Sequence]	Dach1	Forward	199	398	200 [Sequence]	386.0	2.1e-107	100.00 [Alignment]
14:97976595-97976792 [Sequence]	Dach1	Forward	1	198	198 [Sequence]	382.0	4.2e-106	100.00 [Alignment]
14:98083822-98083963 [Sequence]	Dach1	Forward	399	540	142 [Sequence]	272.0	2.9e-73	100.00 [Alignment]
14:98084257-98084306 [Sequence]	Dach1	Forward	541	590	50 [Sequence]	96.0	3.7e-20	100.00 [Alignment]

Homo sapiens *>hDACH1 conserved*

GGTTAGACTGAAGATTTTGAGTGTGAACTTTTCTCTTAATGTCCATTAGACTGACTTTTCTTGGCCTTTGAAACACATTTGTATTCTGC
AGAGAAAAAGAAAAATTGATCCTTGAGGCCATTAATGGAAACACTCCCTATACCAGTTTTGAGTCTTTCAACTGATTGAATTAGATTTT
TTAAATGTTTGTACTAACAACATATGAGTTTAACCGAAAAATAGAAGAAGAAAAACAGCCAAGAAACTGGTAATAAATGAACCTTTATT
GCCTCAGGCATTGGCCTAGTTAATAAACTTTCATTGAAGGTTTCTTTCAACTGTTACGGACAATGAGAGGAAAAAAATATTAAATTACA
GCCATGTGTGACTTTTAAAGCAGTCATTCTTTTTTATCTCAATACAATATTCCTTTGTCTAGTATTTCCATGAAAATTATCATTTGAAA
GGTTTATATCTTAAATAAAGAAAAAAGTTTCTTCCAAATACAACAAAAACTAGTTTAGAGAAATAGTATGCTGAATAAATGAGATCTAA
AAATATGCTACAACCATATTCATATGTAACATCTGAAAACCTATTAAGAGTGTT

Results for hDACH1 supercons

**Job name**	hDACH1 supercons
**Species**	Human (Homo sapiens)
**Assembly**	GRCh38
**Search type**	BLAT

Genomic Location	Overlapping Gene(s)	Orientation	Query start	Query end	Length	Score	E-val	%ID
13:71639315-71639514 [Sequence]	DACH1	Forward	1	200	200 [Sequence]	385.0	3.1e-107	100.00 [Alignment]
13:71643634-71643831 [Sequence]	DACH1	Forward	201	398	198 [Sequence]	381.0	6.1e-106	100.00 [Alignment]
13:71772719-71772862 [Sequence]	DACH1	Forward	399	542	144 [Sequence]	275.0	5.3e-74	100.00 [Alignment]
13:71773156-71773201 [Sequence]	DACH1	Forward	543	588	46 [Sequence]	88.0	8.4e-18	100.00 [Alignment]

Note, for *Drosophila melanogaster*, multiple hits are annotated (in order of appearance in BLAST search); however, only the *dachshund* conserved CRE matches the cutoff criteria of >62% sequence identity (column %ID) over at least 55 basepairs (column Length) with minimum 1^{e-1} confidence level as the BLAST e-value (column E-val). In the case of human and mouse searches with BLAT instead of BLAST but using the same cutoff criteria, the only CREs identified are those linked to human *DACH1* and mouse *Dach1*.

Inter-species EMBOSS Matcher sequence comparisons related to *dac-DACH1* CRE

Shown are screen shots of EMBOSS matcher sequence comparisons using genomic regions of random genes of various species that also vary in length and exon-intron structure. Note only *dachshund*-related genomic region (here shown for mouse) matches the cutoff criteria for a minimum of 62% sequence identity over a length of at least 55 basepairs.

***Mus musculus Dach1* genomic region against *Drosophila dachshund_R65A11* CRE**

```
#=======================================
#
# Aligned_sequences: 2
# 1: mDach1
# 2: dac_R65A11
# Matrix: EDNAFULL
# Gap_penalty: 16
# Extend_penalty: 4
#
# Length: 87
# Identity:      55/87 (63.2%)
# Similarity:    55/87 (63.2%)
# Gaps:           8/87 ( 9.2%)
# Score: 99
#
#
#=======================================

mDach1           432 ATTATCATTTGAAAGGTTTATATCTTAAATAAAGGGGAAAAAACCATTTT    481
                     |||..|.|||.|||. |.|||.|..|.|||||   |..|.|||||.|..|
dac_R65A11       960 ATTTACTTTTCAAAA-TATATGTTATCAATAA---GACACAAACCTTACT   1005

mDach1           482 CTTCTAAATACAATTAAAACTAGTTTGAAAAGTAAGA    518
                     .|||||..|   |||||.| ||||||.||||..||.|
dac_R65A11      1006 TTTCTAGCT---ATTAACA-TAGTTTAAAAAAAAAAA   1038
```

***Petromyzon marinus* (lamprey) *Infalp6* genomic region against *Drosophila dachshund_R65A11* CRE**

```
#=======================================
#
# Aligned_sequences: 2
# 1: lInfalp6
# 2: dac_R65A11
# Matrix: EDNAFULL
# Gap_penalty: 16
# Extend_penalty: 4
#
# Length: 197
# Identity:     118/197 (59.9%)
# Similarity:   118/197 (59.9%)
# Gaps:          16/197 ( 8.1%)
# Score: 154
#
#
#=======================================

lInfalp6      284558 TACCAATT-TATTTTATTTTATTTTGAAGTACCCT-TTGAGCT--AAGAA 284603
                     |||||.|| ||||||..||.||||.||..|..|.| ||..|||  |...|
dac_R65A11       915 TACCATTTCTATTTTCGTTAATTTCGATTTGACTTATTTGGCTGGATTTA    964

lInfalp6      284604 GCTCTCAAGTTGGGTTTCATCAATACAA-ACACACCGTTTCTGTGCCAGA 284652
                     ..|.||||..|...|.|.||||||||..| |||.||| ||.||.|.|.||.
dac_R65A11       965 CTTTTCAAAATATATGTTATCAATAAGACACAAACC-TTACTTTTCTAGC   1013

lInfalp6      284653 CAT-AACGCATTAGATAAAAATAAATTAATAATGAAAAGGTAAGA-AATC 284700
                     .|| |||..|.|..|.|||||.|||..||..|..||||..||.|. ||||
dac_R65A11      1014 TATTAACATAGTTTAAAAAAAAAAAAAAACTAATAAAAATTACGTGAATC   1063

lInfalp6      284701 ATATCATTTAAA----ATATCAAATGTATCTTTTAAAAGTTTTAGCC 284743
                     ..|...||||||    |||||.|| |.|..|.|.||   ||||.|||
dac_R65A11      1064 TAAATTTTTAAACCCGATATCCAA-GAAGATCTCAA---TTTTTGCC   1106
```

***Danio rerio Wnt1* genomic region against *Drosophila dachshund_R65A11* CRE**

```
#=======================================
#
# Aligned_sequences: 2
# 1: DrWint1
# 2: dac_R65A11
# Matrix: EDNAFULL
# Gap_penalty: 16
# Extend_penalty: 4
#
# Length: 221
# Identity:     125/221 (56.6%)
# Similarity:   125/221 (56.6%)
# Gaps:          21/221 ( 9.5%)
# Score: 145
#
#
#=======================================

DrWint1          774 AAGGAAATTAAAAATGATACACAAAGTAGAAAAGA--AATACCAGTATTT    821
                     ||.|.|||..||..|.||.||..|| |..||.||.  ||| |||.||...
dac_R65A11       857 AACGGAATACAATTTAATCCAATAA-TCCAATAGTTTAAT-CCAATACAA    904

DrWint1          822 ATCATCAATTTAAAACACTGTAATTTGTTTTAATTAAGG-----CATAAT    866
                     ||.||  |||...|.||.|...||||...||||||..|.     |.||.|
dac_R65A11       905 ATGAT--ATTACTACCATTTCTATTTTCGTTAATTTCGATTTGACTTATT    952

DrWint1          867 GAACTGAATATATTGTATAACGCATAAGTAATAAAATAAGAAA--AATGA    914
                     ...|||.||.||.|.|..||...|||.||.||.|| |||||.|  ||...
dac_R65A11       953 TGGCTGGATTTACTTTTCAAAATATATGTTATCAA-TAAGACACAAACCT   1001

DrWint1          915 TAATAGTAAAATTATTAA-------TAAAAAAAAAAAATATATATATATC    957
                     ||.|..|..|..||||||       |||||||||||||.|..||...|..
dac_R65A11      1002 TACTTTTCTAGCTATTAACATAGTTTAAAAAAAAAAAAAAAACTAATAAAA   1051

DrWint1          958 TTTAGTTTAATTTACATTTTT    978
                     .|||..|.|||.||.||||||
dac_R65A11      1052 ATTACGTGAATCTAAATTTTT   1072
```

***Gallus gallus Rab6A* genomic region against *Drosophila dachshund_R65A11* CRE**

```
#=======================================
#
# Aligned_sequences: 2
# 1: gRab6A
# 2: dac_R65A11
# Matrix: EDNAFULL
# Gap_penalty: 16
# Extend_penalty: 4
#
# Length: 34
# Identity:      29/34 (85.3%)
# Similarity:    29/34 (85.3%)
# Gaps:           2/34 ( 5.9%)
# Score: 113
#
#
#=======================================

gRab6A         18338 AAAAAAAAAAAAAAACAAACAG--GGAGAAGAAA  18369
                     ||||||||||||||.||||.||  ||||.|||||
dac_R65A11       225 AAAAAAAAAAAAAACCAAAAAGTAGGAGGAGAAA    258
```

***Pan troglodytes DICER1* genomic region against *Drosophila dachshund_R65A11* CRE**

```
#=======================================
#
# Aligned_sequences: 2
# 1: PtDICER1
# 2: dac_R65A11
# Matrix: EDNAFULL
# Gap_penalty: 16
# Extend_penalty: 4
#
# Length: 261
# Identity:     148/261 (56.7%)
# Similarity:   148/261 (56.7%)
# Gaps:          24/261 ( 9.2%)
# Score: 144
#
#
#=======================================

PtDICER1       13219 GGATCTAGG-ATAAATTGTAATAACAGCAAAGTGAAATT---TTTTTAAA  13264
                     ||.|||..| |.||.||.|.||.. ||.|||.||.||.|   ||||.|.|
dac_R65A11       806 GGCTCTCTGTACAACTTTTCATTG-AGAAAAATGTAACTAGTTTTTCATA    854

PtDICER1       13265 GAAGAGCAAAAC--TCAAAGTCAAAACATCACATACTCTTATGCCTTTGG  13312
                     |.|..|.||.||  |..||..|||.| |||..|||.|.|.|| ||..|..
dac_R65A11       855 GCAACGGAATACAATTTAATCCAATA-ATCCAATAGTTTAAT-CCAATAC    902

PtDICER1       13313 AAAAGAAATAATAAAAATAGAAATTTGCCTCCATCAAAATTATAATACTA  13362
                     |||.||.||.|..|..||....|||.|.|..||....|||..|.|..|.
dac_R65A11       903 AAATGATATTACTACCATTTCTATTTTCGTTAATTTCGATTTGACTTATT    952

PtDICER1       13363 TTTCTGAATTCAGTG---AAAAGACAGTGTAATTAAAGGAATTAATTAAA  13409
                     |..|||.|||.|.|.   ||||.|.| |||.||.|     ||.|...|.|
dac_R65A11       953 TGGCTGGATTTACTTTTCAAAATATA-TGTTATCA-----ATAAGACACA    996

PtDICER1       13410 TATATCAAATATCTACTCTATTATAAACATACCAAGAAAATGAAACAAAA  13459
                     .|..|.|..|.||||. |||||   ||||||.....||||  |||.||||
dac_R65A11       997 AACCTTACTTTTCTAG-CTATT---AACATAGTTTAAAAA--AAAAAAAA   1040

PtDICER1       13460 AATTAATAAAA  13470
                     ||.||||||||
dac_R65A11      1041 AACTAATAAAA   1051
```

***Homo spaiens ERN1* genomic region against *Drosophila dachshund_R65A11* CRE**

```
#=======================================
#
# Aligned_sequences: 2
# 1: hsERN1
# 2: dac_R65A11
# Matrix: EDNAFULL
# Gap_penalty: 16
# Extend_penalty: 4
#
# Length: 332
# Identity:     176/332 (53.0%)
# Similarity:   176/332 (53.0%)
# Gaps:          34/332 (10.2%)
# Score: 124
#
#
#=======================================

hsERN1         36511 CATTGTTGACAATTTTTCCTTGTAGTTCAGTTTTTGCCTTATATATTTTG  36560
                     ||||.||.||..||..||.|..|.|.||.|.||..|..||..|.|....|
dac_R65A11       720 CATTTTTAACCGTTCCTCATGTTCGGTCCGGTTCGGTTTTCAAAACCGGG    769

hsERN1         36561 AAGCTATGTTGTTAG---GTGTCTAAGGATTCGTGCCTTATATGTTCTAT  36607
                     ||.|   ||..||||   |.||||....|||..||  ||.|. |.|||.|
dac_R65A11       770 AATC---GTACTTAGACTGGGTCTCCTTATTTCTG--TTCTG-GCTCTCT    813

hsERN1         36608 TCAGTTATTTTTTTTGATAAAAATGAAATATCTTTTTCTG----------  36647
                     ..|....||||..||||.|||||||.||....||||||..
dac_R65A11       814 GTACAACTTTTCATTGAGAAAAATGTAACTAGTTTTTCATAGCAACGGAA    863

hsERN1         36648 TCCCATTCAATCCTTTTCTTTTTGAATTCTGTCTATAGCCAGCCATAGCA  36697
                     |.|.|||.|||||..|..|.....|.||...||.|...|.|...|||..|
dac_R65A11       864 TACAATTTAATCCAATAATCCAATAGTTTAATCCAATACAAATGATATTA    913

hsERN1         36698 CTCTGAACATGCCTGATCTTGTCTGAATTCTATA-GA---ATCTGATTGG  36743
                     ||   |.|||..|| ||.||...|.|.|||.||. ||   ||.||..|||
dac_R65A11       914 CT---ACCATTTCT-ATTTTCGTTAATTTCGATTTGACTTATTTGGCTGG    959

hsERN1         36744 TATTGCTAAACCAGCATTTTGTTAATATTTGTTTGCTA--CATCTTTTTC  36791
                     ..||.||...|.|....|.|||||..|.|......|.|  |.|..|||||
dac_R65A11       960 ATTTACTTTTCAAAATATATGTTATCAATAAGACACAAACCTTACTTTTC   1009

hsERN1         36792 TATGTATTTA-----TTTAAAAAAAAAAAAAA  36818
                     ||..||||.|     |||||||||||||||||
dac_R65A11      1010 TAGCTATTAACATAGTTTAAAAAAAAAAAAAA   1041
```

***D. melanogaster dachshund* gene locus and homology to other insects**

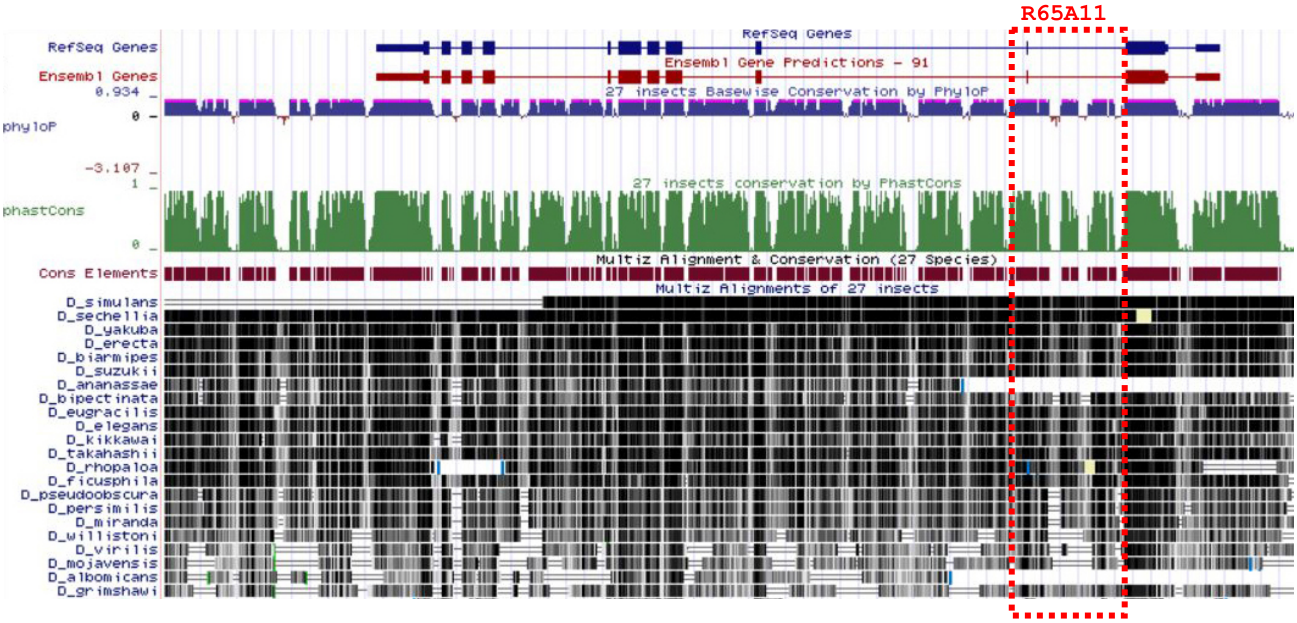

Image capture of UCSC Genome Browser on *D. melanogaster* Aug. 2014 (BDGP Release 6 + ISO1 MT/dm6) assembly, shown is the *dachshund* gene locus. RefSeq Genes shows exon (blue bars) and intron structure of dac; in red is shown Ensembl Gene annotation used for genome browsing. Green bar diagram indicates conservation among *Drosophilidae* species which are listed further below (black bars indicate sequence homology). Conserved elements are shown in dark red. Dashed red box indicates topology and extension of R65A11 CRE sequence determined in *Drosophila melanogaster*.

Examples of dac conserved sequence found in other *Drosophilidae*

***D melanogaster* scaffold**

TTGTTCGCAACCAACTAACAGAGGTTCGTCTCTAACATTTTTCAAAAAAATTACATAACTTTTAAATTTGATTTCAGTTTATTTGTAAG
TGAGAAGCCTATTTTCTAACCATAAATTCTGCACGTTAAGAGTATTTCCTTTCATATCGTATCTACAAAAATCAATCCAACACACCTGT
TTCATCTACCGTTAACACCGTTAAGCCCCGCCCCATTTTCTTATCGAAAATATAGCCCTTTTTCACGCTCTATTTATAGCATTCACATT
CTTTCTTTTTTTTTGCACTTTTTAGCTGGCATA**TCCTTTCGACTTCCGCCATTCGAGGCTCGCCCAATTTCCGTTTCGAGT**TTAATTAA
TTTAATAAACAAATTCTTTTCGCTCTAAAAACTCTCAAGTGTATCGATACGATGCGTTTCTTTTTTTCCTTCGTTAAATAAATAATAAC
CAAAAAAAAAAAAA**AACCAAAAAGTAGGAGGAGAAAAGTTATTGCCATAGTTTTTTTAT**TATACTTGTGTGTTTACCTTTCTGGTGGCTT
GATCGATAGGCATCTGCAATTAAAAAGAGAAGAAGAAGAGACAAGTGAGGCAAAATTGTTAAACGTTTTGTGTAAGCTTTAATACGAAA
AACAAGTACTGCAACATAACGGAAG

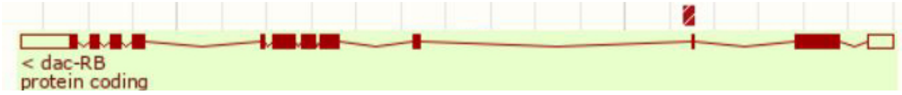

***D sechellia* scaffold**

>supercontig:GCA_000005215.1:scaffold_7:127389:128111:1
CATTGTTCGCAACCAACTAACAGAGGTTCGTCACTAATATTTTTCAAAAAACTTAATAATTTGCAATTTTTAATTCAGTTTATTTGTAC
TTGGGAAGCCTATTTTCTAACCATTTAATCTGCATGTTAAGAGTATTATTTCCTTTCATATCGTATTTACAAAAATCAATCCACACACC
TGTTTCATCTACCGTTAACACCGTTAAGCCCCGCCCCATTTTCTTATCGAAATAAAACCCTTTTTCACGCTCTATTTATAGCATTCACA
TTCTTTTTTTTTTTGCACTTTTTAGCTGGCCATA**TCCTTTCGACTTCCGCCATTCGAGGCTCGCCCAATTTCCGTTTCGAGTTTAATTAA**
TTTAATAAACAAATTCTTTTCGCTCTAAAAACTCTCAAGTGTATCGATACGATGCGTTTCTTTTTTTTCCTTCGTTAAATAAAATAATA
AC**AAAAAAACCAAAAAGTAGGAGGAGAAAAGTTATTGCCATAGTTTTTTTATTATACTTGTGTGTTTACCTTTCTGGTGGCTTGATCGA**
TAGGCATCTGCAATTAAAAAGAGAAGAAGAAGAGACAAGTGAGGCAAAATTGTTAAACGTTTTGTGTAAGCTTTAATACGAAAAACAAG
TACTGCAACATAACGGAAGGAAACAAGGCTTAAATTCGGGGCACAAATGCTGAAAGGGAAGTTTTTCATTGACGGGTTCGTTCTGACGG
ACTTGCATTTT

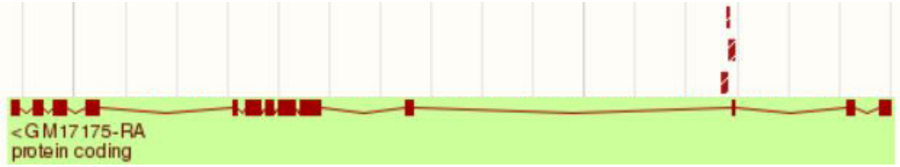

***D simulans* scaffold**

>chromosome:GCA_000259055.1:2L:16190558:16191295:1
AGGATCATTGTTCGCAACCAACTAACAGAGGTTCGTCACTAATATTTTTCCAAAAAATTAATAATTTGAAATTTTTAATTCAGTTTATT
TGTACTGGGGAAGCCTATTTTCTAACCATTTAATCTGCACGTTAAGAGTATTTCCTTTCATATCGTATTTACAAAAATCAATCCACACA
CCTGTTTCATCTACCGTTAACACCGTTAAGCCCCGCCCCATTTTCTTATCGAAATAAAACCCTTTTTCACGCTCTATTTATAGCATTCA
CATTCTTTTTCTTGCACTTTTTAGCTGGCCATA**TCCTTTCGACTTCCGCCATTCGAGGCTCGCCCAATTTCCGTTTCGAGTTTAATTAA**
TTTAATAAACAAATTCTTTTCGCTCTAAAAACTCTCAAGTGTATCGATACGATGCGTTTCTTTTTTTCCTTCGTTAAATAAAATAATAA
C**AAAAAAACCAAAAAGTAGGAGGAGAAAAGTTATTGCCATAGTTTTTTTATTATACTTGTGTGTTTACCTTTCTGGTGGCTTGATCGAT**
AGGCATCTGCAATTAAAAAGAGAAGAAGAAGAGACAAGTGAGGCAAAATTGTTAAACGTTTTGTGTAAGCTTTAATACGAAAAACAAGT
ACTGCAACATAACGGAAGGAAACACGGCTTAAATTCGGGGCACAAATGCTGAAAGGGAAGTTTTTCATTGACGGTTTCGTTCTGACGGA
CTTGCATTTTGGCGGGCAAGCGGGTG

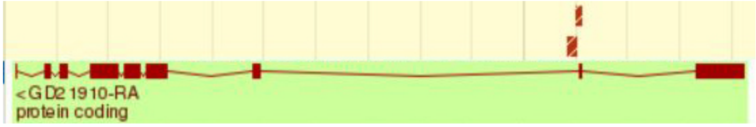

***D yakuba* scaffold**

>chromosome:GCA_000005975.1:2R:2939300:2940051:1
GAGGATCATTCTTCGCAAACAATAGAGGTATGTGTAGGTCTCTAACATTTTTCAAAATATTACTCTATTTAAAAGTGTAAATTCGGTTT
ATTTGTGTGTAACTAGCCTATCTTCTAACCATTTCTTCTGGACGTTAAGAGTATTTCCTTTCATATCGTATCTCCAAAAATCAATCCAC
ACACCTGTTTCATCTACCGTTAACACCGTTAGGCCCCGCCCCATTTTCTTATCCAAATAAAACCCTTTTTCACGCTCTATTTATAGCAT
TCACATTCTTTTTTGCACTTTTTAGCTGGCATA**TCCTTTCGACTTCCGCCATCCGAGGCTCGCCCAATTTCCGTTTCGAGTTTAATTAA**
TTTAATAAACAAATTCTTTTCGCTCTAAAAACTCTCAAGTGTATCGATACGATGCGTTCATTTTTTTCCTTCGTTAAATAAATAATAAC
AAAAAAAGAACCAAAAAGTAGGAGGAGAGAAATTATTGCCATAGTTTTTTTATTATACTTGTGTGTTTACCTTTCTGGTGGCTTGATCG
ATAGGCATCTGCAATTAAAAAGAGAAGAAGAAGAGACAAGTGAGGCAAAATTGTTAAACGTTTTGTGTAAGCTTTAATGCGAGAAACAA
GTACGGCAACATAACGGAAGGAAACACGGCTTAAATTCGGGGCACAAATGCTGGAAGGGAAGTTTTTCATTGACGGCTTCCTTCTGACG
GACTTGCATTTCGGCGGGCAAGCGGGTGTGAAAATGCACA

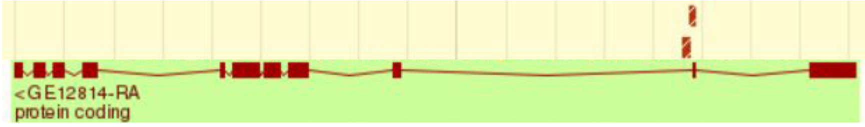

***D ananassae* scaffold**

>supercontig:GCA_000005115.1:scaffold_12916:16070598:16071275:-1
AAAATGATCATTGGGTTGGCTGCCATTTGAGAAGCCCCCAATAAAGGCAAACAAATATAATAAAAAATTATTGCAACGTAAGTTTTGAA
TACAATTGGACGGTCTCAAGAGTGTTTAGAGTGAAAAACAATAAATGGTGTTAATTTTTGTTCAGTTTTTAAAATCAAACTCAATACCT
GTTCCATCTATATACCTTTCATATCGATCCCCTTGCTCTAATAATCATGCTATATCATATTTTTTTATGTTTCATGCTCTATTTATCCT
TCCGGGCTTATCCTTTCGACTTCCGGGACCCTG**GAGGCTCGCCCAATTTCCGTTTCGAGTTTAATTAATTTAATAAACAAATTCTTTTC**
GCTCTAAAAACTCTCAAGTGTACAAACTTTTTTACTGTTTTTTTGTTACCTTTAAAAAACAACGATAACCGAAAAAATTTATAAACAAA
AAATTTTCTTAGGGGGGGAAATAGGAGCGAAA**TTATTGCCATAGTTTTTTTATTATACTTGTGTGTTTACCTCTCTGGTGGCTTGATCG**
ATAGGCATCTGCAATTAAAAAGAGAAGAGAAGAGACAAGTGAGGCAAATTGTTAAACGTTGTGTGTAAGCTTTTAATACAGTGAGAGAA
AAGAAAAACAGGAAACCAAAAAAAACAATATTCAGAGGGATGGTATTATGCTGCA

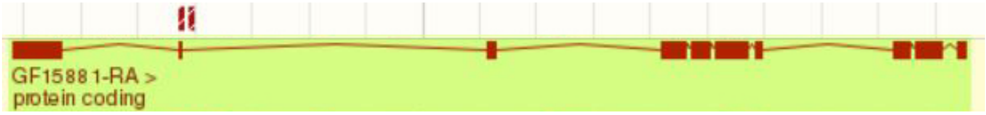

***D pseudoobscura* scaffold**

>supercontig:GCA_000001765.2:4_group3:8197417:8198097:1
AGATCTGTTCCCAGTTGTTCAAACTAAAAGTTAAGCTCAAGTTAAAAATGATCAAATTACTCCCAGACGGTATTGGATTTCCTGTCTCC
CCTTGGTTTCGACCATAGATTATCTTTCAGAACCGTTTCAATCGCTTTATCTGCGTATAAAAAATCCAATCCCCTCACCTGTTCGCTCT
ACTGTTAACACCGTTATAACCGTAATTCCCAAAAATGAAACCCAAAAATTCTTATCGGATAAACCCTTTTTTCATGCTCTATTTCTTGT
TTAACCCTCAAATATCCTTGCAACTTCCGCATT**TTCGCGGCTCGCCCAATTTCCGTTTCGAGTTTAATTAATTTAATAAACAAATTCTT**
TTCGCTCTAAAAACTCTCAAGTGTAGGAAAAAAACGATGCAATCGATGCGTTTCTTTCTTCCAAAAAACAAAAAAACCAAAAAATATTA
AAAAAAAAAAAGAGT**AAAAGGAGGAGGAGAGAAATTATTGCCATAGTTTTTTTATTATACTTGTGTGTTTACCTCTCTGGTGGCTTGAT**
CGATAGGCATCTGCAATTAAAAAGAGAAGAGAAAGAGACAAGTGAGGCAAAATTTGTTAAACGTTGTGTGTGTGTAAGCTTTAAACAAC
AAAAGTAAAAAACGAGTGAAACTCTGAATAAAACAGAGGAGTTCCGTCCCCGAAAACA

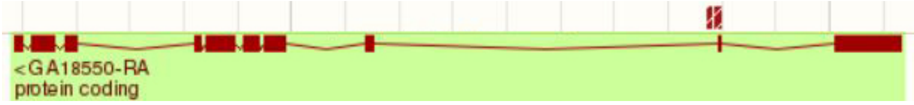

***D willistoni* scaffold**

>supercontig:GCA_000005925.1:scf2_1100000004585:4705370:4706001:1
AGAAGGCCAGCGTAACGAAATTATAACATTTTCATGCTCTATTTACGTATCCTTCGATTTCAAAAATTTGTGAATGGACTCTCCCATTT
CCGTTTTTTTTTTTTGTTTATGTAAAATATAATTTAATTTAATAAGAAAAAACTAAATTTG**TTTTCGCTCTAAAAACTCTCAAGTGT**TT
TGTGTGTATATAGAAAAAAAATGAATGTATACAATATGCGATCGATTTTTCATAACGGAAAAGATATTATTGCCGTAGTTTGTTTTTGT
TTTTTTTTTTTG**TTTTTTATTATACTTGTATGTTTTACCTCTCTGGTGGCTTGATCGATAGGCAT**CTGCAATTAAAAAGAGAAGAAGAA
GAGACAAGTGAGGCACAACATTTTTGTTAAAACATTACAACAAAAAAAAAAGGAGAACATAATGTAATAAGTAAAAGAGAAAATATTAC
ATAGGGATGTATTTTAGACGGAAGTTTCGGGGGCAAAAGCGGGTGTGAAAATGCACACGCCCCTTACCCCCGCTCCCCTTTCCCCGGCA
CATTCCTTCCCCTCCTCTGACGTCTGTCTATCTTACTGAGGTCCCTCATAATTATTATGGCCAGGGCCCAAACAGTGGGGTGCAACTTC
GACCTACCA

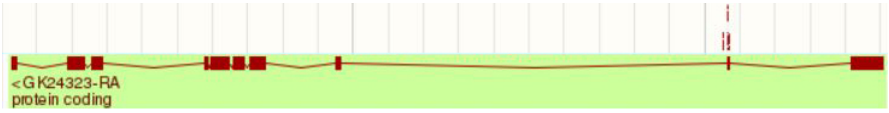

D grimshawi scaffold

>supercontig:GCA_000005155.1:scaffold_14978:688467:689119:1
CTCGCTTTGACCCACATCTTTTCGCATAGCTGGCCAAACGCGTTATCCTTAACCAAAAGCAACAACAAAAAAGAAAAAGAAGAGGAAAA
AAAGCCCATTAACACGCTCTATTTGTTTACGTTCCCAAAAAACTTCCGGCTGTCGTACGCTGGCTCGTCCCATTTCCGCATTAAGTAAT
TAATTTAATAACAATTTTTTATTTTTTGTTTTTTT**TTTTCGCTCTAAAAACTCTCAAGTG**CACACAGAACCGAGTACATCGATTGTAGG
ATCGATTTCGATTTCAACAGATTGCCATAGTTT**TTTTTTTATTATACTTGTGTGTTTACCTCTCTGGCGGCTTGATCGATAGGCAT**CTG
CAATTAAAAAGAGAAGAACAAGAGACAAGAGTGAAGTATAAAAAACACATAGGTAAAAAACGTTGTATGTGTGTGTGTGGTTTGTTGTG
TGTGTGTGTGTTAAGCTTCGACTAAAACAAAAACAAAAACAGCAACAAAAAACAAAAAAAAAACACGATCAGAAAAAAGAAAACGCAAT
TTTAAAAATTTCAAAATTATGTTTCACTTATGGACGGCACGGCGGGAAAGCGGGTGTGAAAATGCACACGCCCCAACCCCTTCAGAAAA
AAAAAAGAGACGCGGGCAGCCACGATAACT

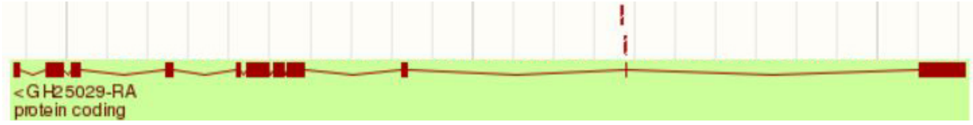

dachshund-DACH1 conserved CRE - potential transcription factor binding sites

dachshund, *mDach1* and *hDACH1* conserved sequence alignment was generated using MLAGAN (http://genome.lbl.gov/vista/lagan/submit.shtml). Potential transcription factor binding sites were identified using JASPAR (http://jaspar.genereg.net/); potential binding sites are color coded as per legend below.

Seq1 = *dach* supercons; Seq2 = *mDAach1* supercons; Seq3 – *hDACH1* supercon.

```
sequence1(+)    000000001  TCCTTTCGACT---------------------------------------  000000011
sequence2(+)    000000042  TCCATTAGACTGAC-TTTCTTGCCCTTTGTAGCACATTTGTATTCTGTGG  000000090
sequence3(+)    000000042  TCCATTAGACTGACTTTTCTTGGCCTTTGAAACACATTTGTATTCTGCAG  000000091

sequence1(+)    000000012  -------------------------TCCGCCATTCGAGGCTCGCCCAATT  000000036
sequence2(+)    000000091  AG-AAAAGGAAAATTGATCCCTGAGGCCACTAATGGAAACACTTTCTATA  000000139
sequence3(+)    000000092  AGAAAAAGAAAAATTGATCCTTGAGGCCATTAATGGAAACACTCCCTATA  000000141

sequence1(+)    000000037  TCCGTTTCGAGTTT---AATTAATT-------------------------  000000058
sequence2(+)    000000140  CCAGTTTTCAGTTTTTCAATTGATTGAATTAGATTTTAGAAATGTTTAGA  000000189
sequence3(+)    000000142  CCAGTTTTGAGTCTTTCAACTGATTGAATTAGATTTTTTAAATGTTTGTA  000000191

sequence1(+)    000000000  --------------------------------------------------  000000000
sequence2(+)    000000190  CTAACAATATATGAGTTTAACCGAAAAATAGAAGAAGAAAAACAGCCAAG  000000239
sequence3(+)    000000192  CTAACAACATATGAGTTTAACCGAAAAATAGAAGAAGAAAAACAGCCAAG  000000241

sequence1(+)    000000059  -------TAATAAACAAATTCTTTTCGCTCTAAAAA--------------  000000087
sequence2(+)    000000240  AAACTGGTAGTAAATGAGCCTTTATTGCCTCAGGCATTGGCCTAGTTAAT  000000289
sequence3(+)    000000242  AAACTGGTAATAAATGAACCTTTATTGCCTCAGGCATTGGCCTAGTTAAT  000000291

sequence1(+)    000000088  -------------------CTCTCAAGTGT--------------------  000000098
sequence2(+)    000000290  AAACTTTCATTGAAGGTTTCTTTCAACTGTTACGGACAATGAGAGGGGGA  000000339
sequence3(+)    000000292  AAACTTTCATTGAAGGTTTCTTTCAACTGTTACGGACAATGAGA--GGAA  000000339

sequence1(+)    000000099  -----------ATCGATACGATGCGT-----------------TTCTTTT  000000120
sequence2(+)    000000340  AAAAATCTTAAATTACAGCCATGTGTGACTTTTAAAGCAGTCATTCTTTT  000000389
sequence3(+)    000000340  AAAAATATTAAATTACAGCCATGTGTGACTTTTAAAGCAGTCATTCTTTT  000000389

sequence1(+)    000000121  T---------------TTCCTTCG--------------------------  000000129
sequence2(+)    000000390  TTATCTCAATACAACATTCCTTTGTCTAGCATCTCCATGAAAATTATCAT  000000439
sequence3(+)    000000390  TTATCTCAATACAATATTCCTTTGTCTAGTATTTCCATGAAAATTATCAT  000000439

sequence1(+)    000000130  ----------------TTATTTAAATAATAA---------------CCAAA  000000149
sequence2(+)    000000440  TTGAAAGGTTTATATCTTAAATAAAGGGGAAAAAACCATTTTCTTCTAAA  000000489
sequence3(+)    000000440  TTGAAAGGTTTATATCTTAAATAAAGAAAAAAG------TTTCTTCCAAA  000000483

sequence1(+)    000000150  AAAAAAAAAAACCA------AAAAGTAGGAGG----AGAAAAGTTATTGC  000000189
sequence2(+)    000000490  TACAATTAAAACTAGTTT-GAAAAGTAAGAGGCTCAGGAAACAAGATCTG  000000538
sequence3(+)    000000484  TACAACAAAAACTAGTTTAGAGAAATAGTATGCTGAATAAATGAGATCTA  000000533

sequence1(+)    000000190  CATAGTTTTTTTATTATACTTGT----GTGTTTACCTTTCTGGTGGCTTG  000000235
sequence2(+)    000000539  AATAAGTAATGTATTA---------GCAGGTAGAACATCTTGAAAACTT-  000000578
sequence3(+)    000000534  AA-----AATATGCTACAACCATATTCATATGTAACATC-TGAAAACCT-  000000576

sequence1(+)    000000236  ATCGATAGGCAT  000000247
sequence2(+)    000000579  ACTAAGAATGCT  000000590
sequence3(+)    000000577  ATTAAGAGTGTT  000000588
```

TAATAAA = *Distalless (Dll)*
TCTCAAGTG = *ventral nervous system defective (vnd)*
CGATAC = *sine oculis (so)*
TTATTTA = *unplugged (unpg)*
ATAATAA = *Sex combs reduced (Scr)* as well as *empty spiracles (ems)*
TTTATTA = *invected (inv)/engrailed (en)*, as well as *unpg* and *Scr*
